# Rongalite addition to dienones: diastereoselectivity in cyclic sulfone synthesis; stereochemical rationalization and prospects as a general conjugate nucleophile

**DOI:** 10.3762/bjoc.22.56

**Published:** 2026-05-13

**Authors:** Melina Goga, Hao Zong, James Franco, Jazmine Prana, Rudolph Michel, Antonia Muro, Elana Rubin, Janet Brenya, Henk Eshuis, Magnus W P Bebbington

**Affiliations:** 1 Department of Chemistry and Biochemistry, Montclair State University, 1 Normal Avenue, Montclair NJ 07043, USA

**Keywords:** diastereoselectivity, Rongalite, sulfones

## Abstract

A detailed experimental and computational study of the diastereoselectivity of cyclic sulfone synthesis by reaction of Rongalite with doubly electrophilic dienones is presented. Computational methods, including density functional theory, conformational search methods, and internal reaction coordinate methods, have been used to rationalize the diastereoselectivity of the reaction by transition-state modeling, and showcases the conformational subtleties of sulfur-containing rings. Extension of the original reaction to double intermolecular additions is also demonstrated along with a comparative study of Rongalite with the reactivity of other readily available sulfinate nucleophiles using competition and exchange experiments.

## Introduction

Sulfonyl groups are widespread in licensed drugs [1]. New methods for the incorporation of sulfonyl groups into organic molecules are desirable, particularly in the light of the general need for more sustainable chemical synthesis. Recently, there has been significant progress in the use of SO_2_ equivalents and bulk inorganic high oxidation state sulfur compounds for the direct incorporation of sulfones [2–3]. Despite these advances, use of a low-valent sulfur nucleophile for initial installation of the sulfur atom followed by oxidation remains a widespread method, even though it is far from ideal environmentally and also in terms of functional group tolerance [4].

**Figure 1:**
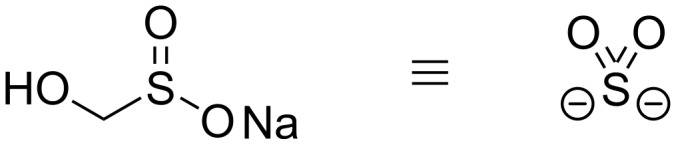
Rongalite as an equivalent of the unstable hyposulfite ion.

Rongalite^TM^ is a low-cost commodity chemical that has been used as a bleaching agent in the dyeing industry for more than a century [5–6]. Over the last 10 years particularly, it has begun to join the toolbox of reagents in organic synthesis, and shows diverse reactivity. It has been employed in reductions, radical processes and as a reagent for C1 transfer [7–14]. Importantly for our work, it can also act as an equivalent of the unstable hyposulfite (SO_2_^2−^) ion (Figure 1) [15–16].

**Scheme 1:**
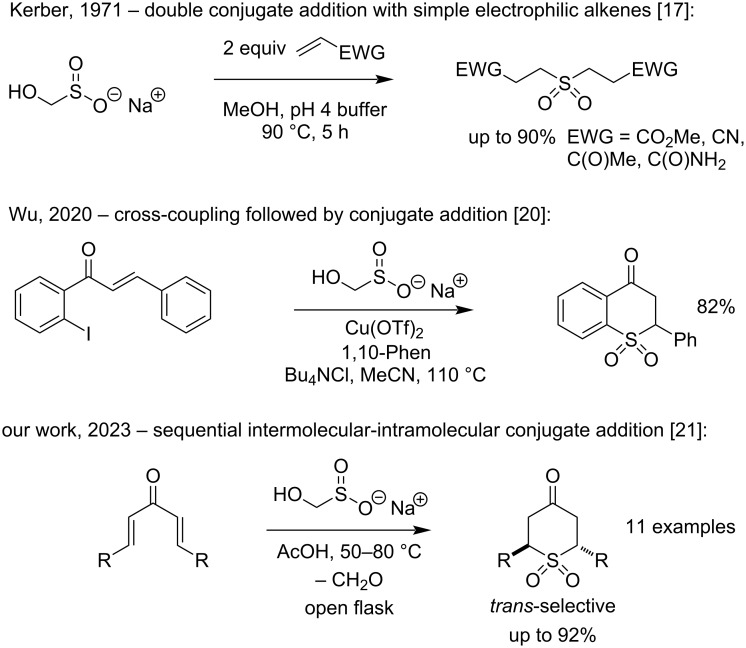
Progression of Rongalite conjugate additions.

The reagent was first used for the preparation of symmetrical sulfones as early as 1971 [17], but interest in the reagent has increased dramatically over the last two decades [5–6]. Modified reagents in which the Rongalite hydroxy group has been protected have allowed for consecutive and different reactions at sulfur, giving access to a range of unsymmetrical sulfones [18–19]. Although sulfur nucleophiles often participate in conjugate additions, few reports documented this behavior for Rongalite (Scheme 1). This may have been due to its known reactivity as a conjugate reductant [17,20]. We recently published a preliminary report on the reaction of Rongalite with dienones leading to cyclic sulfones (Scheme 1) [21]. We now describe our efforts to resolve a stereochemical ambiguity, the modelling of transition states to explain the diastereoselectivity of the one-pot reaction, a full exploration of its scope and limitations and further mechanistic and competition experiments that place the nucleophilicity of Rongalite in context by comparison with other readily available sulfinates.

## Results and Discussion

### Confirmation of diastereoselectivity

**Figure 2:**
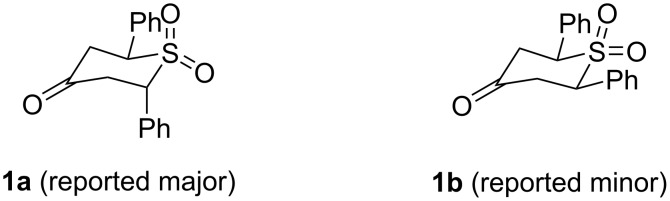
Sulfone diastereomers **1a** and **1b**.

In our initial report, we supposed that the major product was the *trans*-isomer, following the analysis of Detty and co-workers [22], who prepared sulfones **1a** and **1b** (Figure 2) by a conventional sulfide oxidation. In that early work, the stereochemical analysis was dependent on conformational analysis and comparison of coupling constants in the ^1^H NMR spectra. Experiments discussed in our inital report confirmed that the reaction proceeded under kinetic control [21].

**Scheme 2:**
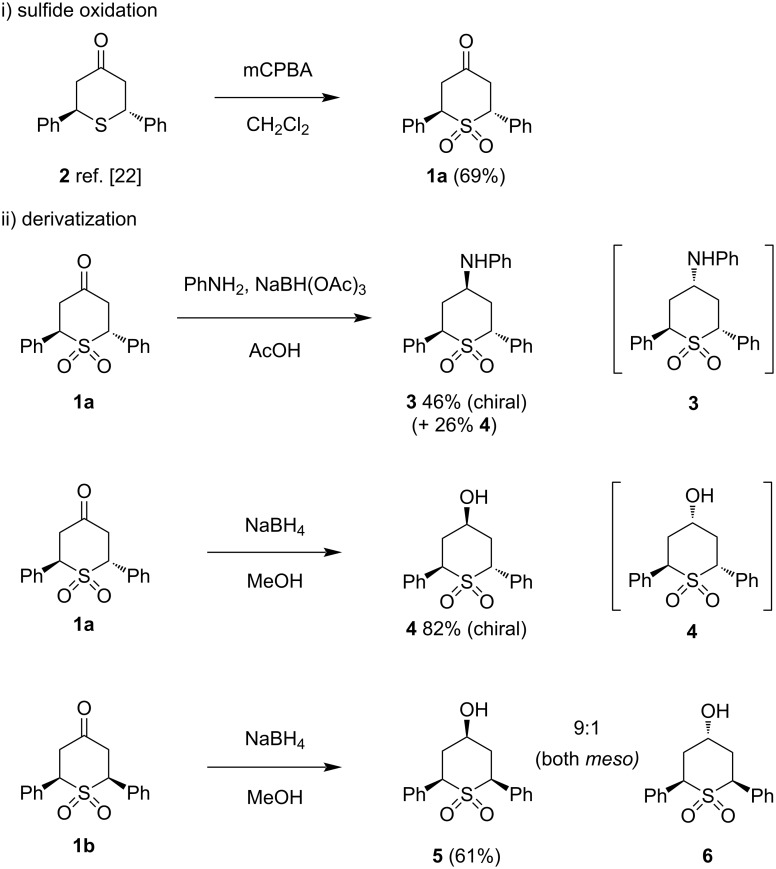
Verification of relative stereochemistry by i) sulfide oxidation and ii) derivatization.

Closer examination of the 1995 paper [22] revealed a slight ambiguity – the ^1^H NMR signals that were discussed and used to assign relative stereochemistry in the main text were found to be swapped over in the experimental section. We had no reason to think that this was anything but a typographical error, but we nevertheless sought confirmation of the structures of the minor and major diastereomers in our own hands. Noting that preparation of sulfides by double conjugate addition of Na_2_S or NaSH has been widely studied, and that the stereochemical assignments presented therein are in some cases supported by X-ray crystallographic studies, we prepared *trans*-sulfide **2** using an established protocol [23], and then oxidized it to *trans*-sulfone **1a** (Scheme 2). This material was identical to the major product from Rongalite double conjugate addition.

Additional confirmation of the structures of major and minor diastereomers **1a** and **1b** was provided by derivatization of both molecules in ways that confirmed their relative stereochemistry by taking advantage of the different symmetry elements present in each case. Thus, both reductive amination and hydride reduction of *C*_2_-symmetric **1a** led to the isolation of the only possible (chiral) diastereomer of the products **3** and **4** in each case – reaction of the nucleophile with either face of the iminium ion or ketone gives the same diastereomer, as illustrated. By contrast, hydride reduction of **1b** gave an approx. 9:1 mixture of products **5** and **6**, as judged by the crude ^1^H NMR spectrum. The major product was isolated and assigned as **5** (from axial attack of a small hydride reagent) [24]. From analysis of the NMR spectra of **5**, it was clear that the molecule retained symmetry following reduction, reflected in the reduced number of signals in both ^1^H and ^13^C spectra compared to alcohol **4**.

### Steric limits and advanced computation

**Scheme 3:**
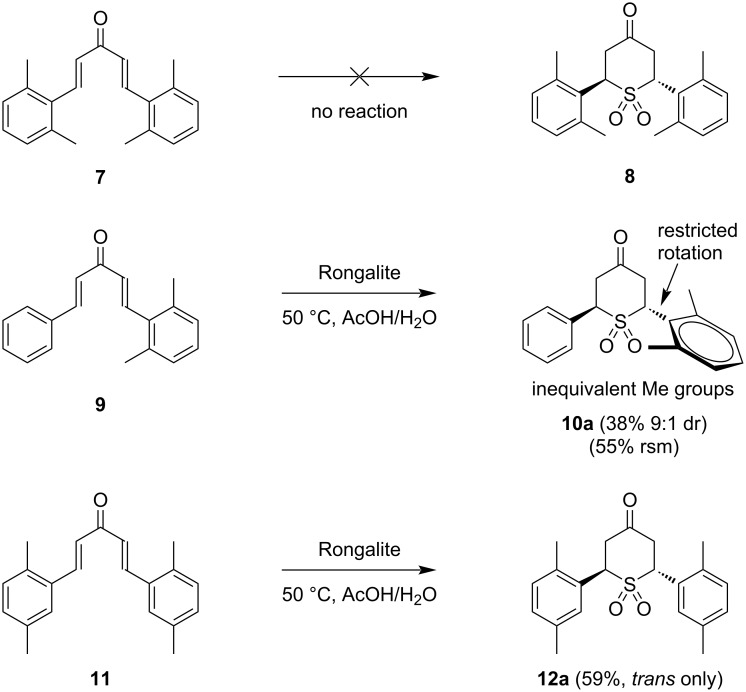
Reactions of sterically hindered substrates.

**Figure 3:**
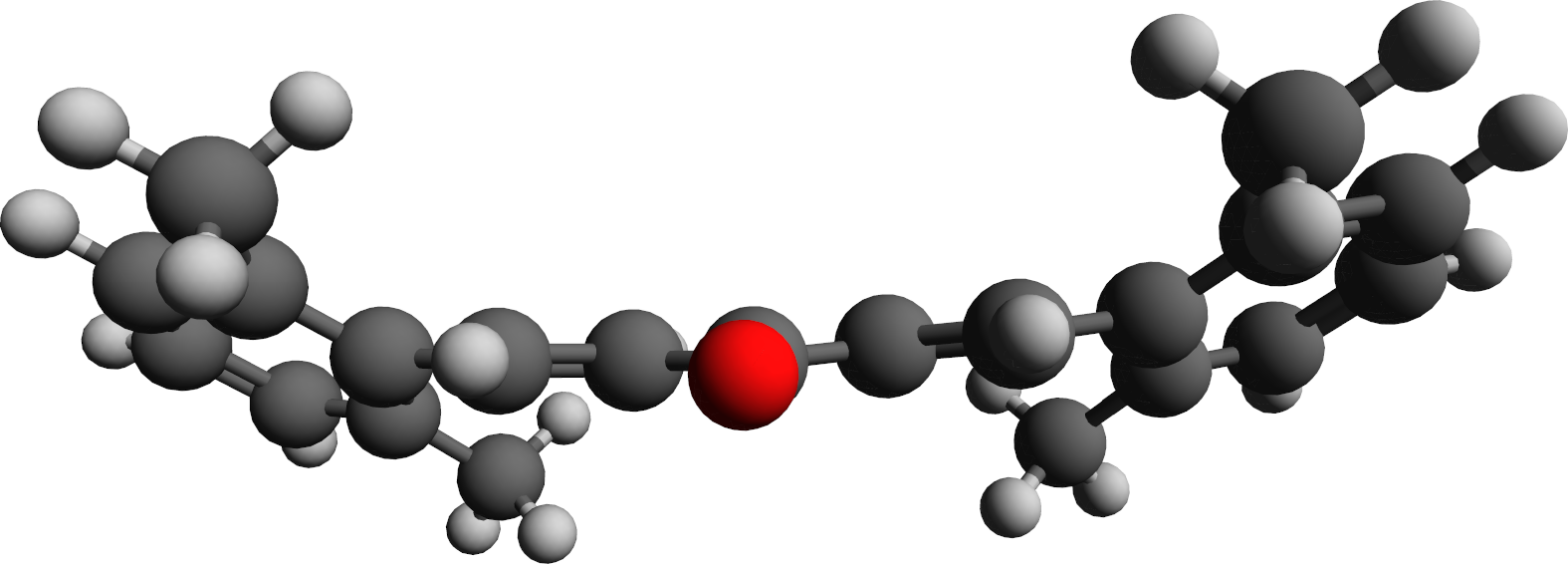
Minimized structure of dienone **7** (calculated using the PBE0 functional [26] and the def2-SVP basis set [27] (see Supporting Information File 1 for full computational details)).

Substrate **7** (Scheme 3), with four *ortho*-methyl groups, showed no sign of product formation under the standard reaction conditions. In order to determine whether this was due to an unreactive conformational preference of the substrate or to difficulty in forming the product, we used density functional theory (DFT) and conformational search techniques based on tight-binding methods to find the energetically minimized structure of **7** (see Supporting Information File 1 for details on all computations). The lowest energy structure is depicted in Figure 3. It shows a structure somewhat rotated out of planarity (dihedral angles between aromatic C–C and vinylic CH are 34°). This presumably still allows for some conjugative stabilization from the aromatic rings to the enone π-systems and reduces eclipsing interactions between the *ortho*-methyl groups and the vinylic H (indicated). There is nothing to suggest from the calculations that an inherent reactivity problem prevents the initial addition of Rongalite to the enone, especially given that the singly electrophilic 2,6-dimethylbenzalacetone is known to participate in reactions with conjugate nucleophiles [25]. It is therefore more likely that steric effects in the transition state for the putative cyclization to give **8** are prohibitive. Indeed, we were unable to locate a transition state for this process computationally.

In order to test the limits of the reaction with related substitution patterns in this process, we prepared unsymmetrical substrate **9** from benzalacetone and 2,6-dimethylbenzaldehyde. Reaction of **9** with Rongalite under our standard conditions did indeed give the expected product **10a**. In this case only the major (trans) isomer was isolated, but the minor diastereomer did appear to be present in the crude reaction mixture (≈9:1 ratio by ^1^H NMR). Evidence for the sterically congested nature of the sulfone **10a** was provided by the NMR spectra, which show evidence of restricted rotation (Scheme 3). In the ^1^H NMR spectrum the signals for the two *ortho*-methyl groups are inequivalent, and indeed differ in chemical shift by a remarkable 1.0 ppm. Similarly, in the ^13^C NMR spectrum, the *ortho*-disubsituted ring produces 6 aromatic and 2 aliphatic signals, rather than the 4 aromatic and 1 aliphatic signals that would be expected given the symmetrical aromatic substitution pattern. These data indicate that the *ortho*-methyl groups cannot freely rotate past the nearby sulfone oxygen atoms. Calculations suggested a rotation barrier of 18 kcal/mol, consistent with it being insurmountable at room temperature, vs only 4 kcal/mol for the unsubstituted phenyl ring (see Supporting Information File 1).

**Figure 4:**
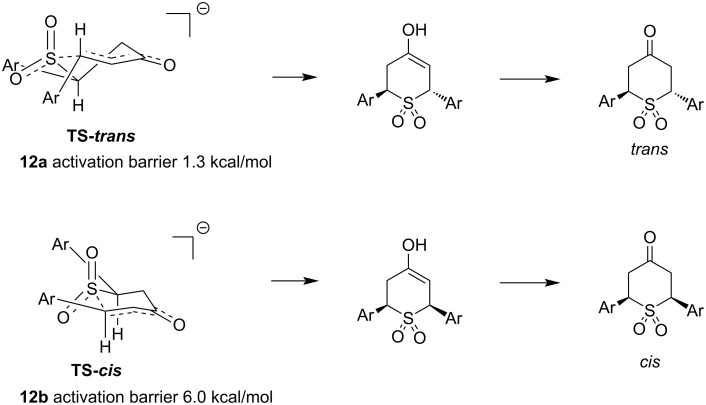
Modeling of cyclization transition states (the solvating acetic acid molecules were omitted for clarity, and the curvature of the 6-membered rings is exaggerated for the same reason. Calculated using the PBE0 functional [26] and the def2-SVP basis set [27] (see Supporting Information File 1 for full computational details)).

The combined results of substrates **7** and **9** (Scheme 3) suggested that a maximum in permitted steric congestion might be demonstrated by reaction of dibenzalacetone substrate **11** [28] with Rongalite. Pleasingly, this reaction was successful, leading to adduct **12a**. Notably, in this case, no trace of the minor isomer was observed in the crude ^1^H NMR spectrum (Supporting Information File 1). We thus used computation to model the cyclization to give products *trans*-**12a** and c*is*-**12b**, as this substrate appeared to represent a limiting case for both reaction diastereoselectivity and steric tolerance. Given that the reaction takes place in a high concentration of ≈80% acetic acid (pH ≈3), it seemed plausible that activation of the dienone carbonyl group would occur by hydrogen-bonding, rather than complete proton transfer to the carbonyl oxygen lone pairs [29]. We therefore included explicit solvation with acetic acid in our computational work, by which we were able to locate transition states for reactions to give *trans*-**12a** and the unobserved *cis*-**12b** (Figure 4). The activation energy difference to reach **TS-12a**, leading to **12a** and **TS-12b**, leading to **12b** was found to be a significant 4.7 kcal/mol in favor of **TS-12a** (see Supporting Information File 1 for a graphical representation of this and TS images directly generated from the computational data). The analogous calculations for **1a** and **1b** showed a much smaller difference in activation barrier, 1.0 kcal/mol, which is consistent with the lower selectivity observed for that substrate, within the expected accuracy of the calculations (see also Supporting Information File 1) [30]. Similar transition states have been invoked for other cyclizations leading to 6-membered rings [31–33], but this is the first time that they have been computationally modeled for a sulfur nucleophile in a reaction of this type. What follows is an examination of the transition state structures leading to the *trans* and *cis*-isomers **12a** and **12b**; although the transition states leading to **1a** and **1b** may differ slightly in energy, they are very similar in appearance to those for **12a** and **12b**. Examination of the calculated transition state structure **TS-12a** shows a flattened half-chair conformer, which has the aryl rings in *pseudo*-equatorial positions. **TS-12b** adopts a different shape, closer to a flattened chair which again allows both aryl groups to be equatorial. There has been much debate in the literature regarding the conformational preferences of sulfur-containing 6-membered rings – spectroscopy of structurally related 5,6-dihydro-2*H*-thiopyrans suggests a preference for a half-chair in the unsubstituted ring but there is some evidence for boat-like major conformers in substituted systems [23,34–36]. There are clearly some subtle interactions that contribute to the observed energy differences between the differently shaped two transition states, but undoubtedly their aggregation leads to a significant overall energy difference. Although one of the aryl rings is close to eclipsing one of the S–O bonds in **TS-*trans***, there is a significant distortion in the chair shape in **TS-*cis***: the two highlighted CH bonds are not parallel – they are converging and this 1,3-diaxial interaction may be a contributing factor in increasing the activation barrier relative to **TS-*trans***. Further technical discussion of the calculations is provided in Supporting Information File 1.

### Current limitations

**Figure 5:**
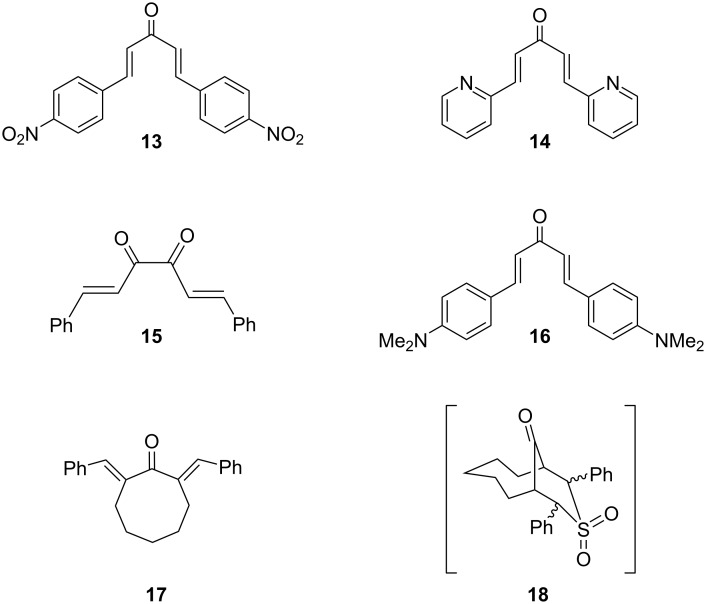
Unsuccessful substrates.

Shown in Figure 5 are some substrates that were not successful, which serve to inform the scope and current limitations of this process. Substrates **13**, **14**, and **15** [33,37–38] are all very electron-poor and resulted in unidentifiable mixtures. This is most likely due to decomposition processes initiated by reduction. Rongalite has been reported to react in this manner with other electron-poor aromatics [39]. Substrate **16** [40], in contrast, is very electron-rich. This produced sluggish reactivity, incomplete conversion and unknown products. Typical signals for the *trans*-product could be seen in the crude ^1^H NMR spectrum, but we were not able to isolate this compound in sufficient purity for characterization. We were intrigued to study the cyclooctanone-derived substrate **17** [41], as it would give an interesting bicyclic system **18**. However, we did not obtain any of the desired product, with low conversion and a mixture of products, none of which were sulfone **18**. The absence of cyclized product is likely due to the necessity of forming a bridgehead enol intermediate which violates Bredt’s rule [42]. A larger ring substrate may have circumvented this problem, but the necessary double aldol reactions to prepare such substrates, using cyclononanone or cyclodecanone are not known. The difficulties in forming medium-ring ketone enolates have been well described [41].

### Rongalite reactivity vs other sulfinates

We finally decided to place the reactivity of Rongalite in context by comparison with other commonly available sulfinates, namely, sodium methanesulfinate and sodium *p*-toluenesulfinate.

**Scheme 4:**
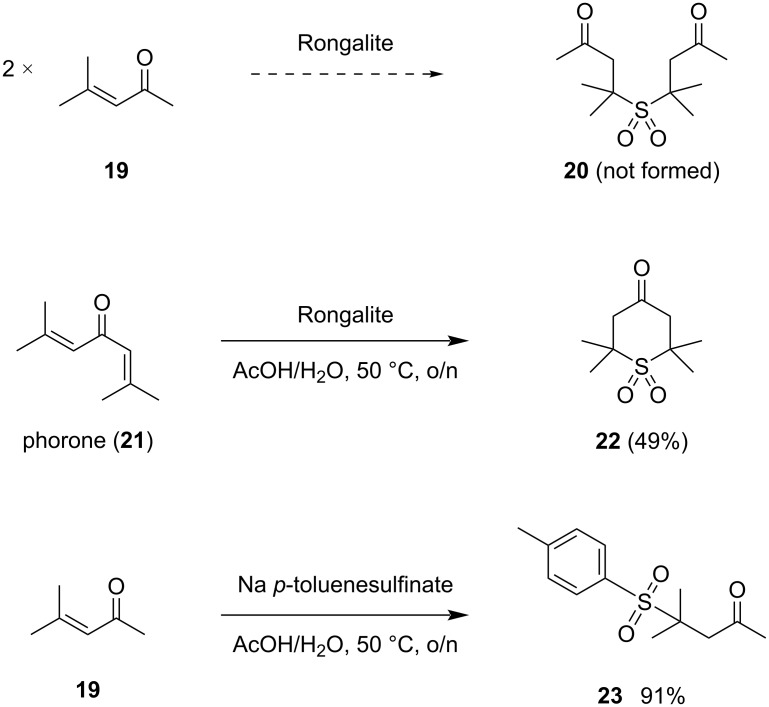
Initial reactivity comparison with *p*-toluenesulfinate.

In the course of our previous studies, we had established that Rongalite did not react productively with mesityl oxide (**19**) to give **20**, in contrast to the successful reaction with phorone (**21**) to give **22** [43] (Scheme 4). As a control reaction, we conducted the addition of commercially available sodium *p*-toluenesulfinate to mesityl oxide under analogous conditions, which produced known sulfone **23** [44] in high yield. This demonstrates the general viability of sulfinate addition to produce *C*-tertiary sulfones, but also implies steric limits to these processes in double intermolecular conjugate addition with Rongalite as the nucleophile.

**Scheme 5:**
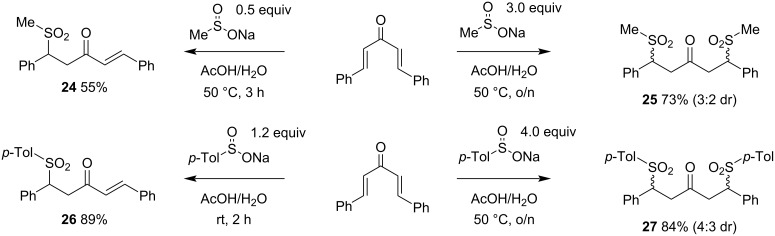
Preparation of anticipated products in competition experiments with Rongalite and other sulfinates.

Our approach was to use competition experiments to indicate the relative reactivity of Rongalite to related aryl- and alkylsulfinates, so that future predictions will be possible about its reactivity with other electrophiles. In anticipation of the likely products from such test reactions, we prepared the mono- and diadducts **24**–**27** arising from addition of methanesulfinate and *p*-toluenesulfinate to dibenzalacetone (Scheme 5). Diadducts **25** and **27** were isolated as inseparable 3:2 and 4:3 mixtures of diastereomers, respectively. From these experiments, we observed the following: 1) double conjugate addition of methanesulfinate occurs readily, such that selective preparation of monoadduct **24** requires an excess of dibenzalacetone to prevent double addition; 2) the second addition of *p*-toluenesulfinate is significantly slower than the first – however this could be explained by the crystallinity of the monoadduct **26**, which precipitates much more readily from the reaction mixture than does **24**. A longer reaction time and an excess of sodium *p*-toluenesulfinate is needed to convert **26** to **27** in a high yield; 3) both reactions are cleaner and higher yielding in the case of *p*-toluenesulfinate addition.

**Scheme 6:**
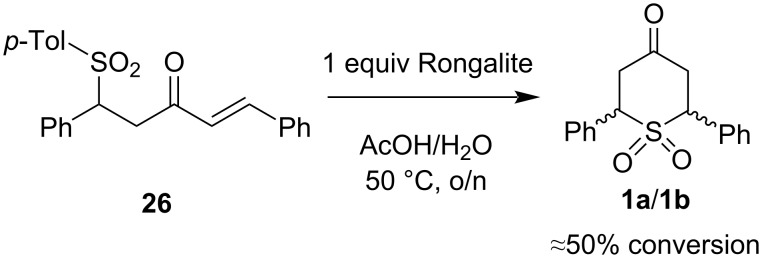
Exchange experiment.

Competition experiments were then performed by treatment of dibenzalacetone with 1 equivalent of methanesulfinate or *p*-toluenesulfinate and 1 equivalent of Rongalite (50 °C, AcOH/H_2_O, o/n) led to a mixture of products. These results are tabulated in Table 1. Mixtures of cyclic and acyclic products were obtained in both cases. Our initial interpretation was that the ratios reflected the relative rates of reaction of Rongalite and the other sulfinates. However, we also considered the possibility that initial sulfinate addition could be reversible. To test this hypothesis, we treated sulfone **26** with Rongalite under standard conditions (Scheme 6) and found approximately a 1:1 mixture of **26** and the cyclic sulfones **1a/1b** in the same diastereomeric ratio (≈7:1) as observed earlier. This supports the idea that sulfinate addition is reversible. Adducts **1a** and **1b** most likely form from dibenzalacetone generated in situ. Therefore, prior elimination of *p*-toluenesulfinate to give dibenzalacetone, followed by Rongalite addition, would account for the formation of the cyclic products here. This reaction occurs to a significant extent on the timescale of the competition experiments at 50 °C, the results of which cannot therefore be fully reflective of the relative nucleophilicities of Rongalite and the other sulfinates. In contrast, performing the competition experiment at room temperature gave a different product profile in which a good yield of **26** (62%) precipitated from the reaction mixture. No other products were observed in either the precipitate or the extracted filtrate from that reaction. The high selectivity for formation of **26** at room temperature suggests a profile that is more reflective of kinetic control, such that we can argue that **26** forms between 1 and 2 orders of magnitude more rapidly than cyclic products **1a** and **1b**, such that they are not detectable in the ^1^H NMR spectra.

**Table 1:** Results of competition experiments.

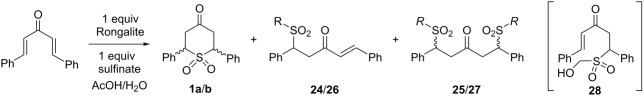				

R (temp., time)	Remaining s.m., %	**1a**/**1b** %	**24**/**26** %	**25**/**27** %

Me (50 °C, 16 h)	15	38	26	20
*p*-Tol (50 °C, 16 h)	0	65	22	12
*p*-Tol (rt, 2 h)	0	<5	>95	0

Further anecdotal evidence that *p*-toluenesulfinate is more reactive than Rongalite is provided by the fact that monoadduct **26** forms readily at room temperature in high yield. This is not the case for the formation of the cyclic Rongalite adducts, which require higher temperatures and a longer reaction time. However, this does not conclusively indicate beyond all doubt the lower nucleophilicity of Rongalite – it is still possible that loss of formaldehyde from an intermediate such as **28** (inset in scheme to Table 1) is rate-determining. We can, however, conclude that conjugate addition of *p*-toluenesulfinate is significantly faster than the slowest step of the double conjugate addition to give **1a** and **1b**. However, the simplest explanation for these results is that Rongalite is significantly less nucleophilic than *p*-toluenesulfinate. This could be related to the inductive withdrawal of electron density by the hydroxy oxygen, which is absent in the other sulfinates.

**Scheme 7:**
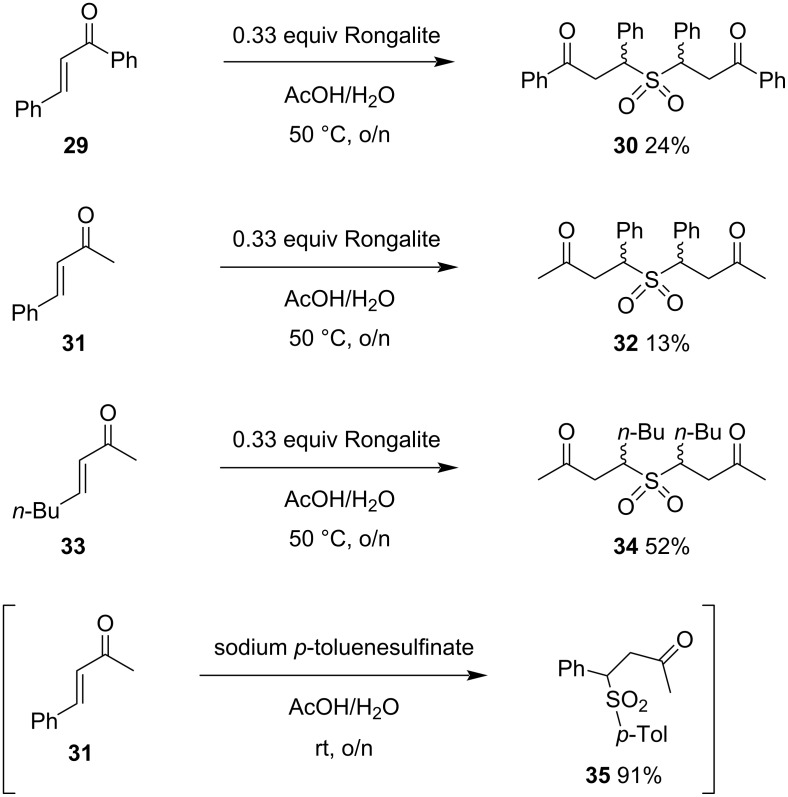
Double intermolecular additions.

We finally elected to examine double intermolecular conjugate addition with some commercially available enones (Scheme 7). These reactions were sluggish, but yielded the expected adducts **30**, **32**, and **34** as 1:1 diastereomeric mixtures (**30** was obtained in a 1:1 mixture according to the crude ^1^H NMR spectrum, but an analytical sample obtained by crystallization consisted of a 3:1 mixture). In contrast, a control reaction of sodium *p*-toluenesulfinate with benzalacetone to give known sulfone **35** [45], occurred readily at room temperature in high yield. It appears that there is scope for further development of Rongalite as a nucleophile in double intermolecular conjugate additions, but full realization of this goal is likely to require the intervention of catalysis. Suitable modes of activation might include well-chosen Lewis acids [46], hydrogen-bond donors [47] or, given the high solubility of Rongalite in water, phase-transfer catalysis [48]. Our earlier results indicate that tolerance of steric effects is greater in reactions with doubly electrophilic substrates that render the second conjugate addition intramolecular, justifying our choice of dienone substrates for this study.

## Conclusion

Methodical investigation of substrate scope allied with detailed computational modelling have improved the understanding of diastereoselective sulfone formation from the reaction of Rongalite and readily available dienones. We have also established that Rongalite, despite apparent lower inherent reactivity than other sulfinates, can be used to prepare sterically encumbered systems and even participate in double intermolecular conjugate additions with β-substituted enones. Our work will be of interest to other workers involved in the synthesis of sulfur heterocycles and also those looking to use Rongalite as a source of nucleophilic sulfur in other transformations. We are currently examining extensions of this method and other sulfinate reaction development and catalysis.

## Supporting Information

File 1 Experimental procedures, copies of ^1^H and ^13^C NMR spectra and further computational details.

## Data Availability

Data generated and analyzed during this study is available from the corresponding author upon reasonable request.
