# Supplementary material for: Rongalite addition to dienones: diastereoselectivity in cyclic sulfone synthesis; stereochemical rationalization and prospects as a general conjugate nucleophile
Source: Beilstein J Org Chem. 2026 May 13;22:742–52. doi: 10.3762/bjoc.22.56 (PMC13181607; doi:10.3762/bjoc.22.56)

BEILSTEIN JOURNAL OF ORGANIC CHEMISTRY

Supporting Information

for

Rongalite addition to dienones: diastereoselectivity in cyclic sulfone synthesis; stereochemical rationalization and prospects as a general conjugate nucleophile

Melina Goga, Hao Zong, James Franco, Jazmine Prana, Rudolph Michel, Antonia Muro, Elana Rubin, Janet Brenya, Henk Eshuis and Magnus W. P. Bebbington

Beilstein J. Org. Chem. **2026,** *22,* 742–752. doi:10.3762/bjoc.22.56

Experimental procedures, copies of ^{1}H and ^{13}C NMR spectra and further computational details

License and Terms: This is a supporting information file under the terms of the Creative Commons Attribution License (https://creativecommons.org/licenses/by/4.0). Please note that the reuse, redistribution and reproduction in particular requires that the author(s) and source are credited and that individual graphics may be subject to special legal provisions.
The license is subject to the *Beilstein Journal of Organic Chemistry* terms and conditions: (https://www.beilstein-journals.org/bjoc/terms)

Computational methods:

To account for structural flexibility, the CREST (conformer-rotamer ensemble sampling tool) utility/driver[1,2] for the xtb program[3] was used to perform conformational sampling for all structures to identify conformers. The conformer with the lowest energies for each molecule was reoptimized at the density functional theory (DFT) level. For CREST, the iMTD-GC algorithm was used with the GFN2-xTB method with the default settings for the metadynamics [1].

All DFT level structure optimizations were performed using the PBE0[4] functional and the def2-SVP basis set.[5] The DFT calculations were performed using the ridft module from the TURBOMOLE program package (v7.7).[6] Resolution-of-the-identity methods were employed to approximate the 4-center integrals in DFT with auxiliary basis sets[7] corresponding to the basis set of choice. Reaction paths were computed using an internal reaction coordinate (IRC) method as implemented in the Woelfling module of TURBOMOLE. The highest energy structure was used as an initial guess for the transition state, which was optimized and verified using vibrational frequencies. For the optimized structures single-point energy calculations were performed with the def2-TZVP basis set,[4] which provides energies well converged for most functionals. The hybrid GGA PBE0 functional[8] with Grimme's dispersion correction D4[9,10] was used. This combination provides good relative energies at reasonable computational cost. All energies presented are electronic energies. It is expected that zero point vibrational energies and entropy effects affect these systems in a very similar way.

Rotational barriers were computed using the ORCA program package[11] at the PBE0/def2-SVP level. The dihedral angle was rotated in steps of 10 degrees and for each step a constrained geometry optimization was performed.

Results from computation:

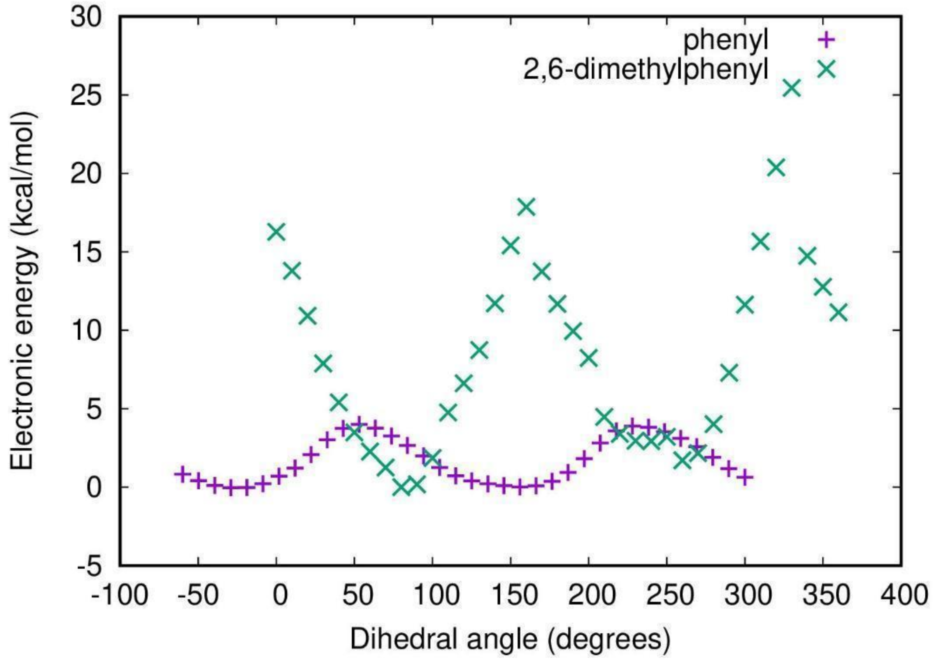

Figure S1: Plot of energy vs dihedral angle for sulfone **10a** (2,6-dimethylphenyl and phenyl substituents). Results were obtained with ORCA using PBE0 and the def2-SVP basis set (see computational methods).

Computer-generated images of relevant structures

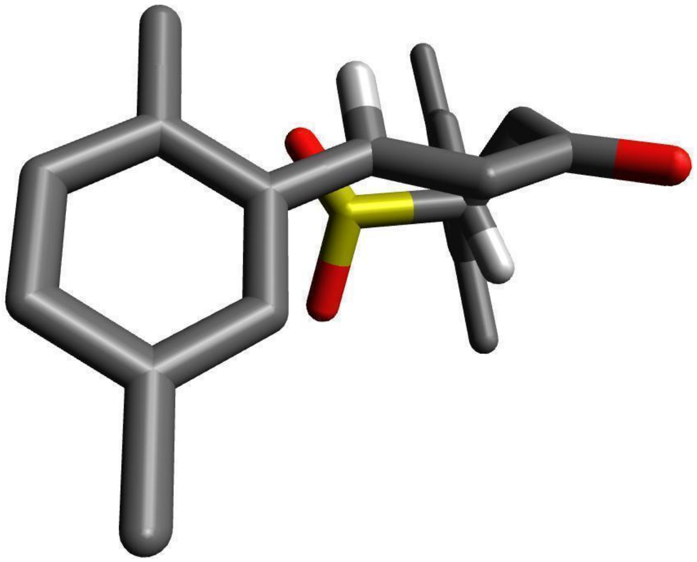

Figure S2: Calculated transition state leading to *trans*-**12a** (Acetic acid molecules are omitted and only H atoms relevant to relative stereochemistry are shown). Structure obtained using TURBOMOLE with PBE0 and def2-SVP basis set (see computational section).

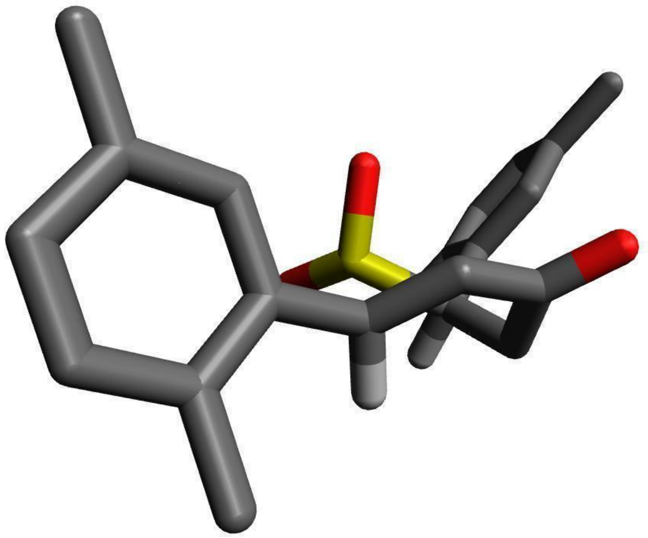

Figure S3: Calculated transition state leading to *cis*-**12b** (acetic acid molecules are omitted). Structure obtained using TURBOMOLE with PBE0 and def2-SVP basis set (see computational section).

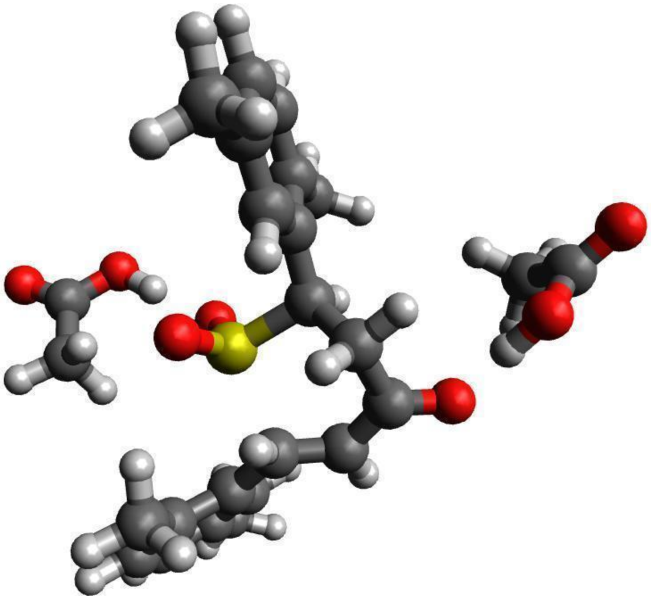

Figure S4: TS-*trans* (formation of **12a**) with acetic acid molecules included. Structure obtained using TURBOMOLE with PBE0 and def2-SVP basis set (see computational section).

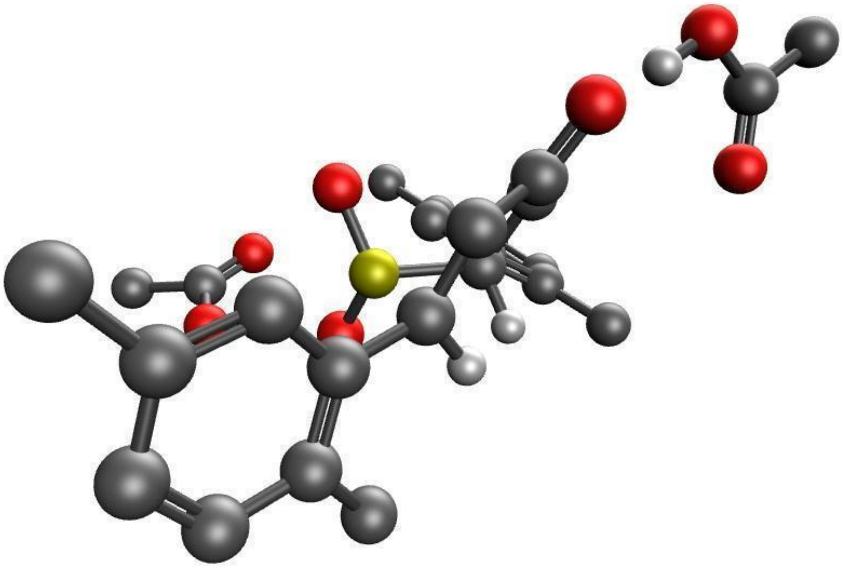

Figure S5: TS-*cis* (formation of **12b**) with acetic acid molecules included. Structure obtained using TURBOMOLE with PBE0 and def2-SVP basis set (see computational section).

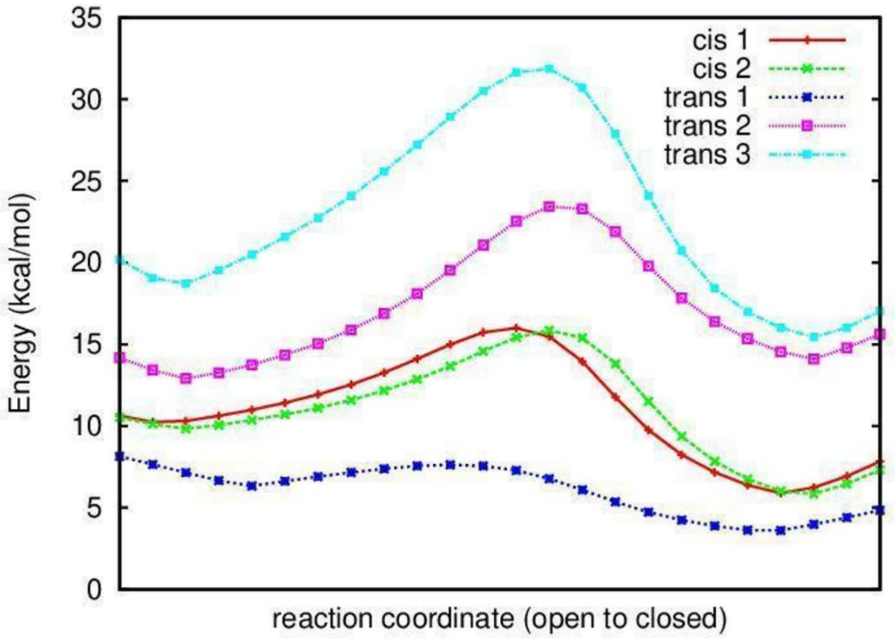

Figure S6: Energy diagram vs reaction coordinate for ring closure for formation of **12a** (*trans*) and **12b** (*cis*). Structure obtained using TURBOMOLE with PBE0 and def2-SVP basis set (see computational section). The Woelfling program was used for the Internal reaction coordinate search.

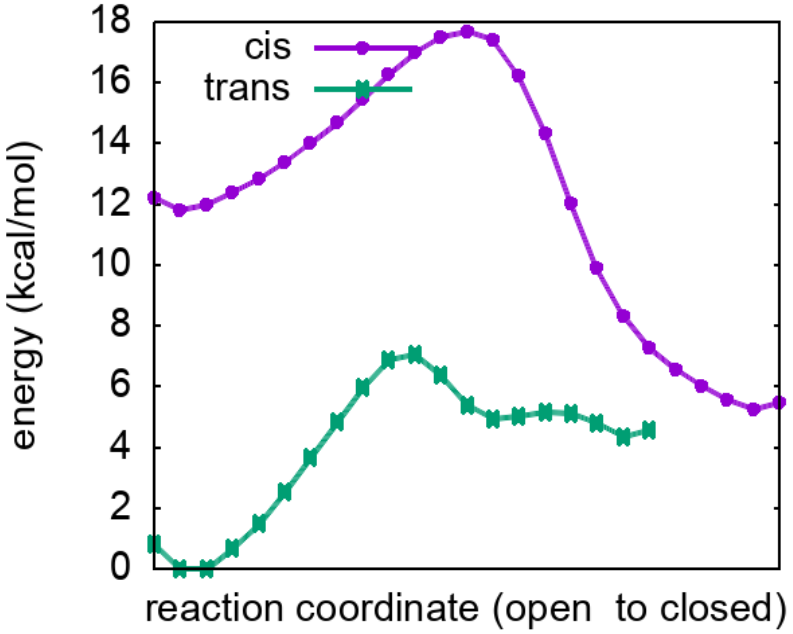

Figure S7: Energy diagram vs reaction coordinate for ring closure for formation of **1a** (*trans*) and **1b** (*cis*). Structure obtained using TURBOMOLE with PBE0 and def2-SVP basis set (see Computational Section). The Woelfling program was used for the Internal Reaction Coordinate search.

Relative energies for the reaction path optimization for **12a** (*trans*) and **12b** (*cis*) obtained using the Woelfling procedure in Turbomole are shown in Figure S6. The reaction involves the ring closure resulting in the enolate intermediate. Two acetic acid solvent molecules were included to model the stabilizing impact of the solvent. See Figures S2–S5 for their orientation. Several converged pathways for each *cis* and *trans* structure are included in Figure S7. Comparing the lowest energy pathways for **12a** and **12b** it is evident that the *cis* configuration shows a much higher barrier to ring closure (6.0 kcal/mol) than the *trans* configuration (1.3 kcal/mol). Constrained CREST calculations on the maximum energy structures for the pathways and subsequent transition state searches for the 26 lowest conformers for each maximum energy structure resulted in an energy difference of 1.8 kcal/mol between the lowest energy *cis* transition state and the lowest energy *trans* transition state, with the *trans* state as the most stable. The lower barrier for the *trans* pathway and the lower energy for the *trans* transition state both support the preferential formation of the *trans* product **12a**. Given the small relative energy difference of the **12a** and **12b** transition states and the much more pronounced difference in barrier height we conclude that kinetic factors are most likely playing a decisive role in product selectivity, thereby favoring the *trans* product over the *cis* product. This is in agreement with a simple Arrhenius analysis. Using the barrier height difference of ≈5 kcal/mol for **12a** and **12b** and assuming an identical intermediate structure for both pathways, a product ratio of 1:5000 would be expected, which is in agreement with the absence of the minor product in the crude NMR spectrum.

For **1a** and **1b** the barrier heights are found to be 7.0 kcal/mol and 6.0 kcal/mol, respectively, both relatively high and quite similar to each other (see Figure S7). Here, the minor product (*cis*) has the lower barrier, but this may be due to the difficulty of defining the equilibrium geometry of the reactant. The open-ring structure is very flexible, and it is challenging to define the reaction starting point consistently for the *cis* and *trans* pathways. This is likely to result in an uncertainty in the barrier height of 1–2 kcal/mol. In addition, the computational methods used have an inherent uncertainty of a similar size. Furthermore, finite temperature effects are not taken into account, i.e. the barrier heights correspond to electronic energy differences, not enthalpy differences (including zero-point energies) or free energy differences. This makes the current study more qualitative than quantitative in nature.

In view of the large conformational flexibility of the reactants a variety of reaction pathways exist within a small energy range. This is supported by the large number of conformers found for the transition states. To obtain more quantitative results for barrier heights that can be translated in relative ratios of products it is likely necessary to treat an ensemble of reaction pathways. This is beyond the scope of this study; in view of this and of the inherent uncertainty in the used methods we limit ourselves to a qualitative analysis.

XYZ coordinates of optimized transition states of 1a, 1b, 12a, 12b including two solvent molecules

1a

52

Energy =

C	-1.5104692	1.7889518	0.9513947
C	-0.1022558	1.7720418	1.0683267
C	0.7721451	2.8250404	0.5080157
C	0.3024839	3.8052012	-0.3737233
C	1.1690211	4.7647069	-0.8901342
C	2.5197918	4.7525503	-0.5458876
C	3.0016963	3.7702048	0.3188896
C	2.1353208	2.8174463	0.8422355
S	0.6932217	-0.1049658	0.2066870
O	1.0677337	-0.3576990	-1.2253306
O	1.6814168	-0.5529858	1.2307252
C	-0.8478858	-1.1175251	0.4788520
C	-0.4170080	-2.5433111	0.4819706
C	0.0985089	-3.1477061	1.6349132
C	0.5665586	-4.4581728	1.5968304
C	0.5379920	-5.1811375	0.4055469
C	0.0388208	-4.5830404	-0.7507341
C	-0.4315382	-3.2740485	-0.7124363
C	-1.5707979	-0.5926610	1.7219467
C	-2.2953089	0.7025326	1.3801064
O	-3.5506697	0.6672294	1.3664088
H	-2.0211825	2.6056864	0.4379207
H	0.3249727	1.3340302	1.9798021
H	-0.7505306	3.8164373	-0.6598594
H	3.1986744	5.5030809	-0.9588081
H	4.0619762	3.7407894	0.5810972
H	-1.4486010	-0.9067230	-0.4188631
H	0.9678621	-4.9151339	2.5049920
H	0.9108479	-6.2079780	0.3764518

H	-0.8060174	-2.7981805	-1.6219889
H	-2.3239965	-1.3225008	2.0511909
H	-0.8524593	-0.4545138	2.5471379
H	0.0207187	-5.1392230	-1.6914792
H	0.7846685	5.5258154	-1.5737487
H	-3.4214092	1.1827257	-1.6580449
O	-5.6776974	3.1809791	-1.6345536
C	-4.5107656	1.0977082	-1.5125957
H	-4.6701672	0.2697229	-0.8078316
C	-5.0427390	2.4056982	-0.9590655
H	-4.9849964	0.8939057	-2.4800727
O	-4.7429085	2.6583712	0.3016679
H	-4.2669255	1.8765330	0.7503164
H	2.9964804	-2.1209368	0.1675196
O	5.3967386	-1.8503622	-1.7757457
C	3.3633513	-2.5985363	-0.7545645
H	2.4663145	-2.8618705	-1.3346091
C	4.2351243	-1.6373267	-1.5250718
H	3.9371657	-3.5019658	-0.5180857
O	3.6463610	-0.5141028	-1.9201043
H	2.6978926	-0.4699000	-1.6427715
H	0.1492240	-2.5787767	2.5644283
H	2.5134988	2.0268653	1.4960452

1b

52

Energy =

C	-0.9164381	1.2186280	1.6140866
C	0.2876943	0.4914260	1.6430941
C	1.6127663	1.0209435	1.2317536
C	2.7276323	0.1707038	1.1672381
C	3.9810188	0.6756926	0.8388334
C	4.1495959	2.0336336	0.5680521

C	3.0510626	2.8891619	0.6428760
C	1.7961881	2.3883075	0.9794630
S	0.0583849	-1.0720629	-0.0989055
O	1.2601295	-1.1277472	-1.0227170
O	-0.6076810	-2.3754732	0.1553595
C	-1.1837013	-0.0552251	-1.0718072
C	-1.1748439	-0.5980836	-2.4571732
C	-1.9040093	-1.7599112	-2.7429146
C	-1.8664908	-2.3285845	-4.0119871
C	-1.0967646	-1.7475020	-5.0190977
C	-0.3555439	-0.6008835	-4.7398593
C	-0.3866792	-0.0361612	-3.4677175
C	-0.8746322	1.4315529	-0.8896289
C	-1.4222705	1.8665836	0.4625989
O	-2.4293617	2.6135798	0.4458245
H	-1.6227047	1.1154046	2.4448769
H	0.3656185	-0.2480907	2.4507015
H	2.6133840	-0.9026943	1.3452002
H	5.1365927	2.4257035	0.3069219
H	3.1741765	3.9588287	0.4519386
H	-2.1329300	-0.3073568	-0.5768798
H	-2.4415026	-3.2358902	-4.2159874
H	-1.0685427	-2.1923121	-6.0173418
H	0.2158726	0.8481708	-3.2527650
H	0.2098661	1.6015894	-0.9754332
H	-1.3781721	2.0248227	-1.6666084
H	0.2640683	-0.1466667	-5.5177799
H	4.8319776	-0.0084401	0.7846739
H	-3.9577621	4.4520751	4.9149966
O	-3.5497569	1.7001407	3.5874345
C	-4.5898401	3.6321272	4.5408312
H	-5.4957626	4.0909706	4.1181116
C	-3.8445387	2.8706054	3.4648415

H -4.8524313 2.9586101 5.3650571

O -3.5685209 3.6255315 2.4338499

H -3.0707843 3.1171881 1.6733544

H 3.7222174 -5.4493479 1.1558785

O 2.8169096 -3.0719849 1.1440817

C 3.2350692 -5.2277576 0.1992255

H 2.3460214 -5.8667293 0.0839672

C 2.8026478 -3.7812735 0.1604069

H 3.9073587 -5.4501115 -0.6418440

O 2.4149161 -3.4188778 -1.0376104

H 1.9975681 -2.4930714 -1.0223274

H -2.4862903 -2.2263626 -1.9453808

H 0.9412442 3.0615777 1.0759303

12a

64

Energy =

C 1.4616123 1.9954081 0.8999575

C 1.7940575 0.6341206 1.0573853

C 3.0712140 0.0697993 0.5681944

C 3.6642881 0.5950084 -0.5856503

C 4.8577497 0.0928545 -1.1101669

C 5.4440617 0.6654161 -2.3696903

C 5.4616205 -0.9739645 -0.4402886

C 4.8755423 -1.5207094 0.6998926

C 3.6778705 -1.0279622 1.2223918

C 3.0621182 -1.6645642 2.4369808

S 0.2078709 -0.6056083 0.0057636

O 0.2421389 -0.9135557 -1.4690724

O -0.1194433 -1.7495584 0.9090227

C -1.1730695 0.6487201 0.2030691

C -2.4791327 -0.0783946 0.1231768

C -3.1976082 -0.1453524 -1.0919864

C	-2.7009716	0.5168869	-2.3468868
C	-4.4010983	-0.8527353	-1.1017304
C	-4.8883906	-1.4932315	0.0362252
C	-4.1761755	-1.4506702	1.2349217
C	-4.6612383	-2.1578089	2.4686633
C	-2.9733638	-0.7379240	1.2517169
C	-0.9228474	1.4757970	1.4745921
C	0.1821654	2.4966873	1.2203521
O	-0.1147521	3.7137358	1.1686709
H	2.1601452	2.6864005	0.4231267
H	1.3933547	0.1166001	1.9346864
H	3.1565168	1.4141040	-1.1016442
H	5.4641893	1.7659704	-2.3415480
H	6.4719157	0.3101667	-2.5339684
H	4.8473184	0.3763565	-3.2507451
H	6.3972974	-1.3937366	-0.8216488
H	5.3606956	-2.3647209	1.1989379
H	3.0155041	-0.9623146	3.2859177
H	2.0329885	-1.9981629	2.2286354
H	3.6483384	-2.5374384	2.7577738
H	-1.0360836	1.2953446	-0.6737999
H	-3.3665043	0.2946486	-3.1928844
H	-2.6579393	1.6144486	-2.2387581
H	-1.6889010	0.1681705	-2.6018599
H	-4.9722777	-0.9068832	-2.0331126
H	-5.8345810	-2.0404154	-0.0138056
H	-3.9694622	-2.9652667	2.7598149
H	-5.6532715	-2.6062451	2.3123849
H	-4.7327621	-1.4693785	3.3260571
H	-2.3919083	-0.7065882	2.1757544
H	-1.8438375	2.0106227	1.7489449
H	-0.6676129	0.8133926	2.3189260
H	2.3350290	-2.9727192	-1.3977308

O	0.9141628	-5.1985582	-3.0243044
C	1.6730394	-3.8321995	-1.2082501
H	1.0172605	-3.5398074	-0.3731602
C	0.8557232	-4.1434099	-2.4411152
H	2.2717449	-4.7099788	-0.9386572
O	0.0559104	-3.1708999	-2.8578945
H	0.1158054	-2.3657359	-2.2834818
H	-2.9455781	5.6757019	-1.1971515
O	-4.1306791	5.7276494	1.0521425
C	-2.4691376	4.9798540	-0.4961238
H	-2.6596673	3.9498316	-0.8393189
C	-3.0790578	5.1594859	0.8806263
H	-1.3785046	5.1137770	-0.4799910
O	-2.4009270	4.6243513	1.8807985
H	-1.5021682	4.2544981	1.5879231

12b

64

Energy =

C	1.2612844	2.2623011	-0.8727421
C	0.3256819	2.2703286	0.1781814
C	-0.7084797	3.3184118	0.3079779
C	-1.0824936	4.0857387	-0.8015615
C	-2.0560388	5.0851257	-0.7308262
C	-2.4380071	5.8697065	-1.9547391
C	-2.6707538	5.3079852	0.5030261
C	-2.3289250	4.5384526	1.6138178
C	-1.3621028	3.5340670	1.5458037
C	-1.0626759	2.6916925	2.7531463
S	-0.7462918	0.2458414	-0.0581665
O	-1.7126808	-0.2742103	0.9926832
O	-1.0607674	-0.1002249	-1.4705218

C	0.8618169	-0.5747839	0.3642913
C	0.7543916	-2.0656144	0.5825997
C	1.6421200	-2.7018131	1.4804276
C	2.6932214	-1.9443799	2.2450771
C	1.5369924	-4.0864493	1.6359151
C	0.5943528	-4.8350888	0.9346589
C	-0.2815071	-4.2164385	0.0416199
C	-1.3174923	-4.9893835	-0.7230268
C	-0.1786089	-2.8301871	-0.1226951
C	1.9216627	-0.2266169	-0.6855574
C	2.1444531	1.2008378	-1.1757899
O	3.0773166	1.3455128	-2.0035817
H	1.2871257	3.0914085	-1.5832494
H	0.6354392	1.8511412	1.1379147
H	-0.6020264	3.8788279	-1.7607400
H	-1.5518208	6.2814548	-2.4630704
H	-2.9611031	5.2330919	-2.6875656
H	-3.1054694	6.7076309	-1.7048158
H	-3.4371004	6.0836841	0.5959029
H	-2.8371945	4.7166126	2.5661637
H	-0.0130412	2.7888693	3.0769777
H	-1.2513495	1.6268265	2.5376042
H	-1.6999226	2.9847531	3.5997205
H	1.1124616	-0.1032575	1.3280369
H	2.2662337	-1.1029058	2.8153738
H	3.1975437	-2.6056212	2.9647833
H	3.4682250	-1.5273115	1.5803380
H	2.2162289	-4.5906830	2.3307339
H	0.5366347	-5.9172353	1.0893011
H	-1.3739101	-6.0341138	-0.3810919
H	-1.0850814	-5.0020556	-1.8013367
H	-2.3060445	-4.5188855	-0.6101476
H	-0.8636954	-2.3262840	-0.8082802

H	2.9022852	-0.6116136	-0.3615812
H	1.6635477	-0.7813797	-1.6049519
H	7.3936807	-1.0991398	-2.4634403
O	5.1400968	-0.7781295	-0.3279651
C	6.6665895	-1.7157857	-1.9143134
H	6.3235505	-2.5006589	-2.6052634
C	5.4873124	-0.8678511	-1.4899226
H	7.1395609	-2.1711669	-1.0363144
O	4.9129750	-0.2791018	-2.5042179
H	4.1191367	0.3446522	-2.2311038
H	-6.3704365	-1.2186380	-0.9166672
O	-3.8172176	-2.8023492	-0.2154407
C	-6.0636725	-1.9732468	-0.1766562
H	-6.3360067	-2.9749061	-0.5291430
C	-4.5688702	-1.8894378	0.0387933
H	-6.5927740	-1.7403736	0.7593477
O	-4.2170596	-0.7200652	0.5221186
H	-3.2174552	-0.6379181	0.6485550

Synthesis (copies of NMR spectra are included in this file):

Melting points were obtained in open capillary tubes and are uncorrected. ^{1}H NMR spectra were recorded on a Bruker Spectrospin 400 spectrometer at 400 MHz and referenced to residual solvent. ^{13}C NMR spectra were recorded using the same spectrometer at 100 MHz respectively. Chemical shifts (δ in ppm) were referenced to tetramethylsilane (TMS) or to residual solvent peaks ($CDCl_3$ at δH 7.26). *J* values are given in Hz and s, d, dd, ddd, t, dt, q, m, br and app. abbreviations correspond to singlet, doublet, doublet of doublet, doublet of doublet of doublet, triplet, triplet of doublet, quartet, multiplet, broad and apparent respectively. High-resolution mass spectra were obtained using a Q Exactive™ Plus Hybrid Quadrupole-Orbitrap™ mass spectrometer (Montclair State) or an LTQ Orbitrap (Purdue Univ.). Infrared spectra were obtained on a Perkin-Elmer Spectrum Two FT-IR Universal ATR Sampling Accessory, deposited neat or as a chloroform solution to a diamond/ZnSe plate. Flash column chromatography was carried out using Matrix silica gel 60 from Fisher Chemicals and TLC was performed using Merck silica gel 60 F254 precoated sheets and visualized by UV (254 nm) and stained by the use of aqueous acidic $KMnO_4$. Eluting solvents are indicated in the text. The apparatus for inert atmosphere experiments was flame-dried under a stream of dry argon.

(In a manner analogous to ref. 12) General procedure double conjugate addition of Rongalite to give cyclic sulfones: Glacial acetic acid (4.7 mL/mmol substrate) was added to dienone substrate (1–2 mmol) in an open round-bottomed flask, followed by water (0.8 mL/mmol Rongalite) and Rongalite (1.5–3 equiv) The mixture was stirred (350 rpm) at 80 °C for 3 h. Equal volumes of ethyl acetate (25 mL/mmol substrate) and water (25 mL/mmol substrate) were added and the mixture extracted twice more with ethyl acetate (25 mL/mmol). The combined organic extracts were washed with saturated aq. $NaHCO_3$ (25 mL/mmol substrate) and water (25 mL/mmol substrate). The organic layer was separated, dried (Na_2SO_4) and the solvent removed at reduced pressure. The product diastereomers were separated by column chromatography on silica gel (EtOAc/hexanes). The major *trans*-isomer invariably eluted with a higher R_f.

Sulfones 1a/1b[13,14]

***trans*-2,6-Diphenyltetrahydro-4*H*-thiopyran-4-one-1,1-dioxide (1a)**.

R_f (50% EtOAc/hexanes) 0.45. White solid, m.p. 176-177 °C. (EtOAc/cyclohexane) (lit. 179-180 °C (Et_2O)) **^{1}H-NMR (400 MHz, $CDCl_3$):** δ 7.49-7.31 (m, 10H), 4.53 (dd, *J* = 8.3, 5.0 Hz, 2H), 3.52 (roofed dd, *J* = 16.3, 8.3 Hz, 2H), 3.38 (roofed dd, *J* = 16.3, 5.0 Hz, 2H). **^{13}C-NMR** ^{13}C NMR (101 MHz, **$CDCl_3$**) δ 203.6, 130.2, 129.6, 129.5, 129.1, 61.3, 44.7. **IR (cm^{-1});** 3032, 2925, 2857, 1727, 1495, 1455, 1326, 1235, 1127. Other data in accordance with the literature.

***cis*-2,6-Diphenyltetrahydro-4*H*-thiopyran-4-one-1,1-dioxide (1b)**.

R_f (50% EtOAc/hexanes) 0.23 White solid, m.p. 235-236 °C (EtOAc/cyclohexane) (lit. 237-8 °C (Et_2O)) **^{1}H-NMR (400 MHz, $CDCl_3$):** δ = ^{1}H NMR (400 MHz, $CDCl_3$), δ 7.48-7.32 (m, 10H), 4.56 (dd, *J* = 14.1, 3.3 Hz, 2H), 3.74 (apparent t, *J* = 14.1 Hz, 2H), 2.95 (dt (triplet poorly resolved), *J* = 14.1, 3.3 Hz, 2H). **^{13}C-NMR (100 MHz, $CDCl_3$):** δ = 202.1, 129.8, 129.6, 129.0, 128.5, 64.4, 45.8. **IR (cm^{-1});** 3071, 3033, 2962, 2927, 1729, 1495, 1456, 1327, 1239, 1131. Other data in accordance with the literature, in particular the coupling constant analysis in ref 15.

1a from sulfide oxidation:
trans-Sulfide **2** was prepared according to the method of Lanfranchi,[14] (0.62 g, 2.3 mmol) and was dissolved in acetic acid (15 mL) and then H_2O_2 (30% in water, 1.36 mL, 12 mmol) was added. The mixture was heated to 60 °C for 16 h, then cooled to room temperature. Volatiles were removed at reduced pressure and the residue purified by chromatography (25–50% EtOAc/hexanes). Removal of the solvent gave *trans*-sulfone **1a** (0.45 g, 69%) with identical spectroscopic data to the major product from Rongalite addition.

***rac*-(2α,4α,6β)-2,6-Diphenyltetrahydro-4*H*-thiopyran-4-(*N*-phenyl)amine-1,1-dioxide (3) (dl)**.
Aniline (0.67 mmol, 62.4 mg, 61 µl) and *trans*-**1a** (200 mg, 0.67 mmol) were mixed in 1,2-dichloroethane (2 mL) and cooled to 0 °C. Acetic acid (60 mg, 1.0 mmol) was added and reaction stirred for 5 min. $NaBH(OAc)_3$ (1.33 mmol, 282 mg) was then added as one portion, reaction warmed to rt and stirred for 24 h. Saturated $NaHCO_3$ (1 mL) was added dropwise, then the mixture extracted with DCM (3 × 5 mL), and dried ($MgSO_4$). Removal of solvent gave 220 mg crude yellow oil. Chromatography (10% EtOAc/hexanes to 30% EtOAc/hexanes) gave fraction 1, 115 mg (46%) of a white solid identified as **3**. Further elution with EtOAc gave a second fraction, 54 mg (26%) of a white solid identified as alcohol **4** (see below for characterization data). For **3**: R_f (10% EtOAc/hexanes) 0.15. White solid, m.p. 182-183 °C (EtOAc/hexanes) **^{1}H NMR (400 MHz, $CDCl_3$)** δ 7.73 (d, *J* = 7.5 Hz, 2H), 7.63 – 7.34 (m, 8H), 7.30 – 7.19 (m, 2H), 6.83 (t, *J* = 7.5 Hz, 1H), 6.69 (d, *J* = 7.5 Hz, 2H), 4.62 (apparent t, *J* = 5.3 Hz, 1H), 4.44-4.27 (m, 2H), 3.95-3.15 (bs, 1H, NH), 2.98 (roofed d, *J* = 14.9 Hz, 1H), 2.86 – 2.59 (m, 3H). **^{13}C NMR (101 MHz, $CDCl_3$)** δ 145.8, 132.6, 129.9, 129.7, 129.6, 129.3, 129.3, 129.1, 128.8, 128.8, 118.6, 113.7, 64.5, 62.0, 47.4, 36.2, 34.8. **IR (cm^{-1});** 3400, 3061, 3033, 2955, 2939, 2893, 1601, 1447, 1293, 1269, 1120. HRMS: (M + H^+) Calcd for $C_{23}H_{24}O_2NS$ = 378.152776; found 378.15195 (δ = -2.2 ppm).

***rac*-(2α,4α,6β)-2,6-Diphenyltetrahydro-4*H*-thiopyran-4-ol-1,1-dioxide (4) (dl)**.
$NaBH_4$ (27 mg, 0.67 mmol) was added to a solution of *trans*-**1a** (200 mg, 0.67 mmol) in MeOH (5 mL) at rt. The reaction was stirred for 1 h at rt. Water (1 mL) was added and the mixture extracted with EtOAc and dried ($MgSO_4$). Filtration of the drying agent and removal of the solvent gave 180 mg solid which was the expected single diastereomer by ^{1}H NMR. Recrystallization (EtOAc/hexanes) gave 164 mg (82%) suitable for characterization, yield 82%. R_f (50% EtOAc/hexanes) 0.27. White solid, m.p. 177 °C dec. (EtOAc/hexanes) **^{1}H NMR (400 MHz, DMSO)** δ 7.65 – 7.35 (m, 10H), 5.30 (d, *J* = 4.6 Hz, 1H), 4.79 (dd, *J* = 6.8, 4.6 Hz, 1H), 4.63 (dd, *J* = 12.5, 3.2 Hz, 1H), 4.53 – 4.33 (m, 1H), 2.74 – 2.63 (m, 1H), 2.60 – 2.46 (m, 1H, superposed on DMSO), 2.44-2.33 (m, 2H). **^{13}C NMR (101 MHz, DMSO)** δ 133.8, 131.8, 130.5, 130.2, 129.0, 129.0, 128.7, 128.7, 63.6, 63.6, 62.1, 37.6, 37.4. **IR (cm^{-1})**; 3519, 3061, 3031, 2932, 1495, 1453, 1287, 1114. **HRMS:** (M + H^+) calcd for $C_{17}H_{19}O_3S$, 303.105492; found 303.10533 (δ = -0.5 ppm).

(2α,4α,6α)-2,6-Diphenyltetrahydro-4*H*-thiopyran-4-ol-1,1-dioxide (5) (*meso*).

$NaBH_4$ (70 mg, 1.84 mmol) was added to a solution of *cis*-**1b** (400 mg, 1.36 mmol) in MeOH (20 mL) at rt. The reaction was stirred 90 min at rt. 1 mL Water was added and the reaction stirred for 10 min. MeOH was then removed at reduced pressure. The mixture was partitioned between EtOAc and water (10 mL each). The organic layer was separated and the aqueous layer extracted with EtOAc (2 × 10 mL) The combined organic layers were dried (Na_2SO_4) and the solvent removed at reduced pressure, giving 380 mg solid. ^{1}H NMR suggested a ≈9:1 mixture of diastereomers **5** and **6** (**6** was not isolated). Column chromatography (SiO_2, 30% to 50/50 EtOAc/hexanes) gave a single isomer that was recrystallized from acetone to give 246 mg of the title compound. R_f (50% EtOAc/hexanes) 0.25. White solid, m.p. 241 °C dec. (EtOAc/hexanes) : **^{1}H NMR (400 MHz, DMSO**) δ 7.63-7.15 (m, 10H), 5.29 (s, 1H), 4.70 (d, *J* = 13.2 Hz, 2H), 4.14 (s, 1H), 2.43 (d, *J* = 13.2 Hz, 2H 0o(partially superposed on DMSO), 2.26 (d, *J* = 13.2 Hz, 2H).0**^{13}C NMR (101 MHz, DMSO)** δ 131.5, 130.4, 129.2, 128.9, 66.6, 63.4, 39.7. **IR (cm^{-1});** 3545, 3065, 3040, 3033, 2927, 1495, 1455, 1276, 1130, 1060. **HRMS:** (M + H^+) calcd for $C_{17}H_{19}O_3S$, 303.105492; 303.10551 (δ = +0.05 ppm).

1,5-Bis(2,6-dimethylphenyl)penta-1,4-dien-3-one (7)

To a solution of 2,6-dimethylbenzaldehyde, 7.45 mmol, 1.00 g, weighed liquid) and acetone (3.80 mmol, 221 mg, 0.28 mL) in absolute EtOH (7.45 mL, 1.0 M in aldehyde) was added NaOH (7.45 mmol) 0.298 g in water (5.5 mL) at room temperature. The reaction was stirred overnight and the resulting precipitate (1.20 g) was filtered off. Column chromatography (5% EtOAc/hexanes) gave a white solid (0.80 g, 73%). R_f (5% EtOAc/hexanes) 0.25. White solid, m.p. 145-146 °C.

^{1}H-NMR (400 MHz, $CDCl_3$): (EtOAc/Hexanes) ^{1}H NMR (400 MHz, $CDCl_3$) δ 7.90 (d, *J* = 16.4 Hz, 2H), 7.22 – 7.03 (m, 6H), 6.73 (d, *J* = 16.4 Hz, 2H), 2.42 (s, 12H). **^{13}C-NMR** ^{13}C NMR (101 MHz, $CDCl_3$) δ 189.0, 142.1, 136.9, 134.4, 1312, 128.5, 128.4, 21.2. **IR (cm^{-1}):** 3056, 2961, 2860, 1648, 1589, 1310, 1188. **HRMS** (M+H^+), calcd for $C_{21}H_{23}O$ = 291.174890, Found 291.17482 (δ = -0.2 ppm)

1-(2,6-Dimethylphenyl)-5-phenylpenta-1,4-dien-3-one (9)

Solid NaOH (596 mg, 14.9 mmol) was added to solution of 2,6-dimethylbenzaldehyde (2.00 g weighed liquid, 14.9 mmol) and 4-phenylbut-3-en-2-one (benzalacetone, 14.9 mmol, 2.18 g) in EtOH (20 mL) at rt. The resulting thick precipitate was filtered off after 1 h. The mixture was filtered and washed with water (3 × 20 mL) to give a yellow solid. 2.03 g, 52%. R_f (5% EtOAc/hexanes) 0.21, m.p. 88-90 °C (EtOAc/Hexanes)

^{1}H-NMR (400 MHz, $CDCl_3$): δ 7.90 (d, *J* = 16.2 Hz, 1H), 7.74 (d, *J* = 16.0, 1H), 7.64-7.58 (m, 2H), 7.44-7.38 (m, 3H), 7.20 – 7.02 (m, 4H), 6.73 (d, *J* = 16.2, Hz, 1H), 2.41 (s, 6H). **^{13}C-NMR (101 MHz, $CDCl_3$)** δ 188.9, 143.5, 142.0, 136.9, 134.8, 134.5, 131.1, 130.6, 129.0, 128.4, 128.3, 125.6, 21.2. **IR (cm^{-1}):** 3061, 3021, 2970, 2954, 1667, 1619, 1590, 1334, 1106. **HRMS** (M+H^+), calcd for $C_{19}H_{19}O$ = 264.14359, Found 264.1454 (δ = + 6.9 ppm)

***trans*-2-(2,6-Dimethylphenyl)-6-phenyltetrahydro-4*H*-thiopyran-4-one-1,1-dioxide (10a**)

General procedure with 500 mg (1.9 mmol) **9**, Rongalite (1.75 equiv, 3.34 mmol, 513 mg), acetic acid (8.9 mL) and water (1.5 ml) gave a crude oil (458 mg). Chromatography gave 150 mg starting material and 240 mg of *trans*-**10a** (38%, 55% by recovered starting material). Impure traces of

the minor isomer could not be purified. R_f (30% EtOAc/hexanes) 0.31. White solid, m.p. 166-168 ^{o}C (EtOAc/hexanes); Restricted rotation was revealed by the NMR data: **^{1}H NMR (400 MHz, $CDCl_3$)** δ 7.55-7.49 (m, 2H), 7.48-7.40 (m, 3H), 7.12 – 7.00 (m, 2H), 6.92 (d, *J* = 7.3 Hz, 1H), 5.06 (dd, *J* = 14.3, 2.9 Hz, 1H), 4.62 (dd, *J* = 6.1, 4.0 Hz, 1H), 4.21 (dd, *J* = 15.8, 14.3 Hz, 1H), 3.71 (dd, *J* = 16.4, 6.1 Hz, 1H), 3.37 (ddd, *J* = 16.4, 4.0, 2.9 Hz, 1H), 2.98 (dd, *J* = 15.8, 4.0 Hz, 1H), 2.68 (s, 3H), 1.71 (s, 3H). **^{13}C-NMR (101 MHz, $CDCl_3$)** δ 203.9, 139.7, 139.3, 132.4, 131.2, 129.5, 129.4, 129.1, 129.0, 129.0, 125.7, 64.5, 54.8, 45.3, 43.6, 22.3, 20.4. **IR cm^{-1}**: 3065, 3032, 2921, 2855, 1720, 1316, 1219, 1127. **HRMS** (M+H$^+$), calcd for $C_{19}H_{21}SO_3$ = 329.12114, Found 329.12100 (δ = -0.42 ppm).

***trans*-2,6-Bis(2,5-dimethylphenyl)tetrahydro-4*H*-thiopyran-4-one-1,1-dioxide (12a)**

General procedure with 500 mg (1.72 mmol) **11**,[8] Rongalite (1.75 equiv, 3.44 mmol, 530 mg) acetic acid (8.0 mL) and water (1.35 mL) gave a crude oil (458 mg). Chromatography (20% EtOAc/hexanes) gave 364 mg of *trans*-**12a** (59%). R_f (20% EtOAc/hexanes) 0.25. White solid, m.p. 191-192 ^{o}C (EtOAc/hexanes); **^{1}H NMR** (400 MHz, $CDCl_3$) δ 7.17 (s, 2H), 7.10 (d, *J* = 1.2 Hz, 4H), 5.04 (dd, *J* = 8.5, 5.1 Hz, 2H), 3.50 (ddd, *J* = 16.2, 8.5, 1.4 Hz, 2H), 3.32 (ddd, *J* = 16.2, 5.1, 1.4 Hz, 2H), 2.35 (s, 6H), 2.20 (s, 6H). **^{13}C NMR** (101 MHz, $CDCl_3$) δ 204.2, 136.2, 135.8, 131.2, 130.1, 129.1, 128.0, 56.7, 45.3, 21.1, 19.4. **IR cm^{-1}**: 3023, 2955, 2922, 2868, 1715, 1503, 1315, 1118. **HRMS** (M+H$^+$), calcd for $C_{21}H_{25}SO_3$ = 357.152442, Found 357.15103. (δ = - 4.0 ppm)

5-(Methanesulfonyl)-1,5-diphenylpent-1-en-3-one (24) (R = Me)

Sodium methanesulfinate (214 mg, 0.5 equiv, 2.1 mmol) in water (2.5 mL) was added to a solution of dibenzalacetone (1.03 g, 4.2 mmol) in AcOH (19.5 mL). The mixture was stirred at 50 °C for 3 h and then overnight at room temperature. The mixture was partitioned between EtOAc (150 mL) and water (50 mL). The organic layer was separated, washed with saturated $NaHCO_3$ (50 mL) and water (50 mL) and dried (Na_2SO_4). Removal of solvent gave 1.02 g crude solid. Chromatography (10% EtOAc/hexanes to neat EtOAc) gave starting material and then 362 mg (55%) of the title compound. White solid, **m.p.** 138-139 ^{o}C, R_f (50% EtOAc/hexanes) 0.40 **^{1}H NMR** (400 MHz, $CDCl_3$) δ 7.64 (d, *J* = 16.2 Hz, 1H), 7.61 – 7.52 (m, 4H), 7.49 – 7.39 (m, 6H), 6.76 (dd, *J* = 16.2, 1.1 Hz, 1H), 4.94 (dd, *J* = 9.4, 3.7 Hz, 1H), 3.85 (ddd, *J* = 17.7, 3.8, 1.1 Hz, 1H), 3.60 (ddd, *J* = 17.7, 9.4, 1.1 Hz, 1H), 2.74 (s, 3H). **^{13}C NMR** (101 MHz, $CDCl_3$) δ 194.6, 144.1, 134.0, 133.3, 131.0, 129.5, 129.3, 129.2, 129.0, 128.5, 125.5, 64.9, 38.8, 38.4. **IR cm^{-1}**: 3053, 3005, 2920, 2849, 1685, 1651, 1610, 1298, 1132. **HRMS** (M+H$^+$), calcd for $C_{18}H_{19}SO_3$ = 315.105492, Found 315.105470. (δ = - 0.1 ppm).

1,5-Bis(methanesulfonyl)-1,5-diphenyl-pentan-3-one (25) (R = Me)

Sodium methanesulfinate (0.65 g, 3.0 equiv, 6.4 mmol) in water (1.5 mL) was added to a solution of dibenzalacetone (0.50 g, 2.13 mmol) in AcOH (9.5 mL). The mixture was stirred at 50 °C for 2 h and then cooled to room temperature. The resulting solid was filtered off and washed with water (2 × 50 mL) to give 612 mg (73%) of an analytical sample. White solid, m.p. 167 ^{o}C dec. Approx 3:2 mixture of diastereomers. Relative stereochemistry of major isomer not ascertained. **^{1}H NMR (400 MHz, DMSO)** δ 7.51 – 7.28 (m, 10H) superposed, 4.87-4.78 (m, 2H) superposed, 3.66 – 3.43 (m, 4H) superposed, 2.83 (s, 3H, minor), 2.80 (s, 3H, major). **^{13}C NMR (101 MHz,**

DMSO) δ Major: 202.5, 133.3, 130.2, 129.0, 128.8, 63.5, 40.4, 38.4. Minor: 202.7, 133.1, 130.1, 129.0, 128.8, 63.6, 40.6, 38.4. **IR cm^{-1}**: 3037, 3007, 2927, 1724, 1699, 1297, 1130. **HRMS** ($M+H^+$), calcd for $C_{19}H_{23}S_2O_5$ = 395.098694 , Found 395.098688. (δ = - 4.6 ppm).

1,5-Diphenyl-5-(*p*-toluenesulfonyl)-pent-1-en-3-one (26) (R= *p*-tolyl)

Sodium *p*-toluenesulfinate (455 mg, 1.2 equiv, 2.56 mmol) in water (2.5 mL) was added to solution dibenzalacetone (500 mg, 2.13 mmol) in AcOH (9.5 mL). The mixture was stirred for 2 h at rt. The resulting thick white ppte was filtered and washed with water (2 × 50 mL) and left in vacuo overnight. The resulting powder (737 mg, 89%) was the title compound in a form sufficiently pure for characterization.

White solid, m.p. 143 °C dec; **^{1}H NMR** (400 MHz, $CDCl_3$) δ 7.62 – 7.47 (m, 3H), 7.46-7.34 (m, 5H), 7.29 – 7.13 (m, 7H), 6.69 (dd, *J* = 16.2, 1.4 Hz, 1H), 4.85 (ddd, *J* = 9.7, 3.9, 1.4 Hz, 1H), 3.81 (ddd, *J* = 17.5, 3.9, 1.4 Hz, 1H), 3.62 (ddd, *J* = 17.5, 9.7, 1.4 Hz, 1H), 2.38 (s, 3H). **^{13}C NMR** (101 MHz, $CDCl_3$) δ 194.7, 144.7, 143.9, 134.1, 134.0, 132.6, 130.9, 129.8, 129.4, 129.1, 129.0, 128.8, 128.4,128.4, 125.6, 66.4, 38.8, 21.6. **IR cm^{-1}**: 3065, 3033, 2941, 2941, 2921, 1666, 1313, 1289, 1141. **HRMS** ($M+H^+$), calcd for $C_{24}H_{23}SO_3$ = 391.136792, Found 391.13616. (δ = - 1.6 ppm)

1,5-Diphenyl-1,5-bis(*p*-toluenesulfonyl)-pentan-3-one (27) (R = *p*-tolyl)

Sodium *p*-toluenesulfinate (0.76 g, 4.0 equiv, 4.3 mmol) was added to a solution of dibenzalacetone (500 mg, 2.13 mmol) in AcOH (9.5 mL) and water (1.25 mL). The mixture was stirred overnight at 50 °C. The resulting thick white ppte was filtered and washed with water (2 × 50 mL) and left in vacuo overnight. The resulting powder (486 mg, 84%) was the title compound in a form sufficiently pure for characterization, isolated as a 4:3 mixture of diastereomers.

White solid, m.p. 167 °C dec; **^{1}H NMR** (400 MHz, $CDCl_3$) δ 7.48 – 6.83 (m, 18H, superposed), 4.58 (dd, *J* = 9.3, 4.5 Hz, 2H. superposed), 3.59 (dd, *J* = 17.8, 4.5 Hz, 2H, major), 3.51 (dd, *J* = 17.8, 4.5 Hz, 2H, minor), 3.38 (dd, *J* = 17.8, 9.3 Hz, 2H, minor), 3.18 (dd, 2H, *J* = 17.8, 9.3 Hz major), 2.38 (s, 6H, major), 2.36 (s, 6H, major). **^{13}C NMR** (101 MHz, $CDCl_3$) δ Major 201.1, 144.9, 133.7, 131.9, 129.4, 129.4, 129.0, 128.8, 128.4, 65.9, 41.6, 21.6. Minor: 200.9, 144.8, 133.6, 132.1, 129.5,129.3, 129.0, 128.9, 128.5, 66.0, 41.3, 21.6. **IR cm^{-1}**:3065, 3033, 2946, 1719, 1303, 1144. **HRMS** ($M+H^+$), calcd for $C_{31}H_{31}S_2O_5$ = 547.161294, Found 547.15927 (δ = - 3.7 ppm).

3,3'-Sulfonylbis[1,3-diphenyl-1-propanone] (30)[15]

Rongalite (1.0 equiv, 2.4 mmol, 370 mg) was added to a solution of *trans*-chalcone (2.0 equiv, 4.8 mmol, 1.0 g) in acetic acid (24 mL) and water (6 mL). The mixture was heated to 50 °C and stirred overnight. The solvent was removed at reduced pressure and the crude mixture partitioned between EtOAc and water (20 mL each). The layers were separated and the aqueous layer extract with EtOAc (2 × 20 mL). The organic extracts were combined, dried (Na_2SO_4) and the solvent removed at reduce pressure to give 900 mg of a crude solid. Chromatography (5% EtOAc/hexanes to 20% EtOAc/hexanes) gave 420 mg starting chalcone, followed by 280 mg (24%) of a white solid that was identified as a 1:1 mixture of sulfone diastereomers. Recrystallization gave an analytical sample that was a 2.7:1 mixture of diastereomers. It was unclear which diastereomer had crystallized in the majority.

m.p. 158 °C dec. (Acetone); **^{1}H NMR** (400 MHz, $CDCl_3$) Major: δ 7.94 (d, *J* = 7.8 Hz, 4H), 7.76 – 7.19 (m, 16H), 4.78 (d, J = 9.6 Hz, 2H), 4.12-3.97 (superposed) (m, 2H), 3.90-3.76 (m, 2H) (superposed). Minor: δ 7.96 (d, *J* = 7.8 Hz, 4H), 7.76 – 7.19 (m, 16H), 5.13 (dd, *J* = 9.6, 4.1 Hz, 2H), 4.12-3.97 (superposed) (m, 2H), 3.90-3.76 (m, 2H) (superposed). **^{13}C NMR** (101 MHz, $CDCl_3$) Major: δ 194.7, 136.1, 133.6, 132.7, 130.2, 129.3, 128.8, 128.7, 128.1, 61.2, 37.7. Minor: 194.9, 136.1, 133.7. 132.5, 129.9, 129.1, 128.9, 128.7, 128.2, 62.3, 38.5. **IR cm^{-1}**: 3063, 3031, 2918, 2852, 1680, 1290, 1232, 1127. **HRMS** (M+H$^+$), calcd for $C_{30}H_{27}SO_4$ = 483.163007, Found 483.16165 (δ = - 2.8 ppm).

4,4′-Sulfonylbis[4-phenyl-2-butanone] (32)

Rongalite (1.40 g, 9.1 mmol) was added to a solution of benzalacetone (4-phenyl-3-buten-2-one, 4.0 g, 27.3 mmol) in acetic acid (100 mL) and water (10 mL). The mixture was heated to 50 °C and stirred overnight. The acetic acid and water were removed at reduced pressure. The residue was dissolved in EtOAc (50 mL), washed with saturated aq. $NaHCO_3$, separated and dried (Na_2SO_4). Removal of the solvent gave a crude oil that showed 10–15% conversion by ^{1}H NMR. Chromatography 10% EtOAc/hexanes to 40% EtOAc/hexanes) gave 2.2 g of recovered starting material and 420 mg of a white solid (13%) that was recrystallized from EtOAc/hexanes and was revealed to be a 1:1 mixture of diastereomers. M.p. 116-117 °C.

^{1}H NMR (400 MHz, $CDCl_3$) **Isomer 1:** δ 7.45 – 7.20 (m, 10H), 4.42 (dd, *J* = 9.5, 3.7 Hz, 2H), 3.50 (dd, *J* = 17.9, 3.7 Hz, 2H), 3.14-3.01 (m, 2H) (superposed), 2.03 (s, 3H). **Isomer 2:** δ 7.45 – 7.20 (m, 10H), 4.78 (dd, *J* = 8.2, 5.2 Hz, 2H), 3.39 (ddd, *J* = 17.9, 5.2 Hz, 2H), 3.14-3.01 (m, 2H) (superposed), 2.14 (s, 3H). **^{13}C NMR** (101 MHz, $CDCl_3$) Isomer 1: δ 202.9, 132.3, 130.0, 129.3, 128.9, 60.7, 43.0, 42.0, 30.4. Isomer 2: δ 203.4, 132.5, 129.7, 129.3, 129.1, 128.9, 61.9, 43.0, 30.3. **IR cm^{-1}**: 3063, 3033, 2967, 2925, 1711, 1295, 1128. **HRMS** (M+H$^+$), calcd for $C_{20}H_{23}SO_4$ = 359.131707, Found 359.13001. (δ = - 4.7 ppm)

4,4′-Sulfonylbis[2-octanone] (34)

Rongalite (305 mg, 1.98 mmol) was added to a solution of 3-octen-2-one (500 mg, 0.59 mL, 3.96 mmol, 2.0 equiv) in acetic acid (20 mL) and water (5 mL). The mixture was then heated overnight at 50 °C. The mixture was partitioned between EtOAc and water (50 mL each). The organic layer separated, washed with saturated $NaHCO_3$ (50 mL) and water (50 mL) and dried (Na_2SO_4). Removal of the solvent gave 530 mg crude oil. Product m.w. = 318.48, 100% = 631 mg. Chromatography, (5% to 15% EtOAc/hexanes) gave starting material (99 mg) and then 330 mg (52%) of a pale yellow oil, identified as a 1:1 mixture of diastereomers by ^{1}H NMR.

Yellow oil; **^{1}H NMR (400 MHz, $CDCl_3$)** 1:1 mixture of diastereomers: δ 3.74 – 3.66 (m, 2H) and 3.64-3.56 (m, 2H), 3.12 (dd, *J* = 18.8, 6.6 Hz, 2H) and 3.11 (dd, *J* = 18.4, 6.6 Hz, 2H), 2.52 (dd, *J* = 18.8, 1.5 Hz, 2H) and 2.51 (dd, *J* = 18.4, 1.7 Hz, 2H), 2.24 (s, 3H) and 2.21 (s, 3H), 1.96-1.80 (m, 2H, superposed), 1.63 – 1.45 (m, 2H, superposed), 1.37 – 1.21 (m, 8H, superposed), 0.91-0.81 (6H, superposed). **^{13}C NMR (101 MHz, $CDCl_3$)** 1:1 mixture of diastereomers δ 204.9 and 204.4, 54.8 and 54.8, 41.11 and 40.4, 30.1 and 30.10, 28.7 and 28.7, 28.6 and 27.8, 22.5 and 22.4, 13.8 and 13.8. **IR cm^{-1}**: 2952, 2922, 2868, 1720, 1698, 1357, 1275, 1131. **HRMS** (M+H$^+$), calcd for $C_{16}H_{31}SO_4$ = 319.194307, Found 319.19364 (δ = - 2.1 ppm).

References

1. Pracht, P.; Bohle, F.; Grimme, S. *Phys. Chem. Chem. Phys.*, **2020**, *22*, 7169-7192.

2. Grimme, S.; Bohle, F.; Hansen, A.; Pracht, P.; Spicher, S.; Stahn, M. *J. Phys. Chem. A*, **2021**, *125*, 4039-4054.

3. Bannwarth, C.; Ehlert, S.; Grimme, S.; *J. Chem. Theo. Comp.*, **2019**, *15*, 1652-1671.

4. Perdew, J. P.; Burke, K.; Ernzerhof, M.; *Phys. Rev. Lett.*, **1996**, *77*, 3865-3868.

5. Weigend, F.; Ahlrichs, R.; *Phys. Chem. Chem. Phys.*, **2005**, *7*, 3297-3305.

6. Balasubramani, S. G.; Chen, G. P.; Coriani, S.; Diedenhofen, M.; Frank, M. S.; Franzke, Y. J. *et. al. J. Chem. Phys.*, **2020**, *152*, 184107.

7. Weigend, F.; *Phys. Chem. Chem. Phys.*, **2006**, *8*, 1057-1065.

8. Adamo, C.; Barone, V. *J. Chem. Phys.*, **1999**, *110*, 6158-6170.

9. Grimme, S.; Antony, J.; Ehrlich, S.; Krieg, H. *J. Chem. Phys.*, **2010**, *132*, 154104.

10. Caldeweyher, E.; Bannwarth, C.; Grimme, S. *J. Chem. Phys.* **2017**, *147*, 034112.

11. Neese, F. *Wiley Int. Rev.: Comp. Mol. Sci.* **2018**, *8*, e1327.

12. Goga, M.; Zong, H.; Prana, J.; Michel, R.; Muro, A.; Rubin, E.; Brenya, J.; Bebbington, M. W. P. *Synth. Commun.* **2023,** *53,* 1351–1359.

13. Rule, N. G.; Detty, M. R.; Kaeding, J. E.; Sinicropi, J. A. *J. Org. Chem.* **1995,** *60,* 1665–1673

14. Gendron, T.; Kessedjian, H.; Davioud-Charvet, E.; Lanfranchi, D. A. *Eur. J. Org. Chem.* **2015,** 1790–1796.

15. Coates, J. E. ; Abbott F. S. *J. Org. Chem.,* **1977**, *42*, 3506-3514.

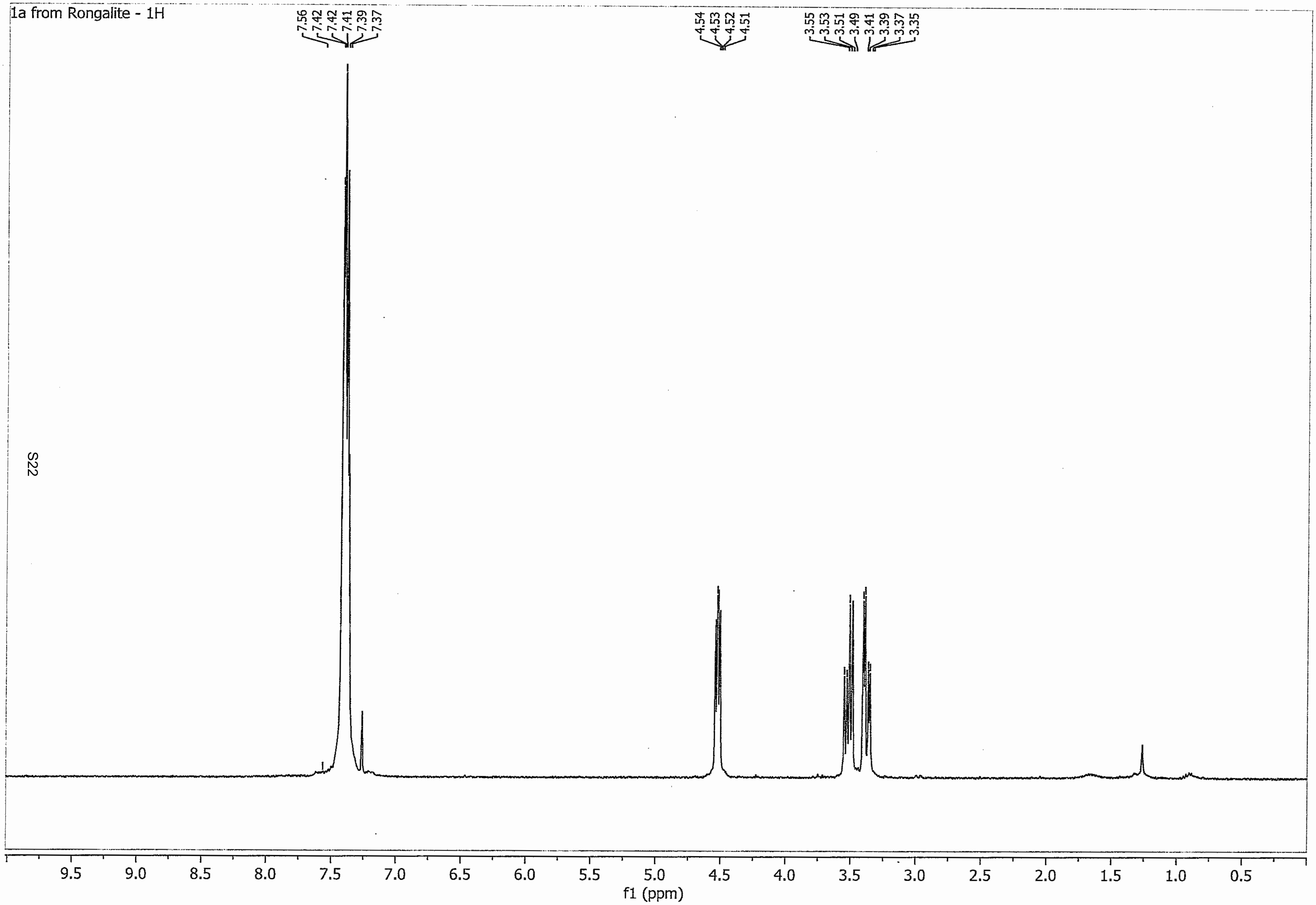
1a from Rongalite - 1H
7.56
7.42
7.42
7.41
7.39
7.37
4.54
4.53
4.52
4.51
3.55
3.53
3.51
3.49
3.41
3.39
3.37
3.35
9.5
9.0
8.5
8.0
7.5
7.0
6.5
6.0
5.5
5.0
4.5
4.0
3.5
3.0
2.5
2.0
1.5
1.0
0.5
f1 (ppm)

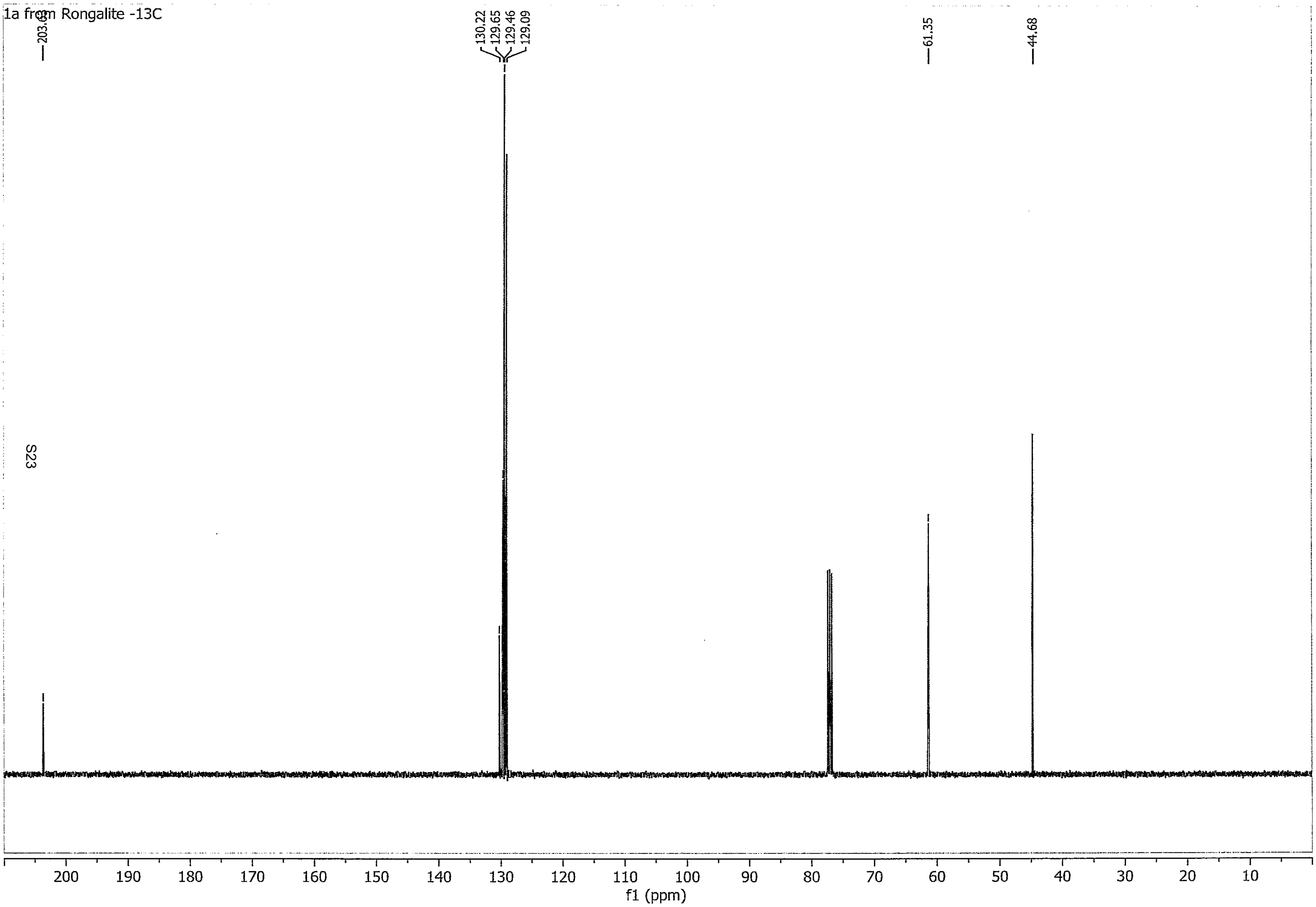
1a from Rongalite -13C
203.[illegible]
130.22
129.65
129.46
129.09
61.35
44.68
200 190 180 170 160 150 140 130 120 110 100 90 80 70 60 50 40 30 20 10
f1 (ppm)

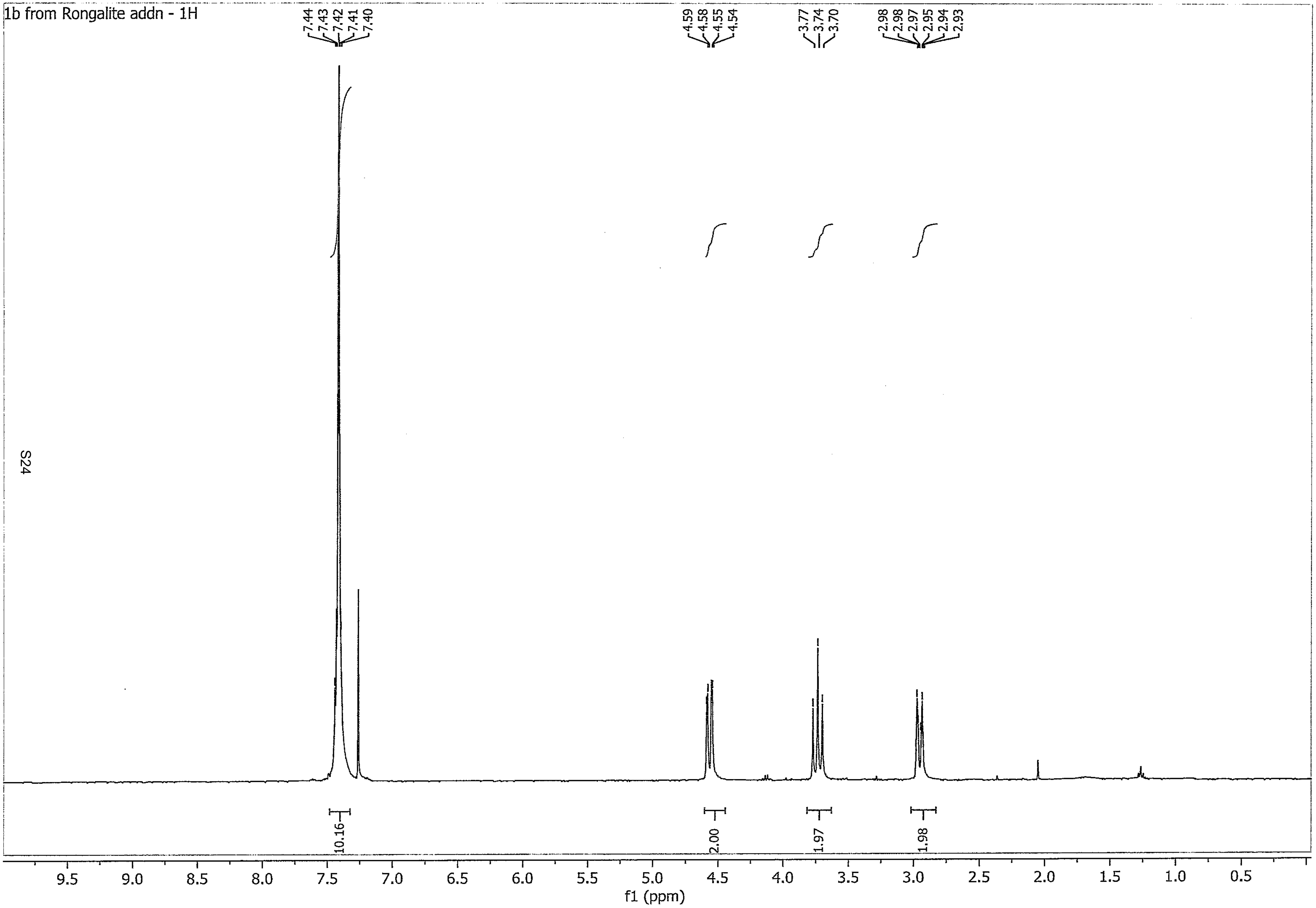
1b from Rongalite addn - 1H
7.44
7.43
7.42
7.41
7.40
4.59
4.58
4.55
4.54
3.77
3.74
3.70
2.98
2.98
2.97
2.95
2.94
2.93
10.16
2.00
1.97
1.98
9.5 9.0 8.5 8.0 7.5 7.0 6.5 6.0 5.5 5.0 4.5 4.0 3.5 3.0 2.5 2.0 1.5 1.0 0.5
f1 (ppm)

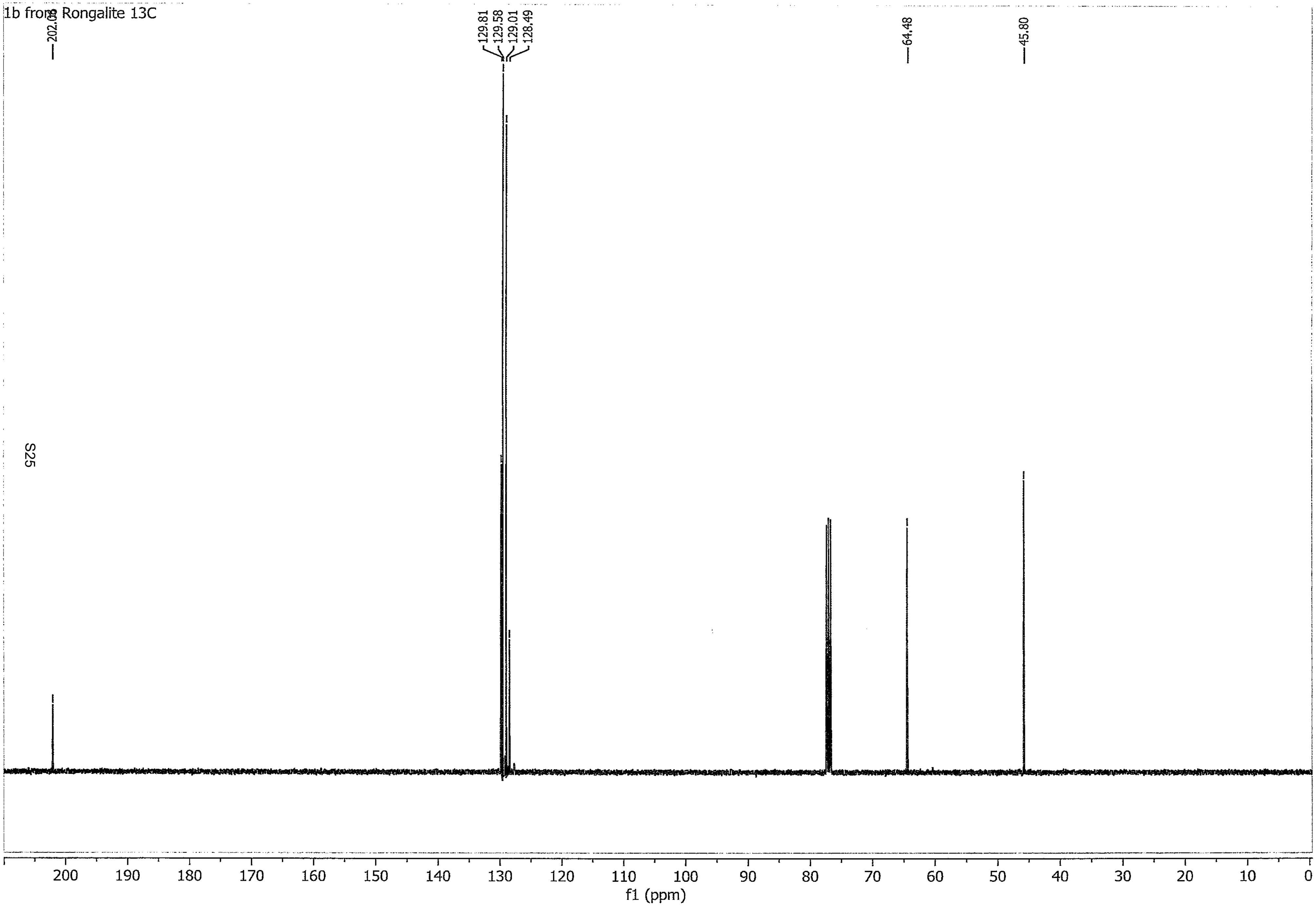
1b from Rongalite 13C
129.81
129.58
129.01
128.49
64.48
45.80
200
190
180
170
160
150
140
130
120
110
100
90
80
70
60
50
40
30
20
10
0
f1 (ppm)

3 - reductive amination

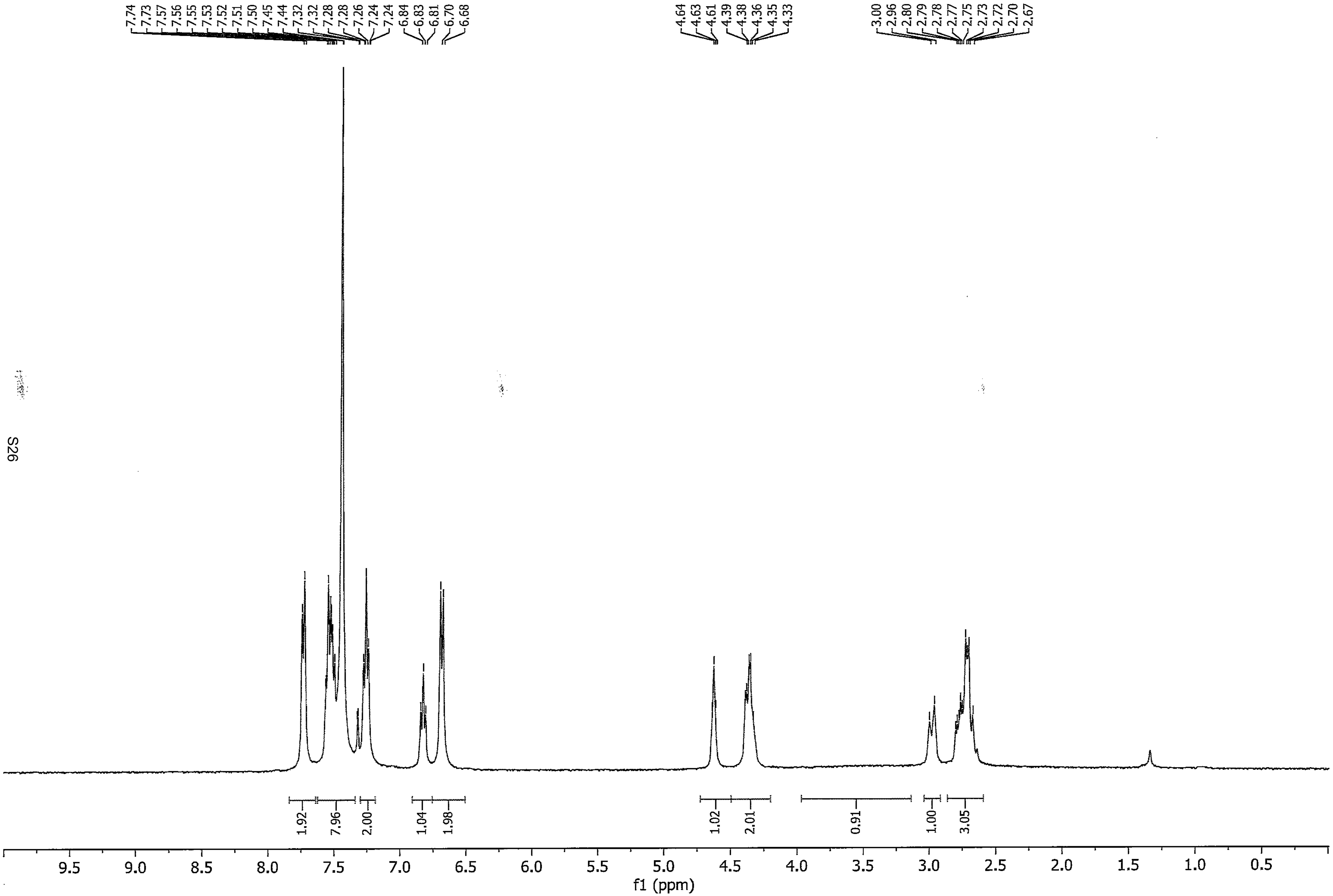

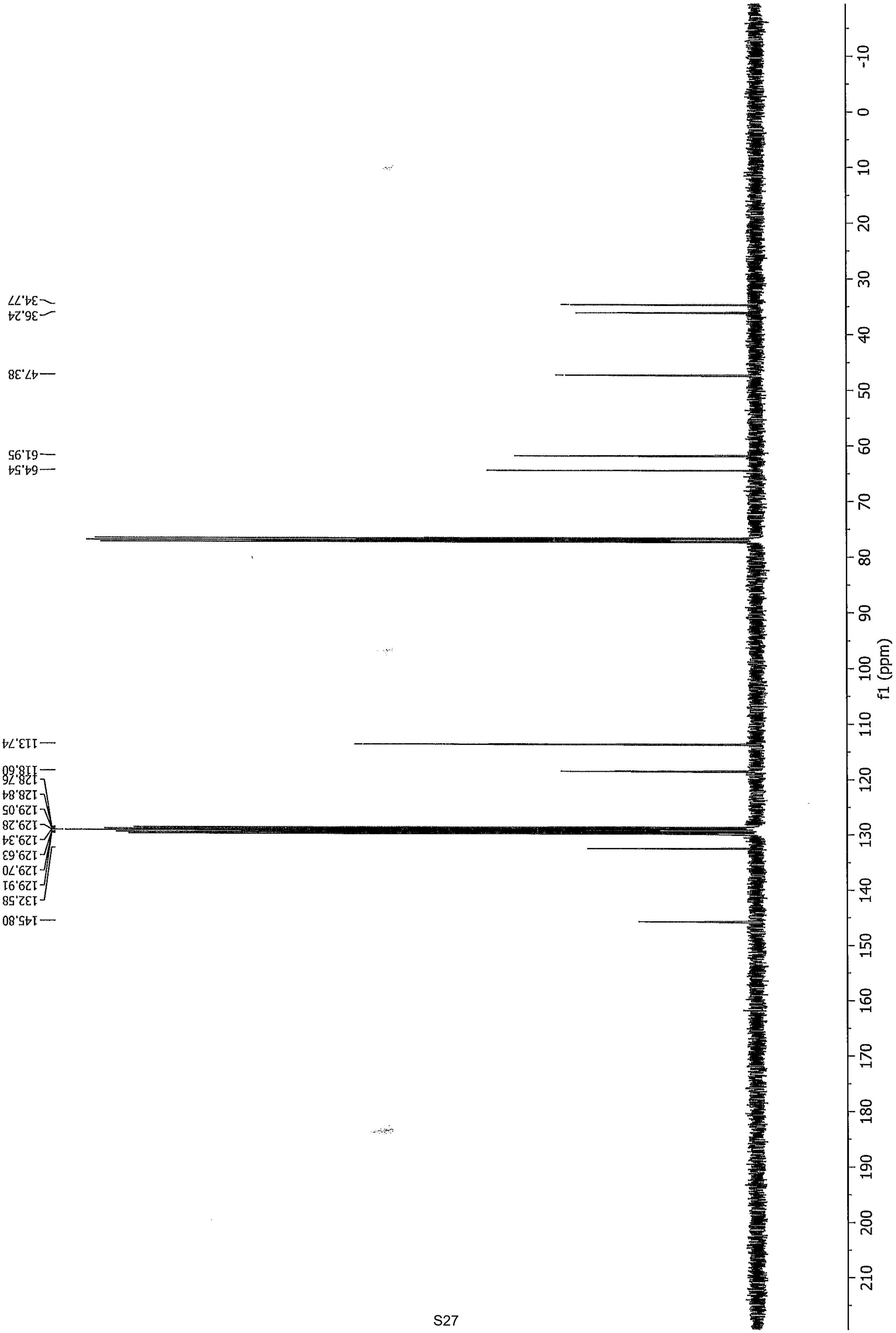
34,77
36,24
47,38
61,95
64,54
113,74
118,60
128,76
128,84
129,05
129,28
129,34
129,63
129,70
129,91
132,58
145,80
-10
0
10
20
30
40
50
60
70
80
90
100
110
120
130
140
150
160
170
180
190
200
210
f1 (ppm)

3

4- alcohol from trans sulfone

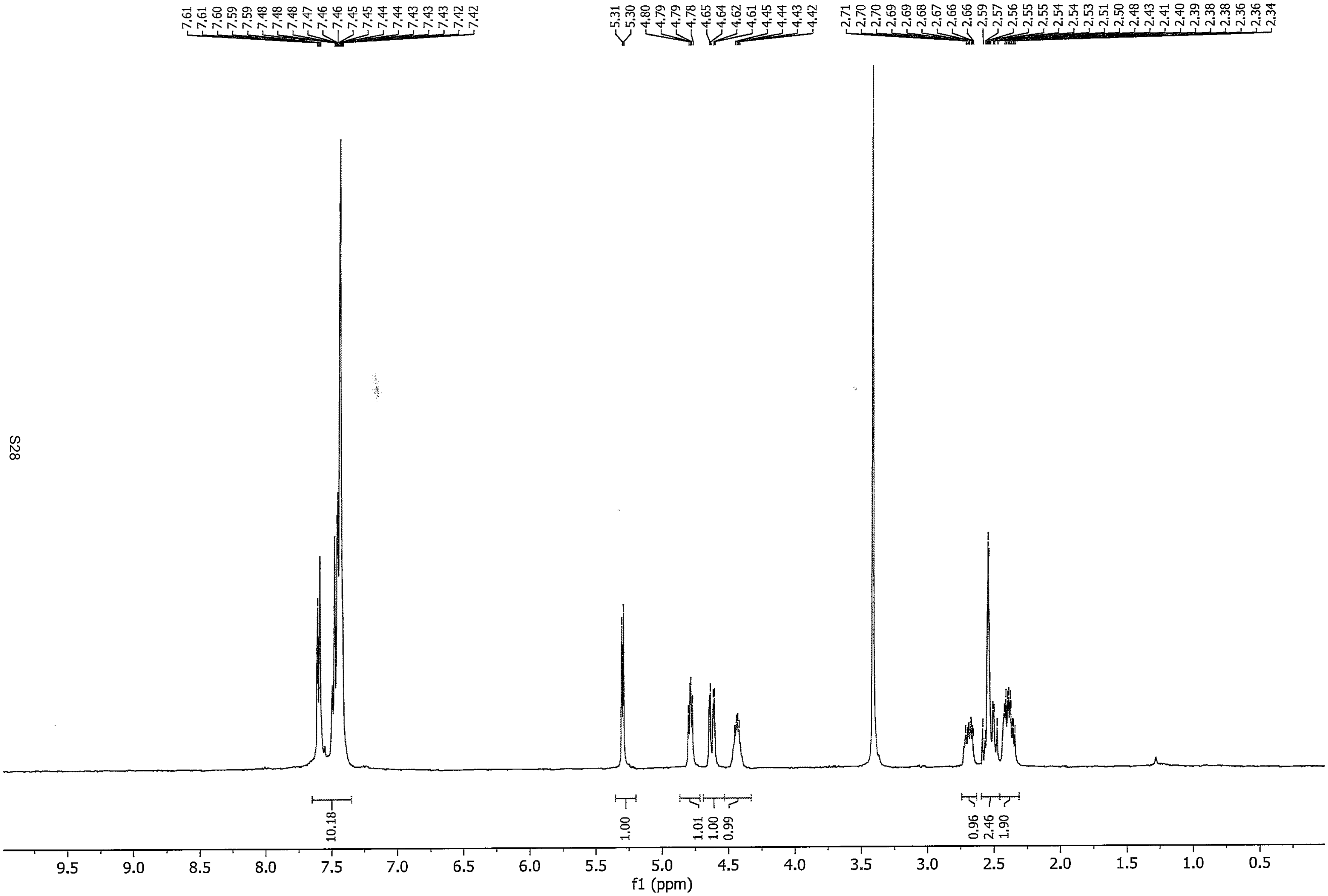

4

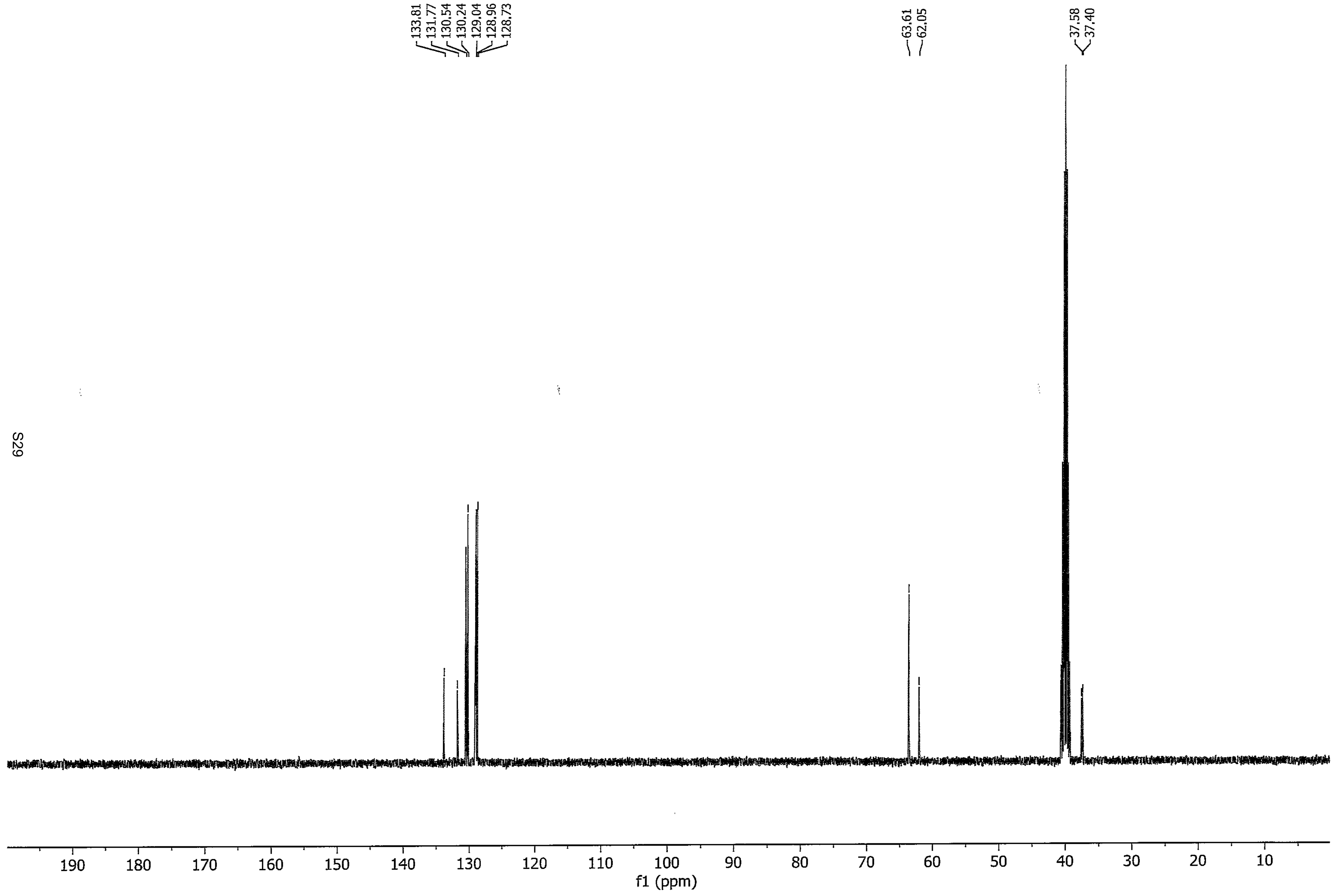
133.81
131.77
130.54
130.24
129.04
128.96
128.73
63.61
62.05
37.58
37.40
190
180
170
160
150
140
130
120
110
100
90
80
70
60
50
40
30
20
10
f1 (ppm)

5 - alcohol from cis sulfone - 1H

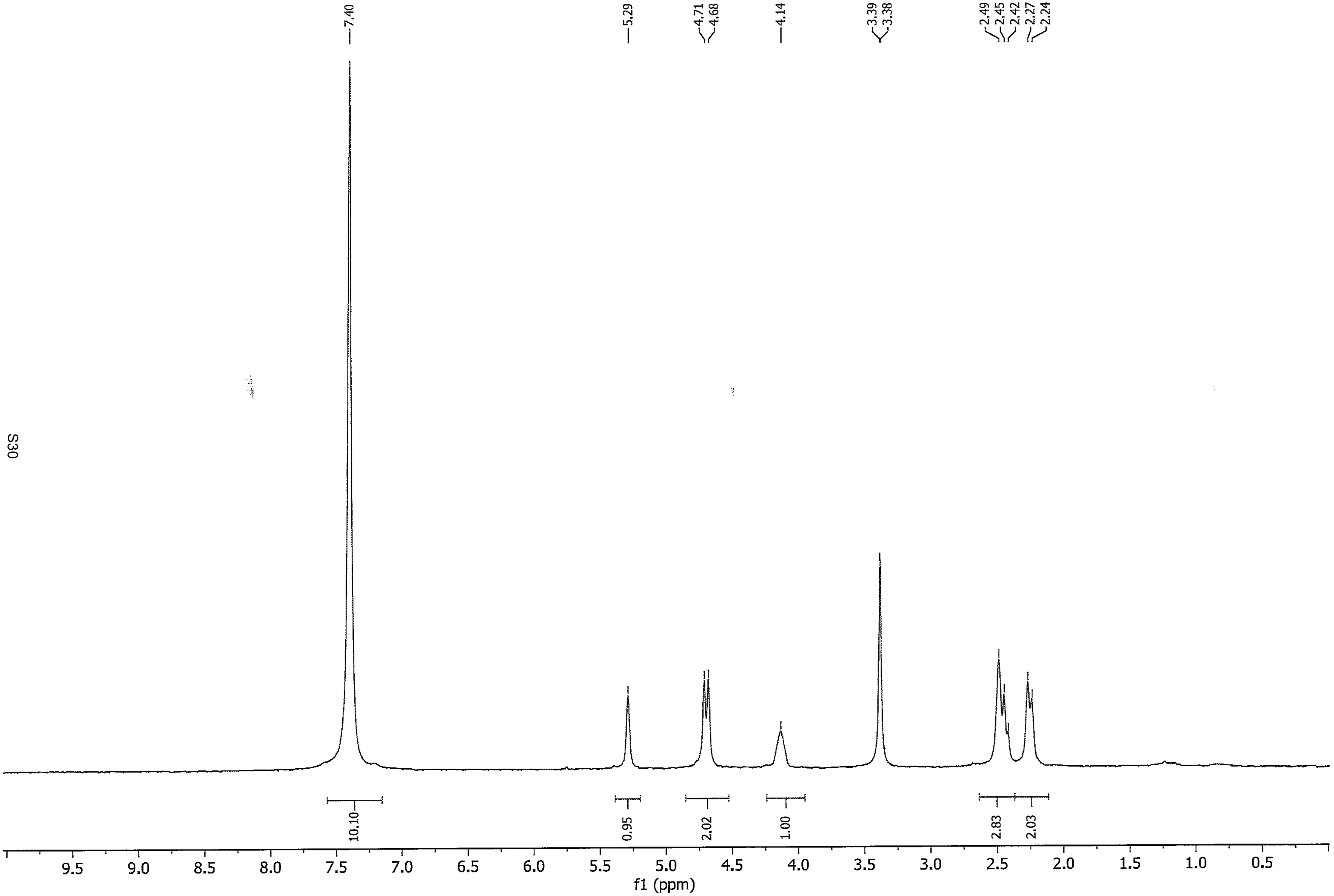

5 - 13C

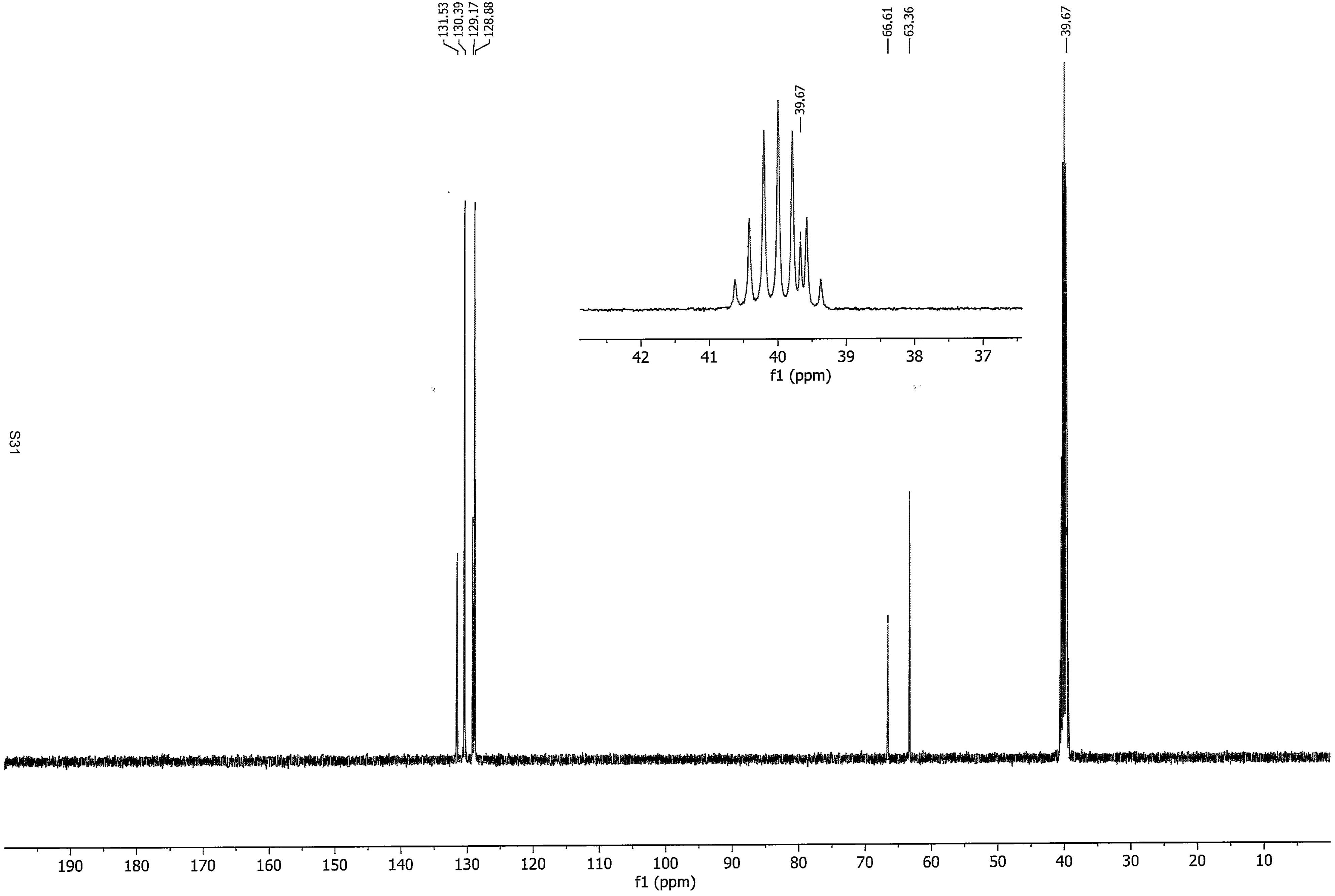
131.53
130.39
129.17
128.88
66.61
63.36
39.67
39.67
42
41
40
39
38
37
f1 (ppm)
190
180
170
160
150
140
130
120
110
100
90
80
70
60
50
40
30
20
10
f1 (ppm)

7 - 1H

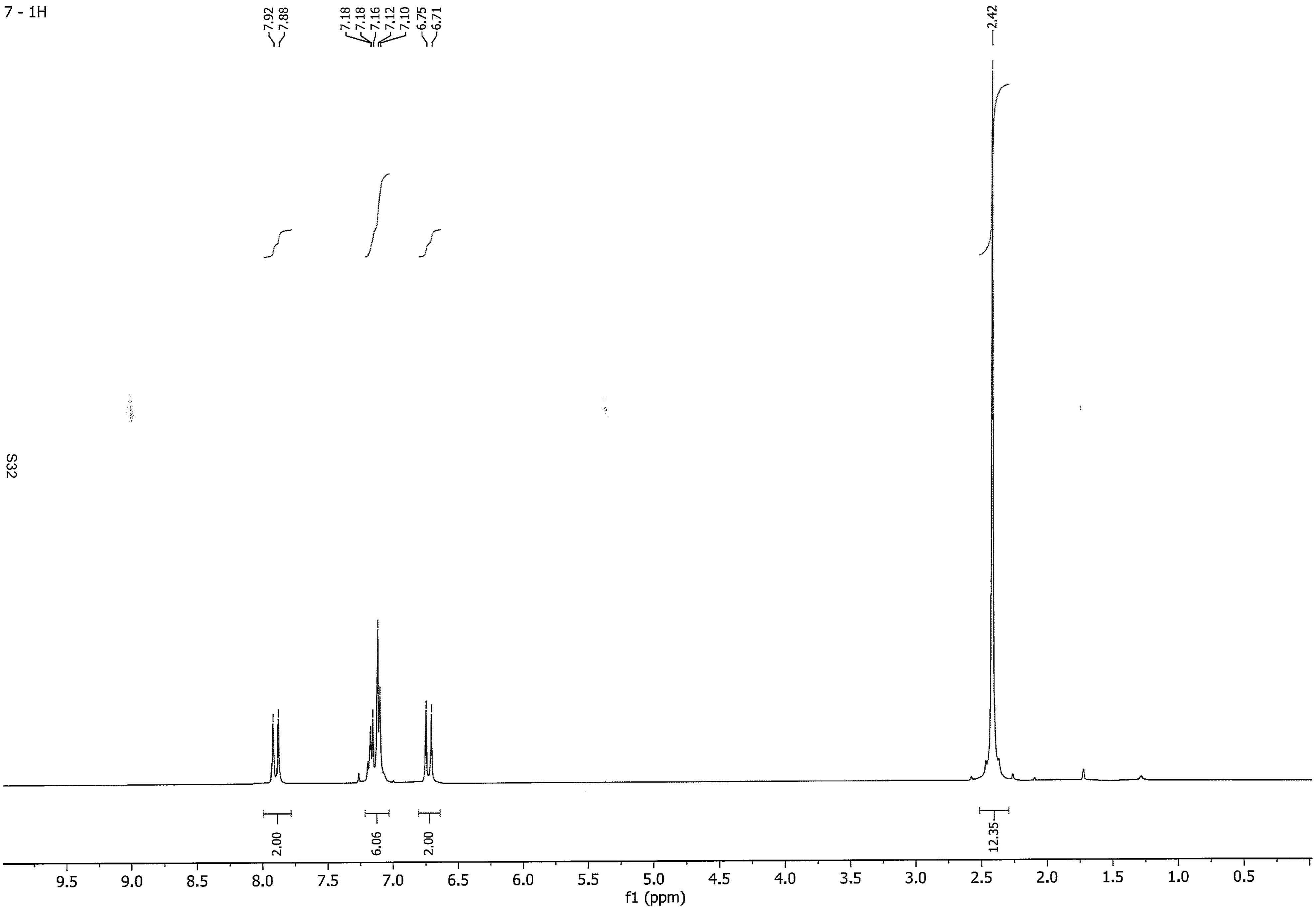
7.92
7.88
7.18
7.18
7.16
7.12
7.10
6.75
6.71
2.42
2.00
6.06
2.00
12.35
9.5
9.0
8.5
8.0
7.5
7.0
6.5
6.0
5.5
5.0
4.5
4.0
3.5
3.0
2.5
2.0
1.5
1.0
0.5
f1 (ppm)

7 - 13C

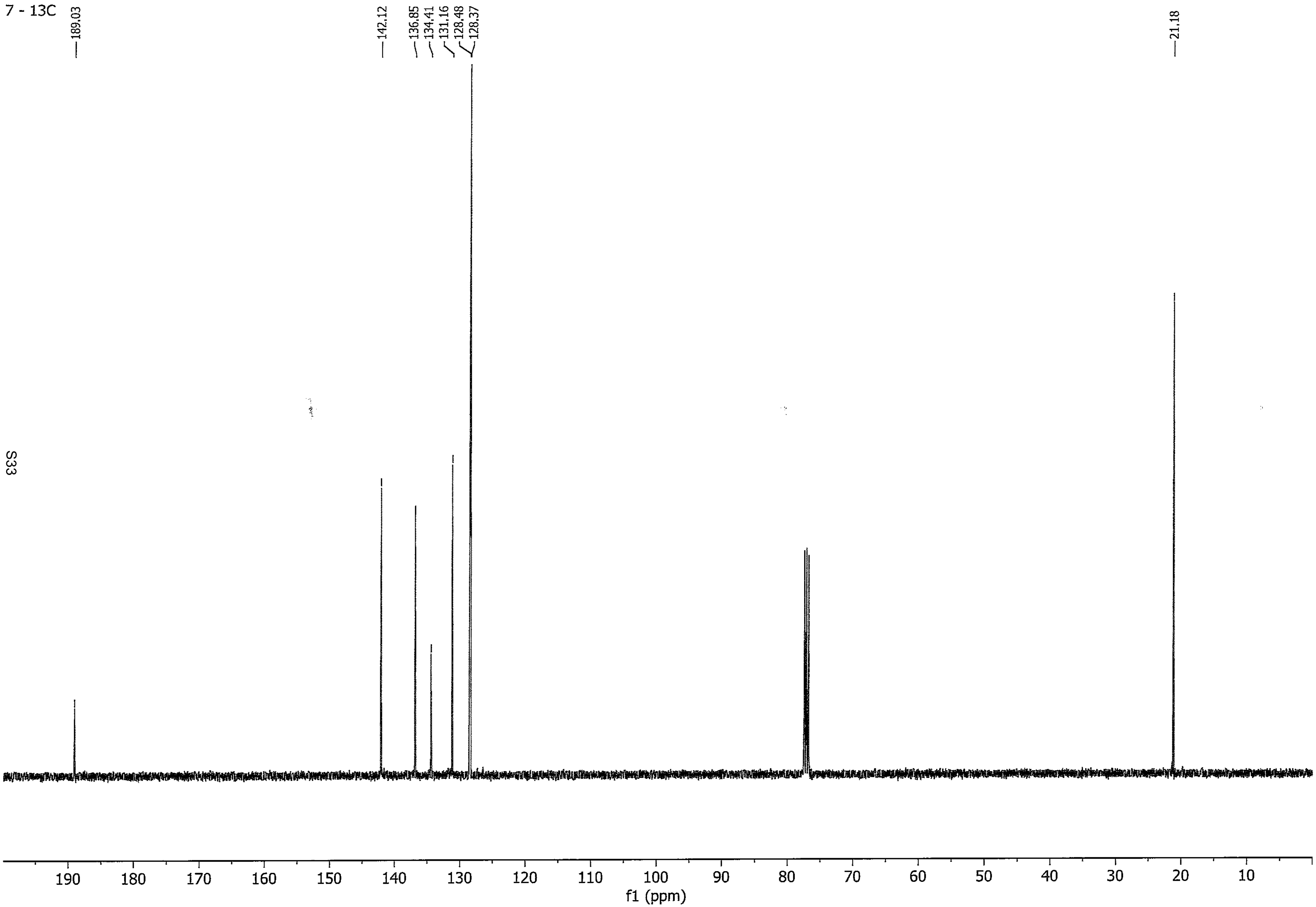
189.03
142.12
136.85
134.41
131.16
128.48
128.37
21.18
190
180
170
160
150
140
130
120
110
100
90
80
70
60
50
40
30
20
10
f1 (ppm)

9 - 1H

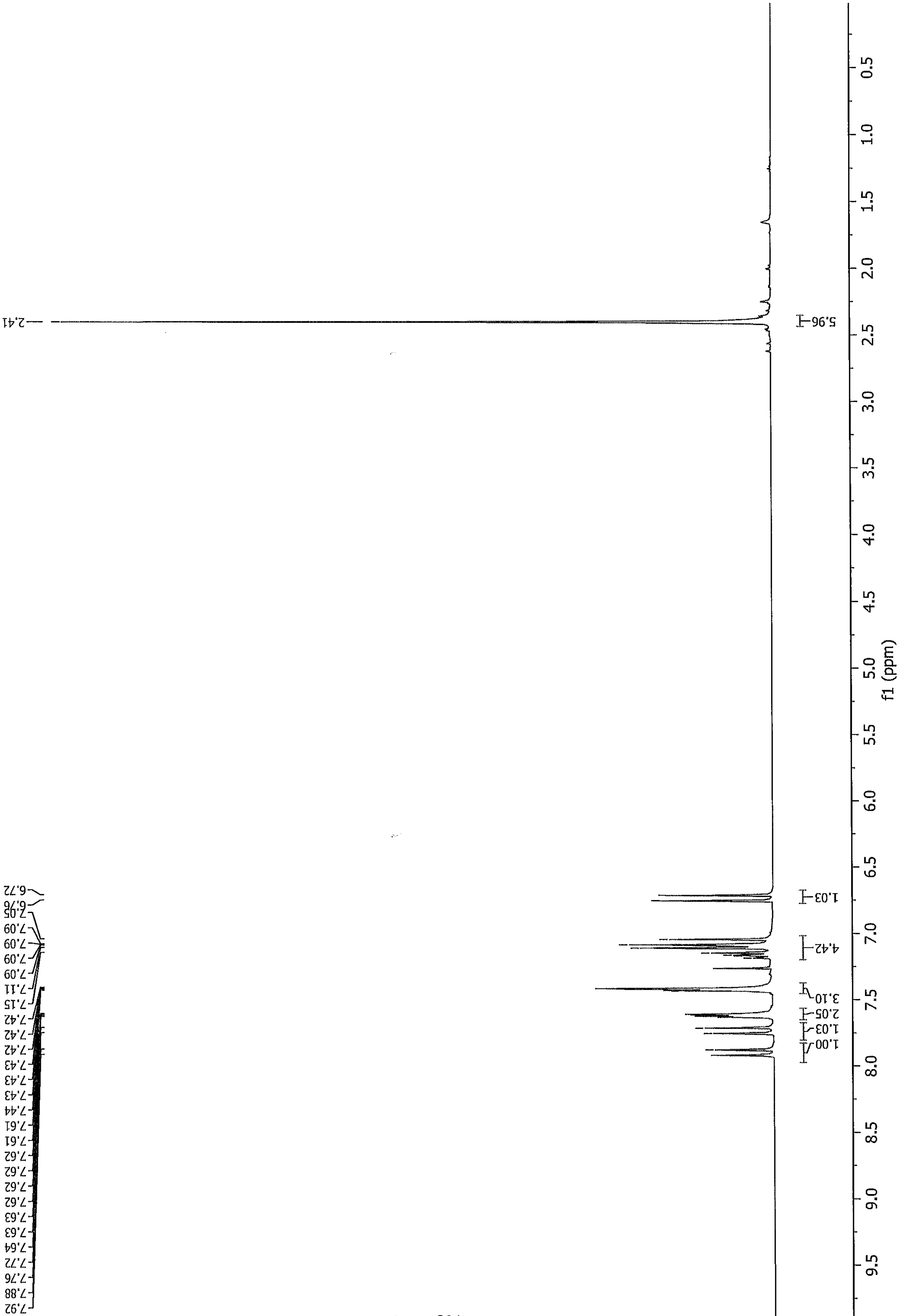
2.41
6.72
6.76
7.05
7.09
7.09
7.09
7.09
7.11
7.15
7.42
7.42
7.42
7.43
7.43
7.43
7.44
7.61
7.61
7.62
7.62
7.62
7.62
7.63
7.63
7.64
7.72
7.76
7.88
7.92
5.96
1.03
4.42
3.10
2.05
1.03
1.00
0.5
1.0
1.5
2.0
2.5
3.0
3.5
4.0
4.5
5.0
5.5
6.0
6.5
7.0
7.5
8.0
8.5
9.0
9.5
f1 (ppm)

9 - 13C

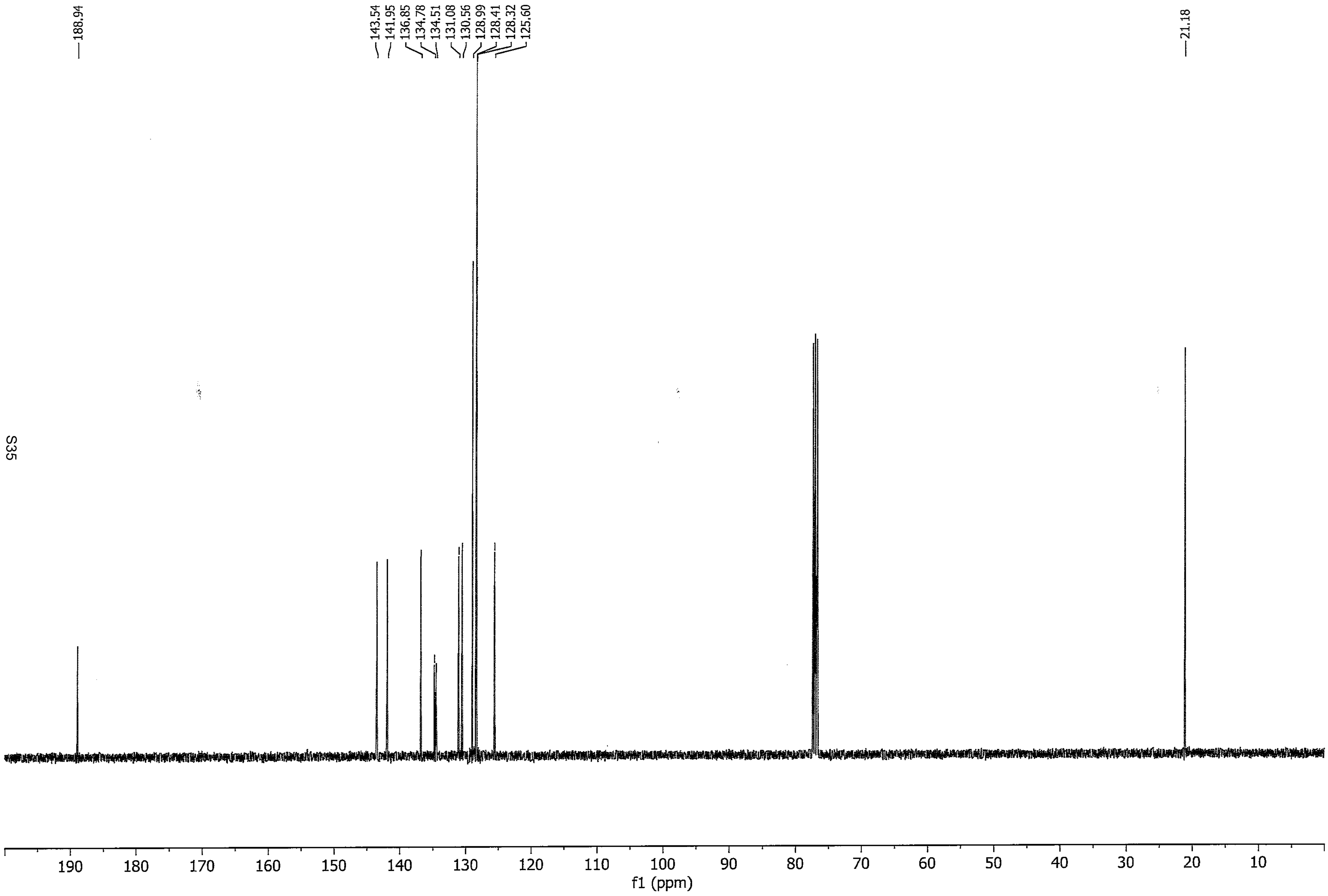
188.94
143.54
141.95
136.85
134.78
134.51
131.08
130.56
128.99
128.41
128.32
125.60
21.18
190
180
170
160
150
140
130
120
110
100
90
80
70
60
50
40
30
20
10
f1 (ppm)

10a (trans) w/restricted rotation - 1H

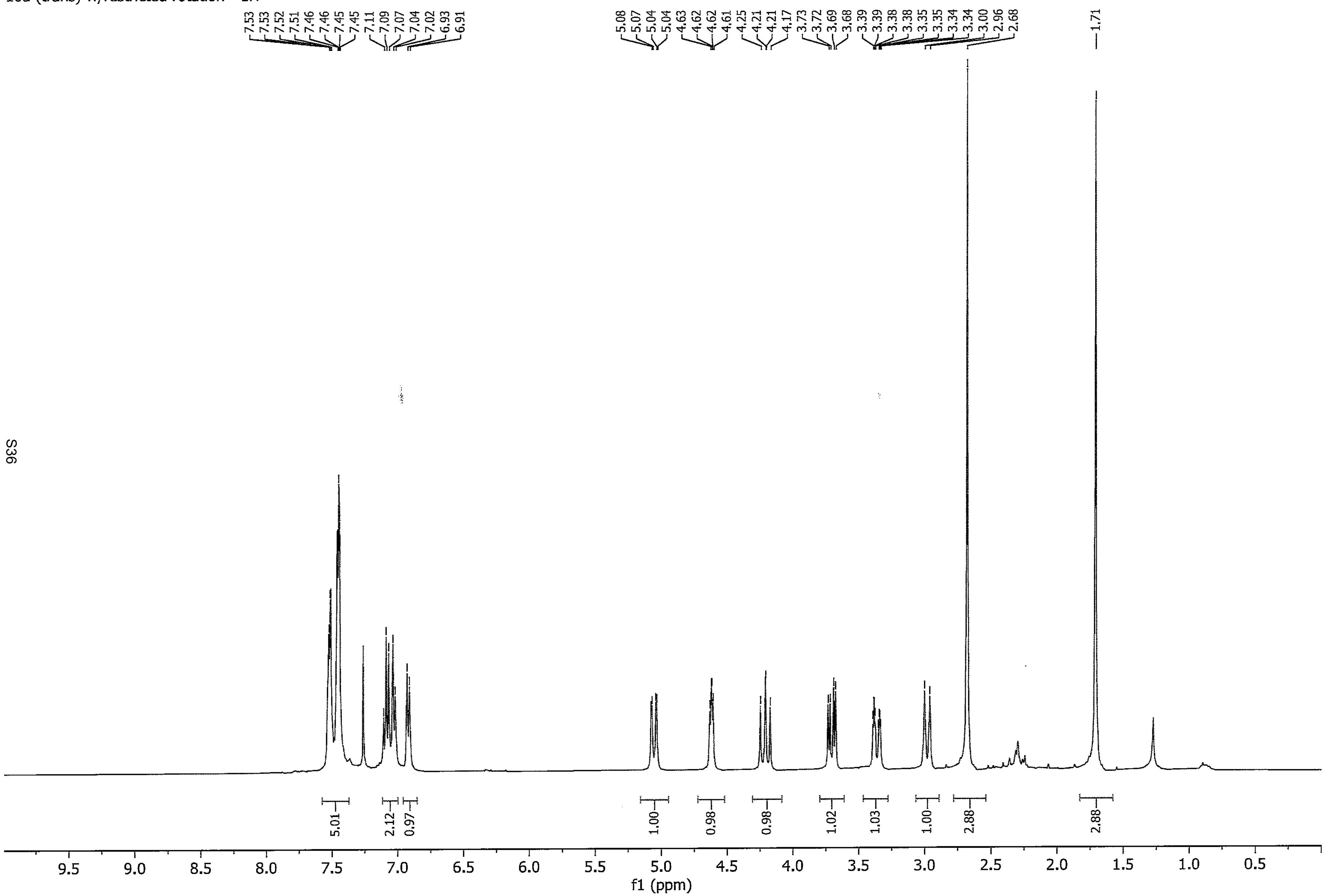

10a - 13C

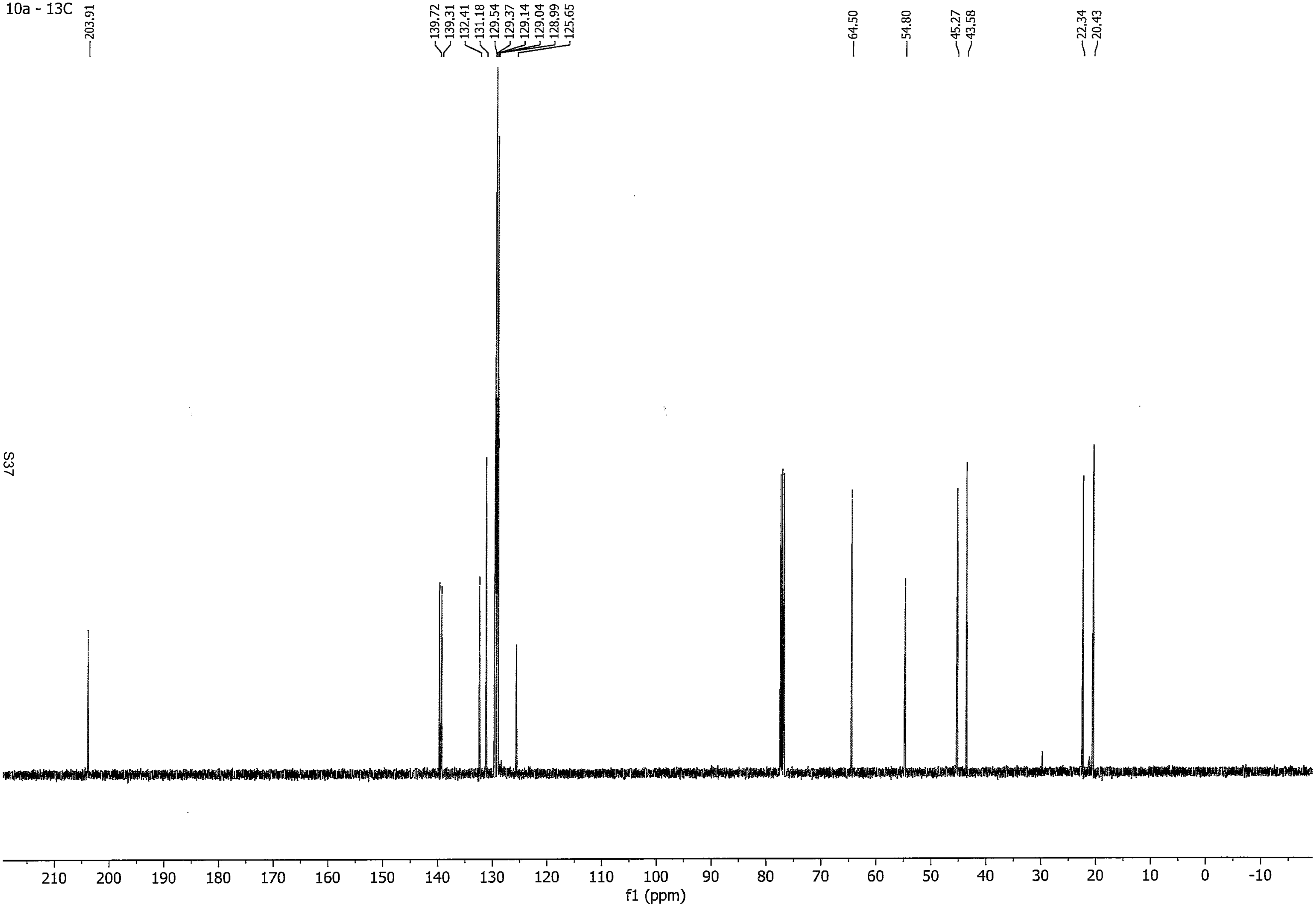

203.91
139.72
139.31
132.41
131.18
129.54
129.37
129.14
129.04
128.99
125.65
64.50
54.80
45.27
43.58
22.34
20.43
210
200
190
180
170
160
150
140
130
120
110
100
90
80
70
60
50
40
30
20
10
0
-10
f1 (ppm)

crude mixture Rongalite addition to 11 - no sign of cis isomer

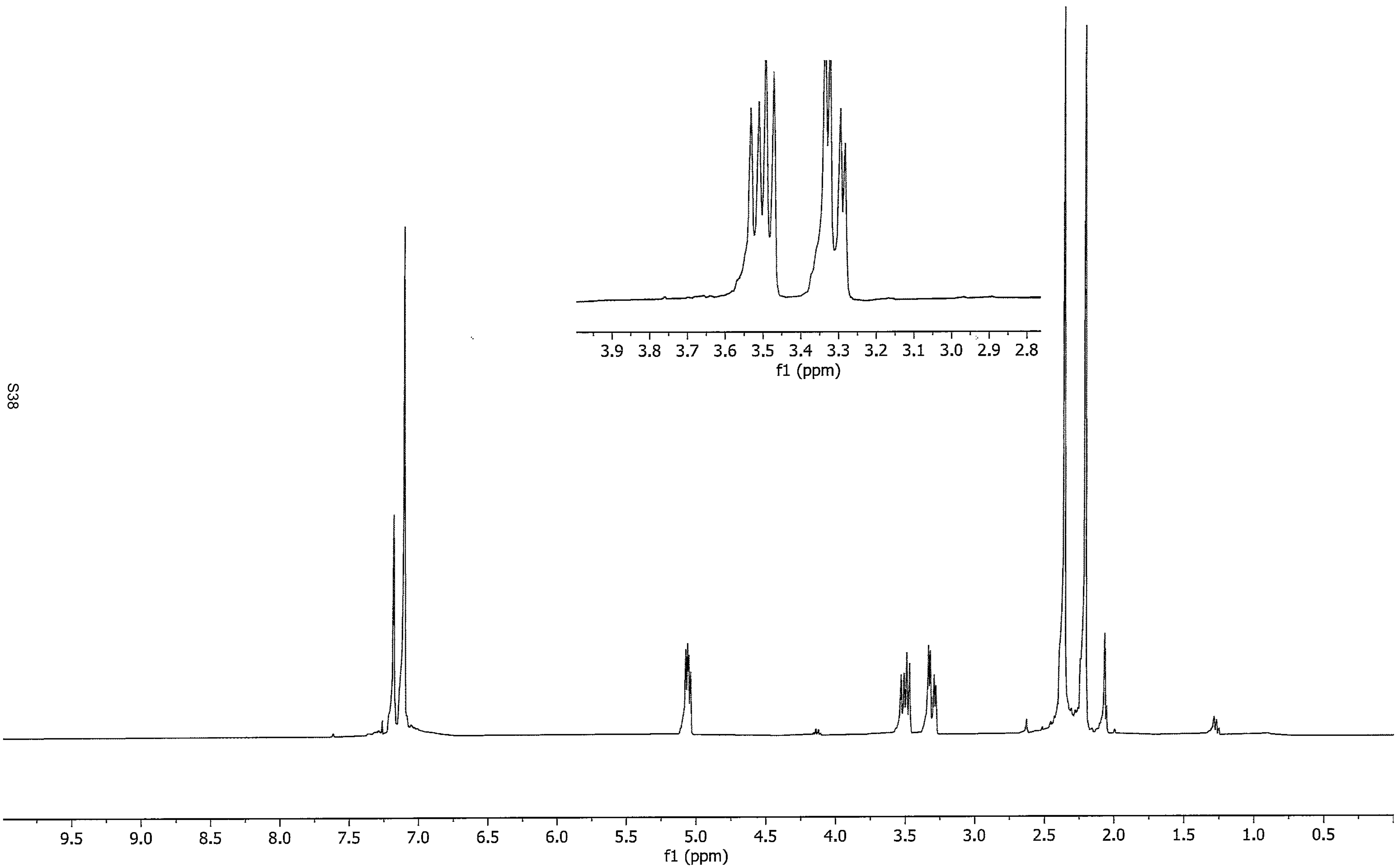

12a - trans-(2,5-dimethylphenyl) 1H

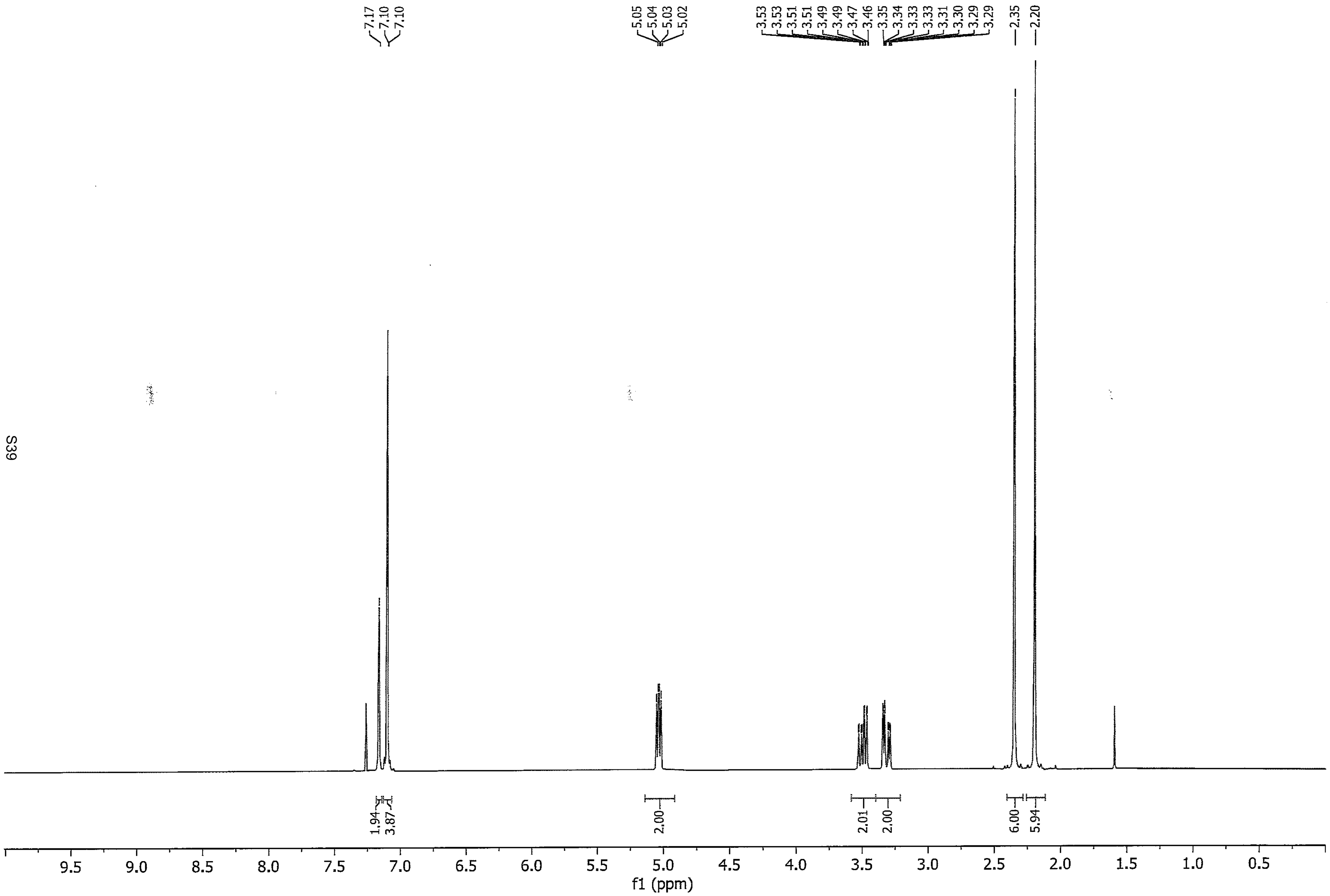

12a - 13C

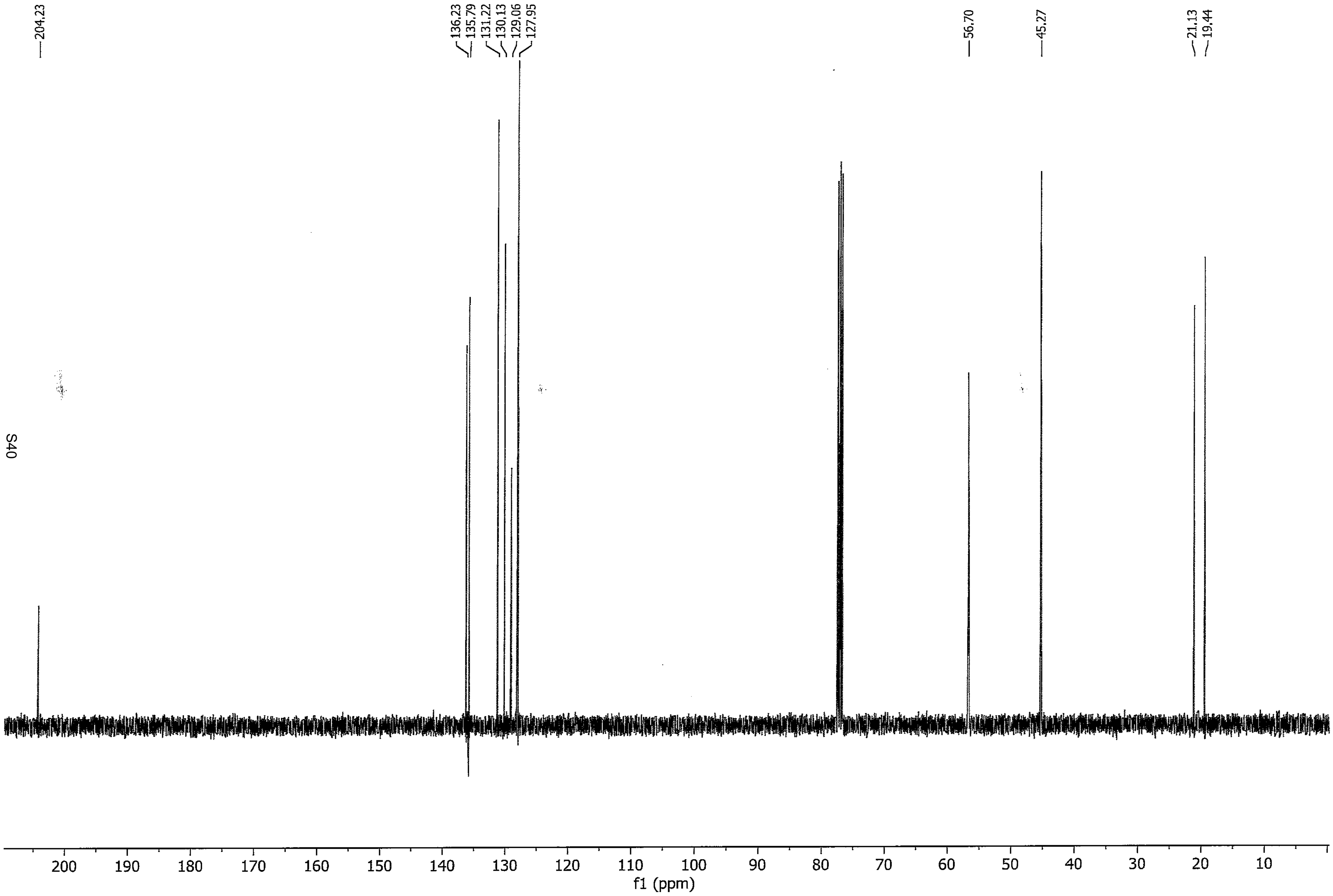
204,23
136,23
135,79
131,22
130,13
129,06
127,95
56,70
45,27
21,13
19,44
200 190 180 170 160 150 140 130 120 110 100 90 80 70 60 50 40 30 20 10
f1 (ppm)

24 - methanesulfonyl monoadduct 1H

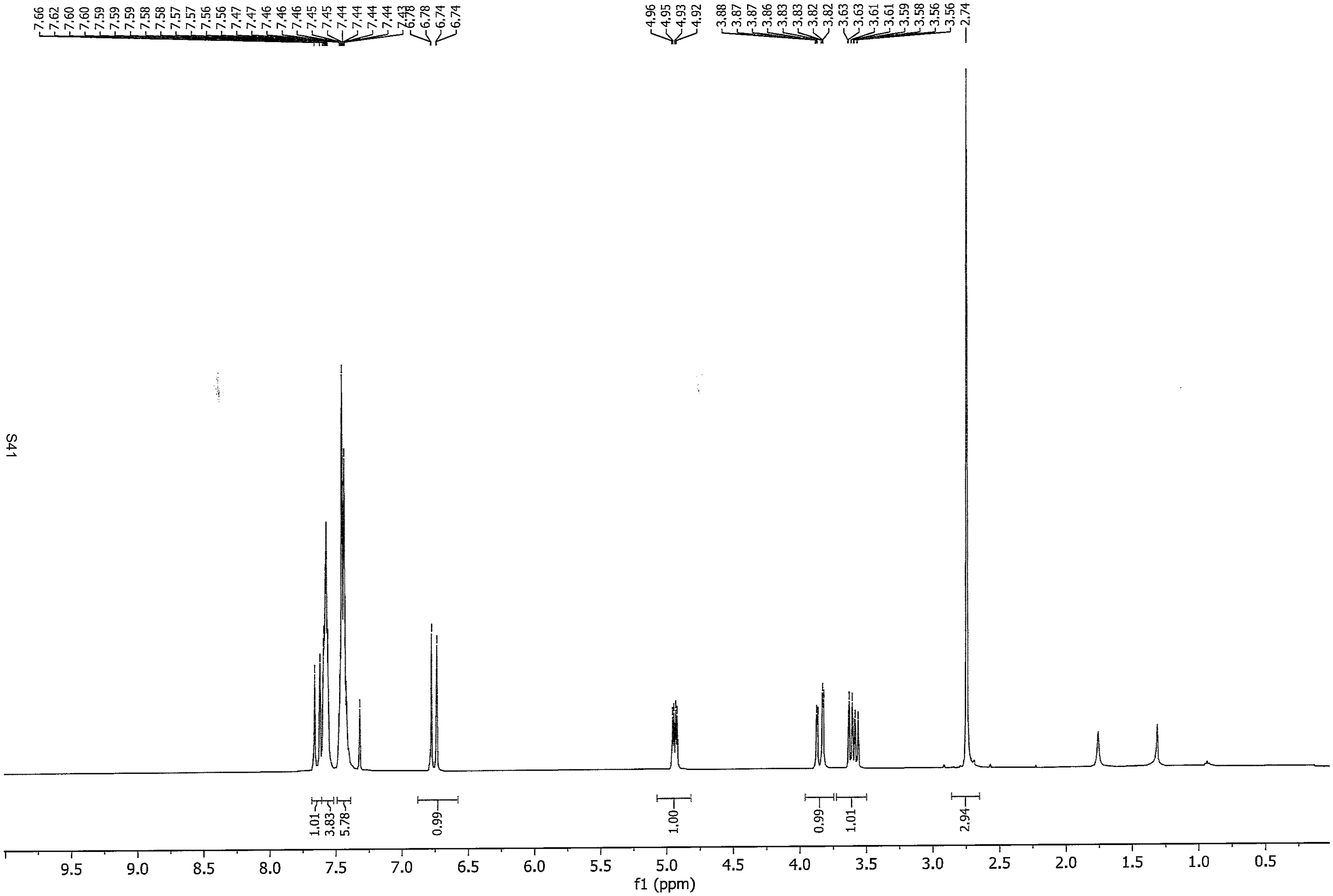

24 - 13C

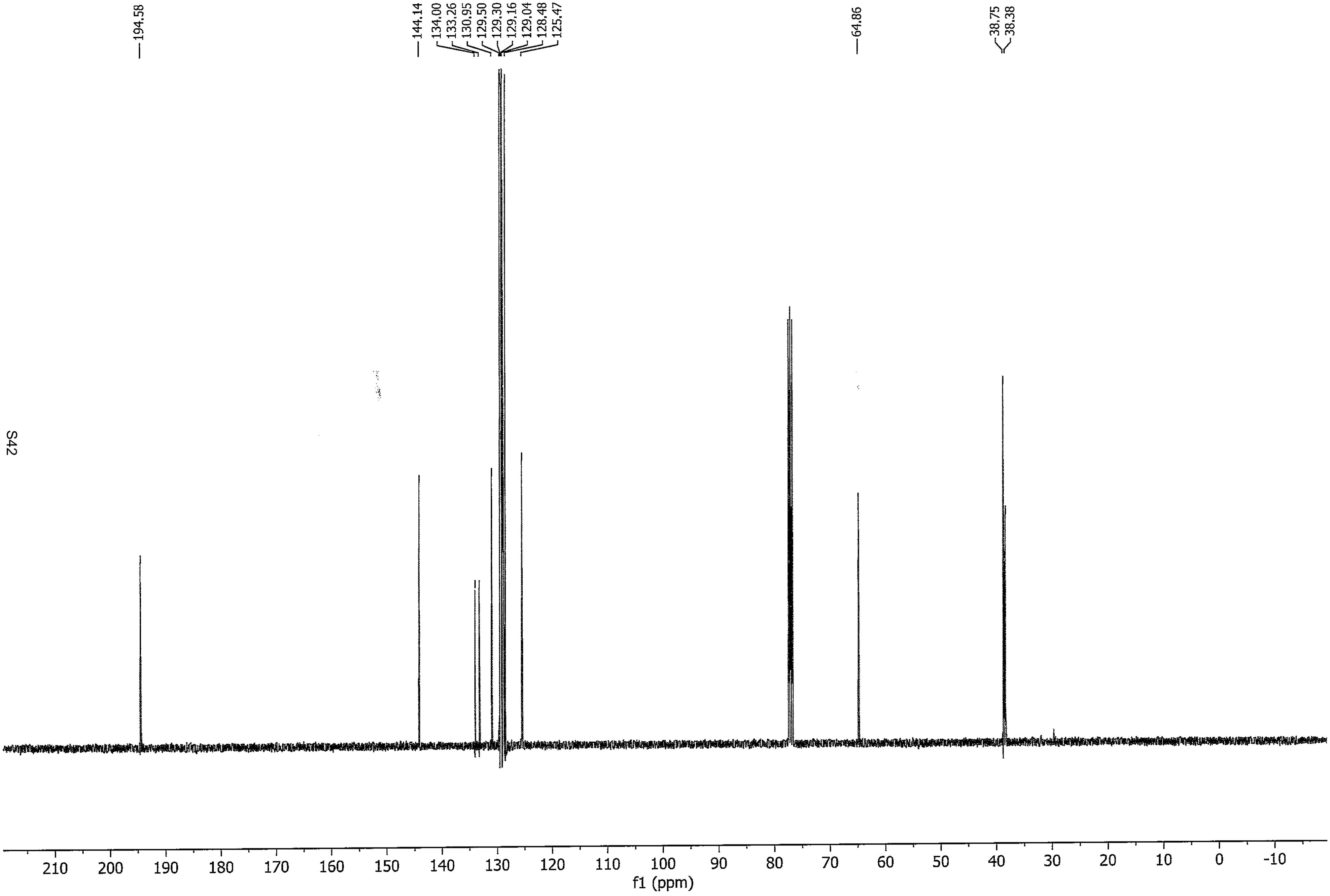
194.58
144.14
134.00
133.26
130.95
129.50
129.30
129.16
129.04
128.48
125.47
64.86
38.75
38.38
210 200 190 180 170 160 150 140 130 120 110 100 90 80 70 60 50 40 30 20 10 0 -10
f1 (ppm)

25 - methanesulfonyl_diadduct 1H

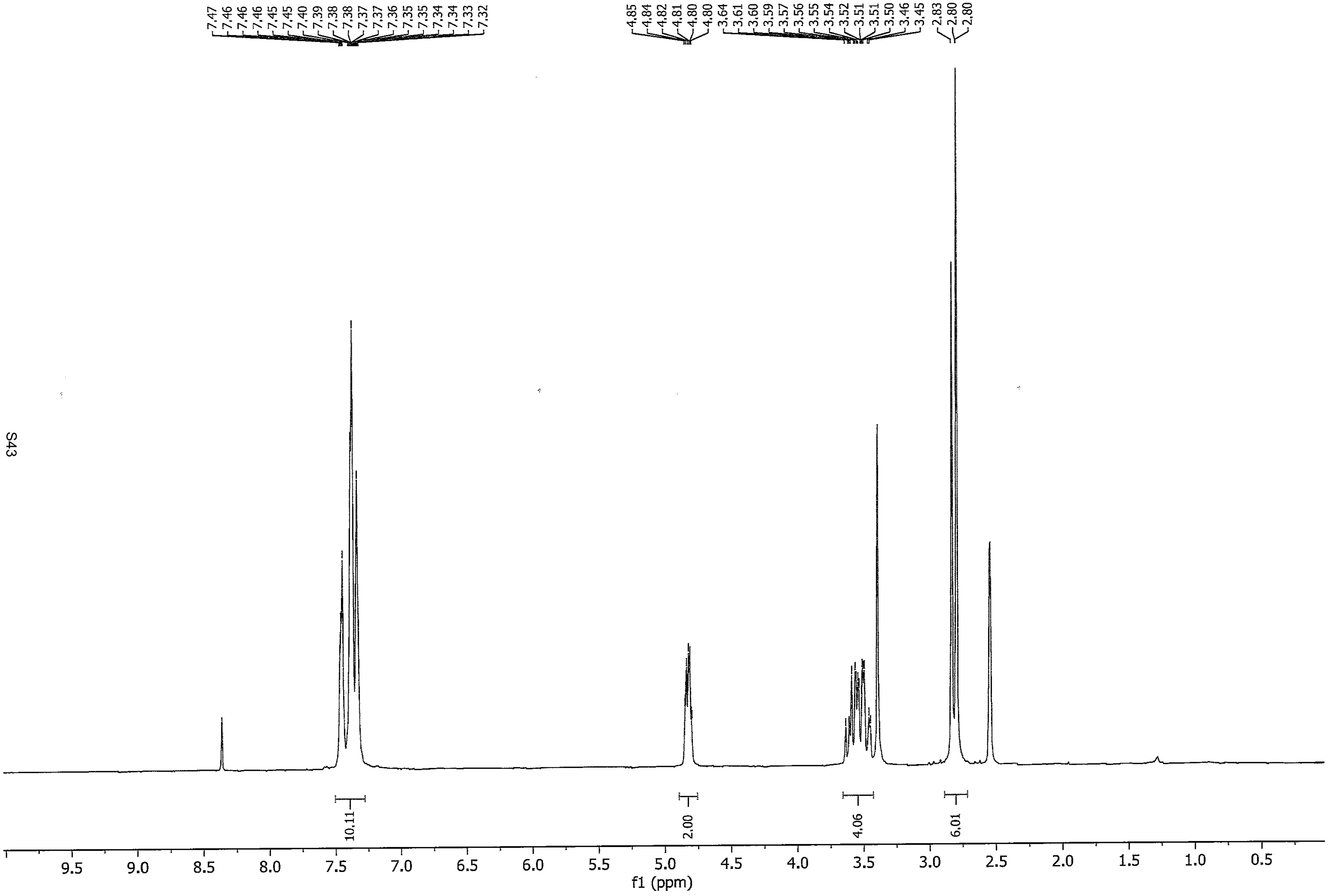

25 - methanesulfonyl_diadduct 13C

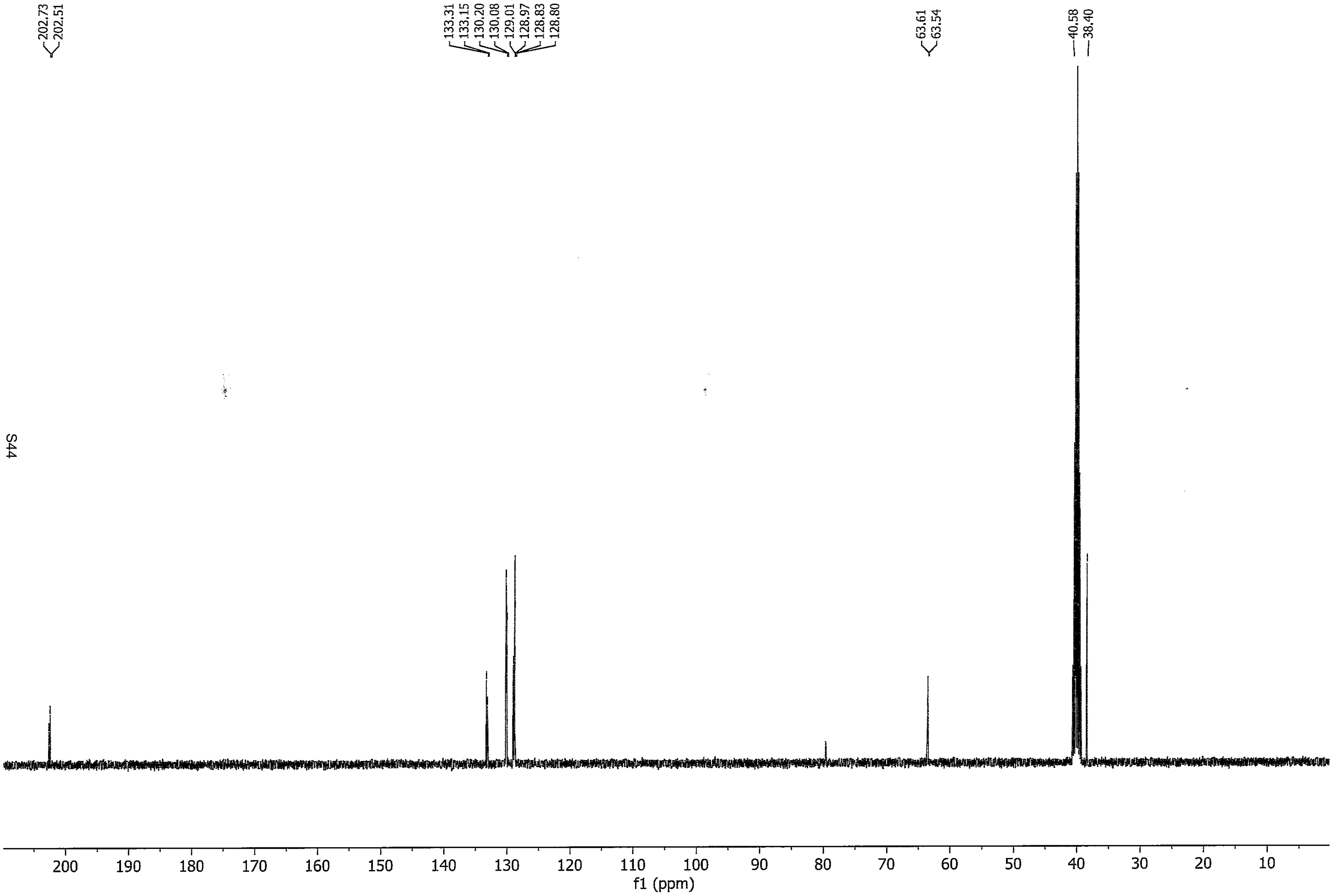

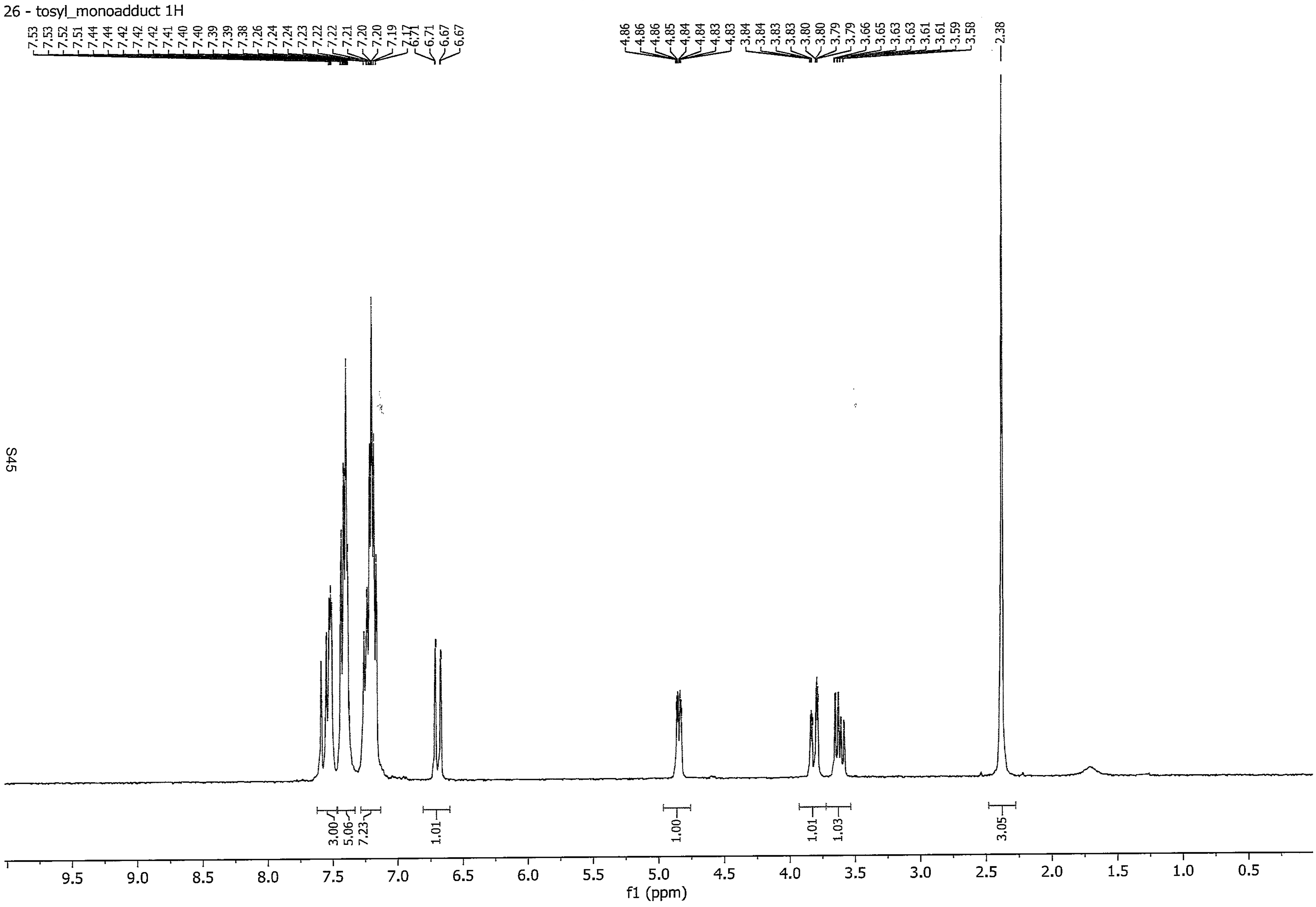
26 - tosyl_monoadduct 1H
7.53
7.53
7.52
7.51
7.44
7.44
7.42
7.42
7.42
7.41
7.40
7.40
7.39
7.39
7.38
7.26
7.24
7.24
7.23
7.22
7.22
7.21
7.20
7.20
7.19
7.17
6.71
6.71
6.67
6.67
4.86
4.86
4.86
4.85
4.84
4.84
4.83
4.83
3.84
3.84
3.83
3.83
3.80
3.80
3.79
3.79
3.66
3.65
3.63
3.63
3.61
3.61
3.59
3.58
2.38
3.00
5.06
7.23
1.01
1.00
1.01
1.03
3.05
9.5
9.0
8.5
8.0
7.5
7.0
6.5
6.0
5.5
5.0
4.5
4.0
3.5
3.0
2.5
2.0
1.5
1.0
0.5
f1 (ppm)

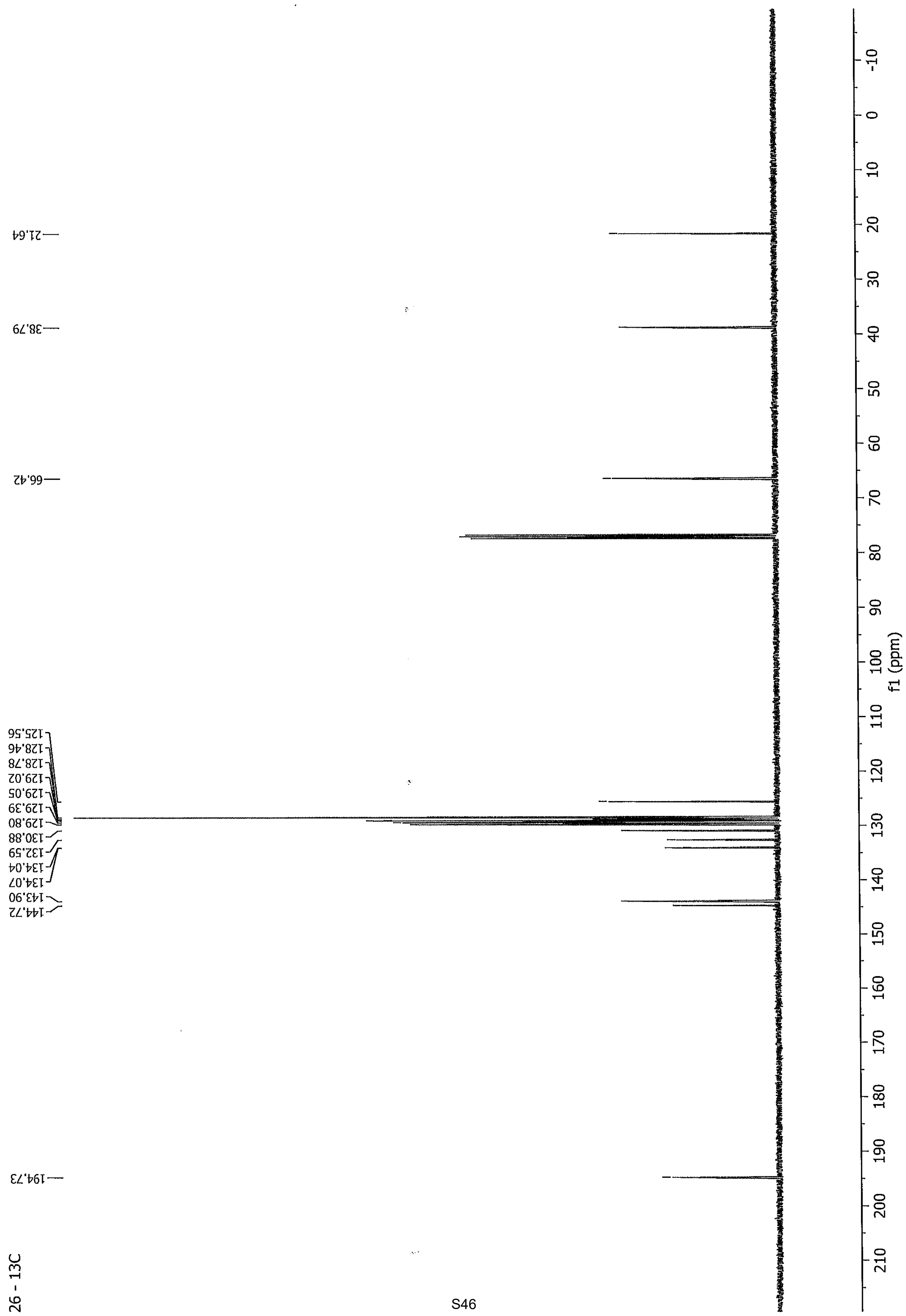
26 - 13C
194.73
144.72
143.90
134.07
134.04
132.59
130.88
129.80
129.39
129.05
129.02
128.78
128.46
125.56
66.42
38.79
21.64
f1 (ppm)
210
200
190
180
170
160
150
140
130
120
110
100
90
80
70
60
50
40
30
20
10
0
-10

27 - tosyl_diadduct 1H

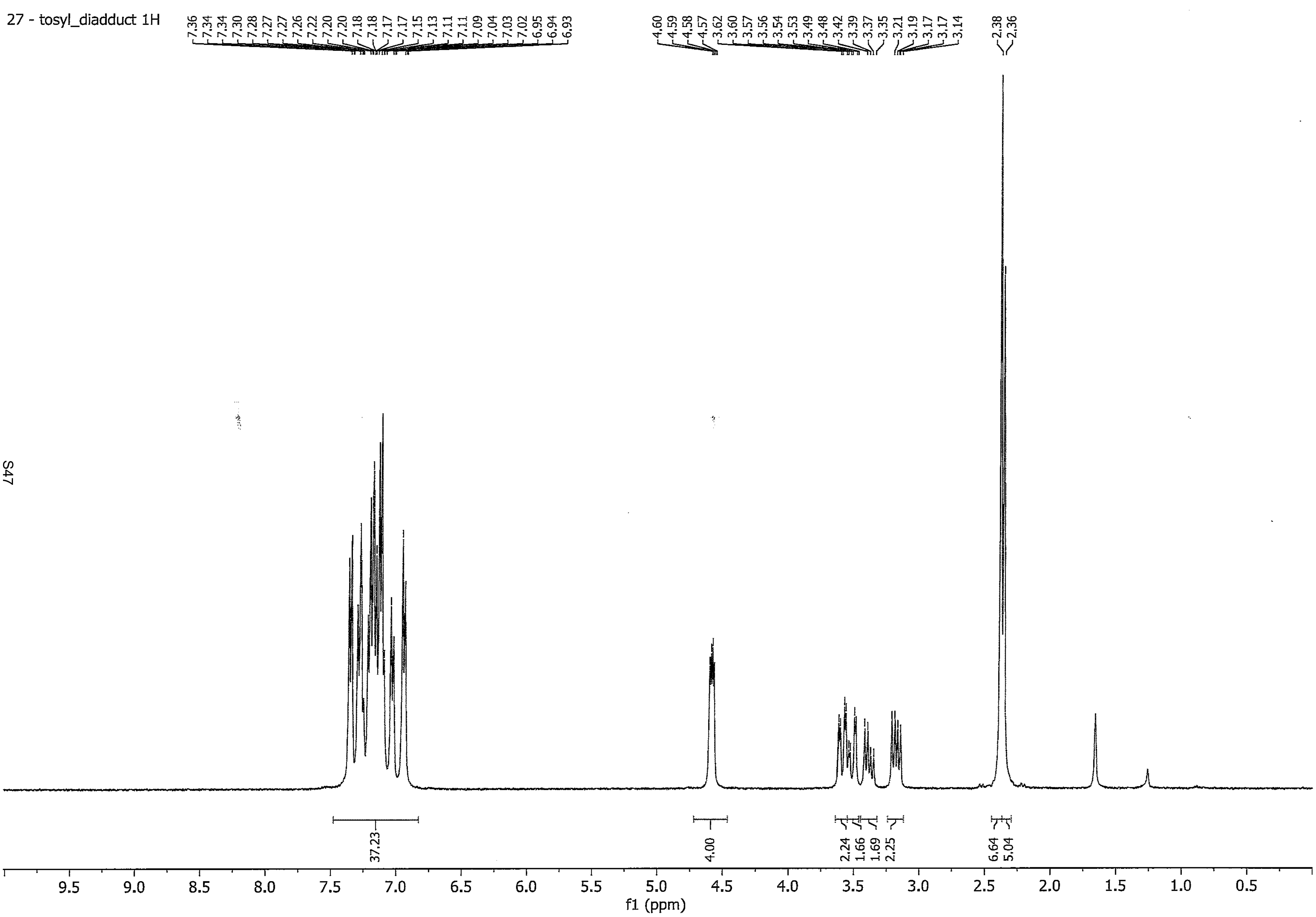

27 - tosyl_diadduct 13C

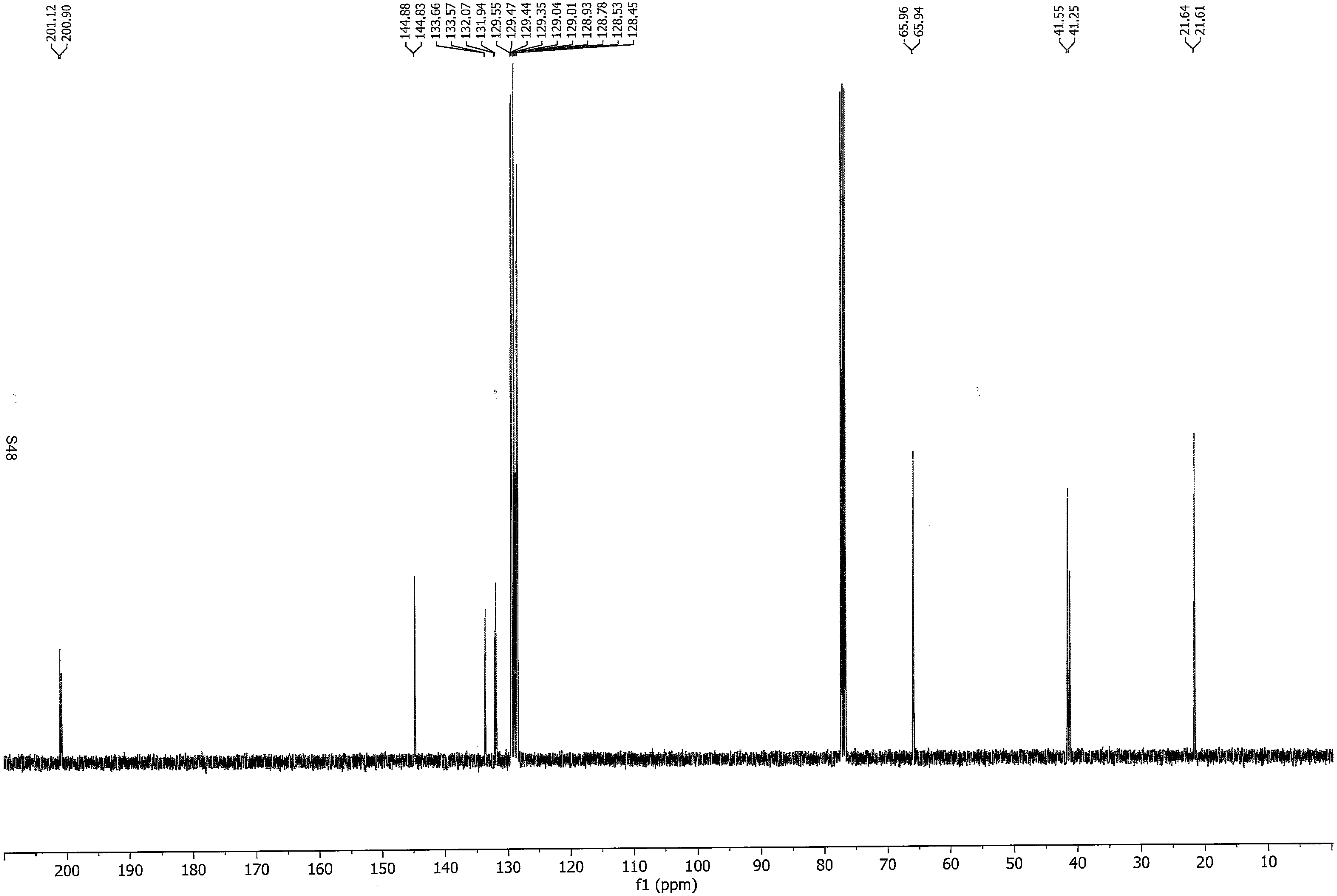

30 - chalcone_adduct 2.7:1 1H

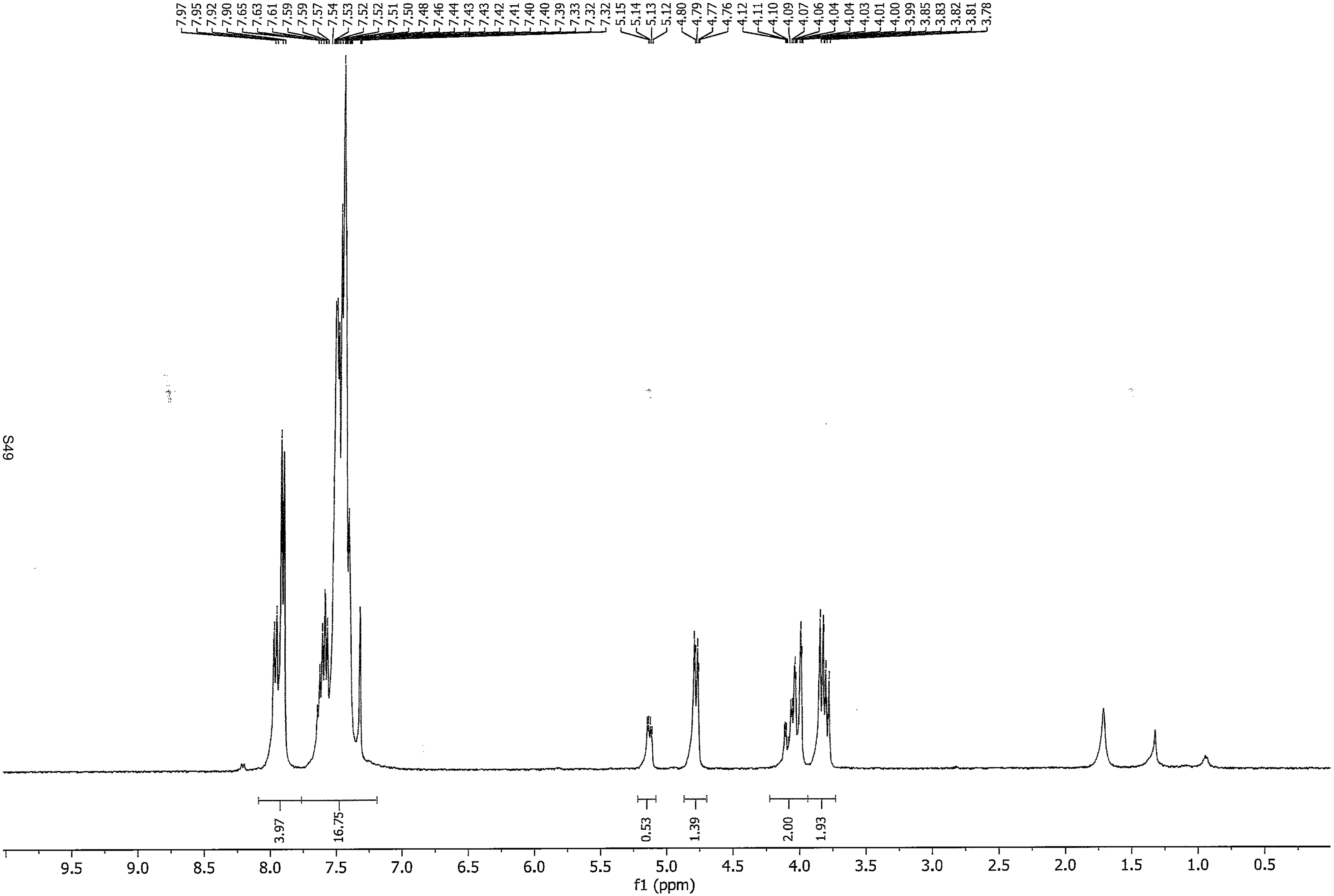

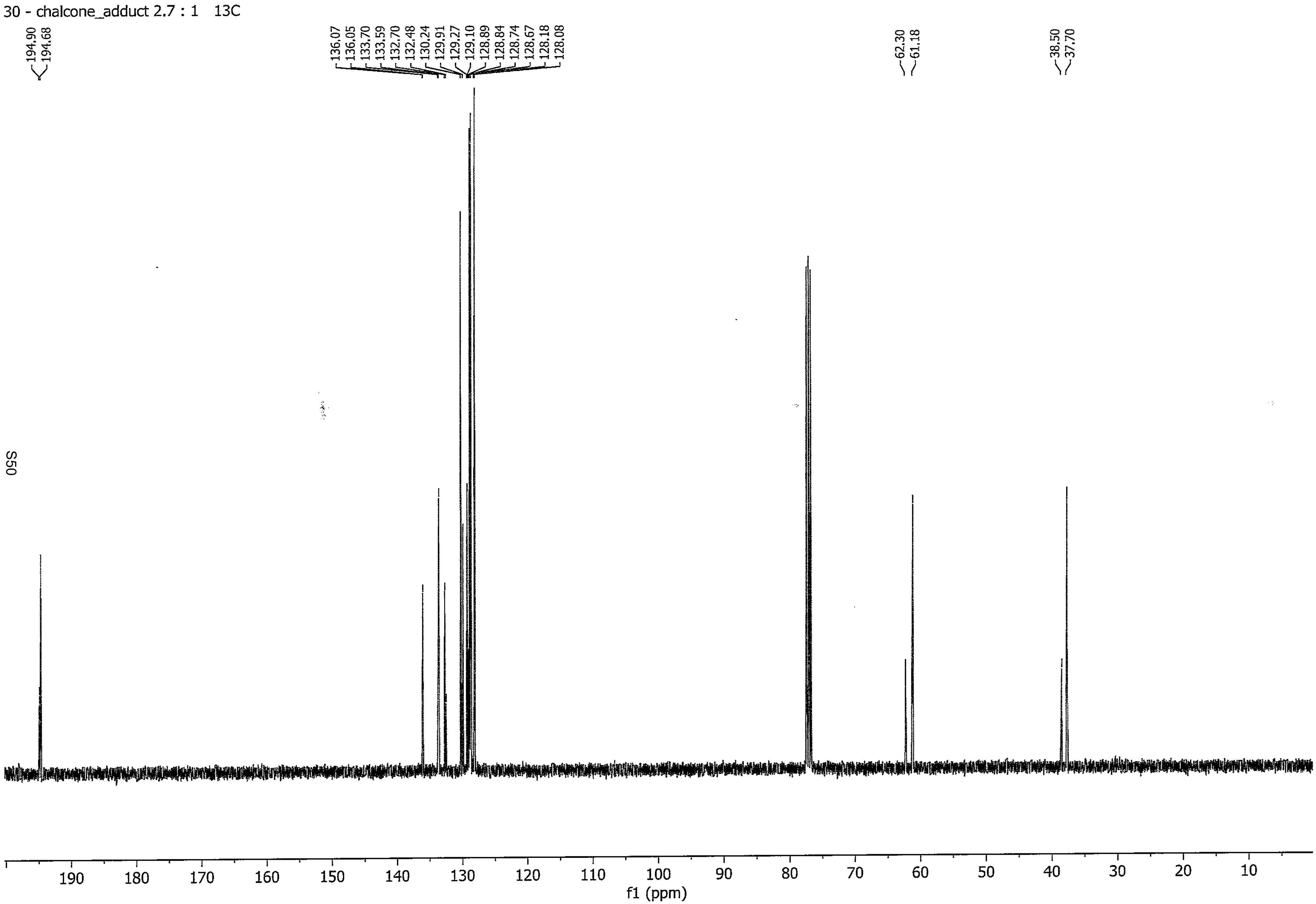
30 - chalcone_adduct 2.7 : 1 13C
194.90
194.68
136.07
136.05
133.70
133.59
132.70
132.48
130.24
129.91
129.27
129.10
128.89
128.84
128.74
128.67
128.18
128.08
62.30
61.18
38.50
37.70
190 180 170 160 150 140 130 120 110 100 90 80 70 60 50 40 30 20 10
f1 (ppm)

31 - benzalacetone_adduct 1H

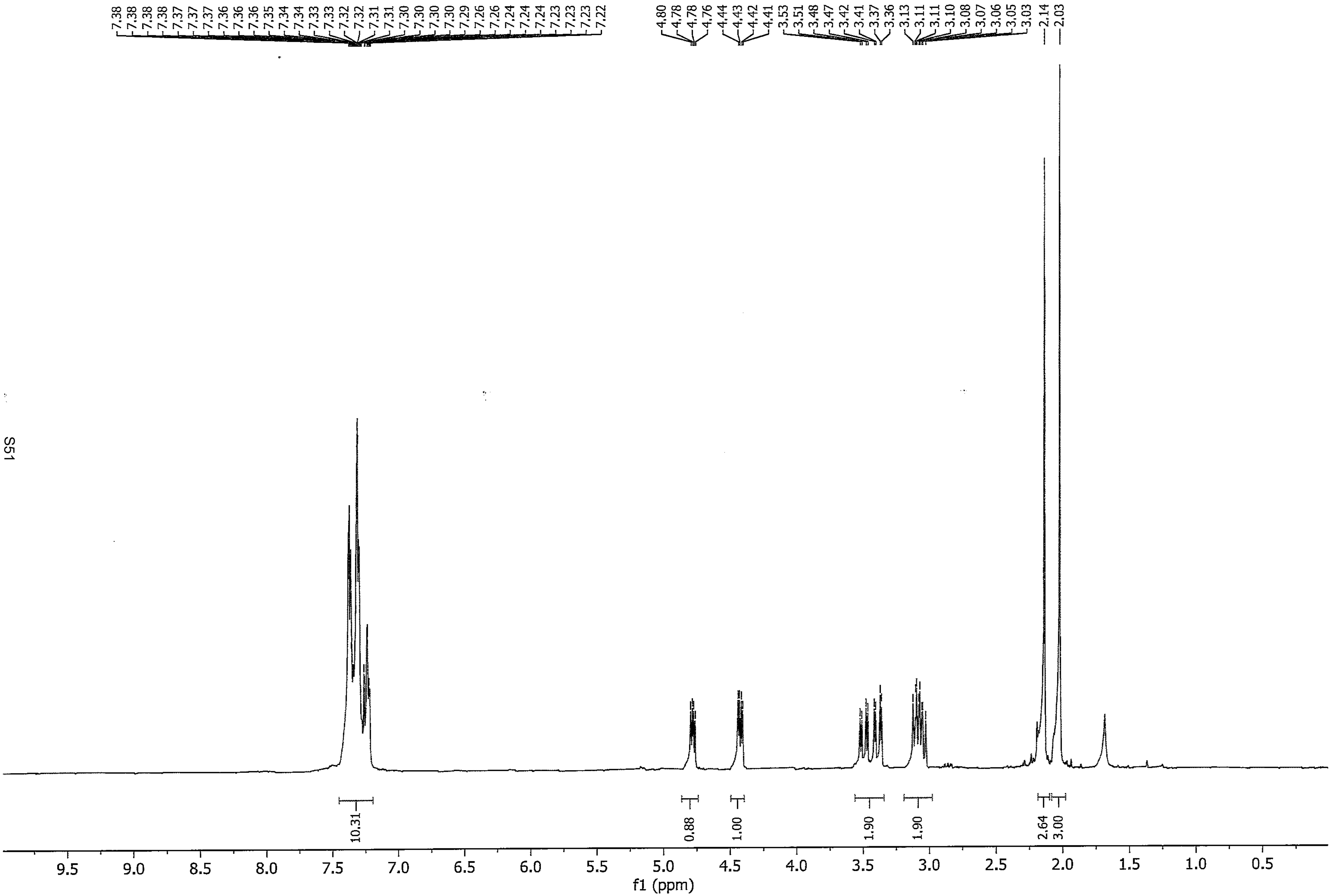

31 - benzalacetone_adduct 13C

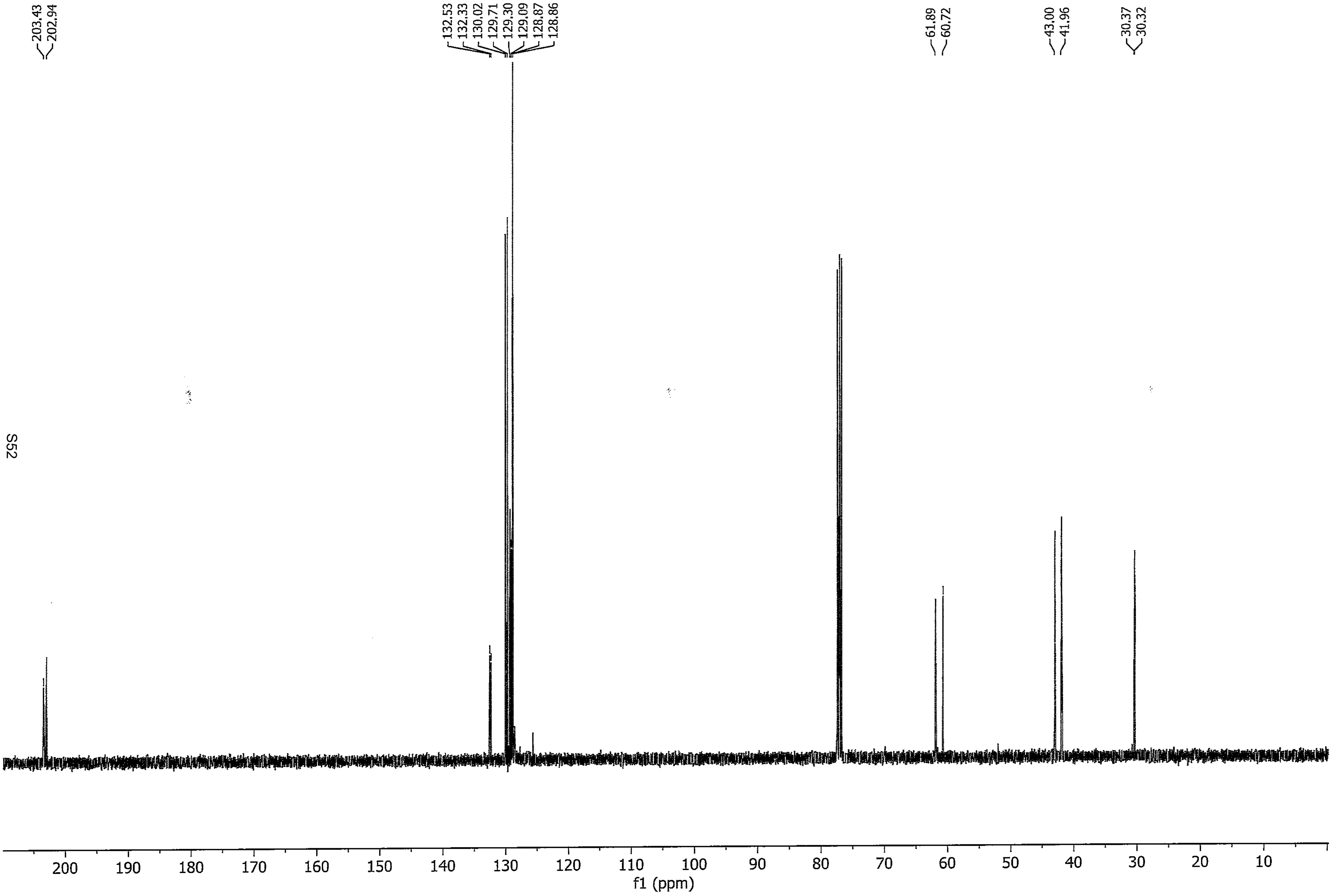

34 - 2-octen_3_one_adduct 1H

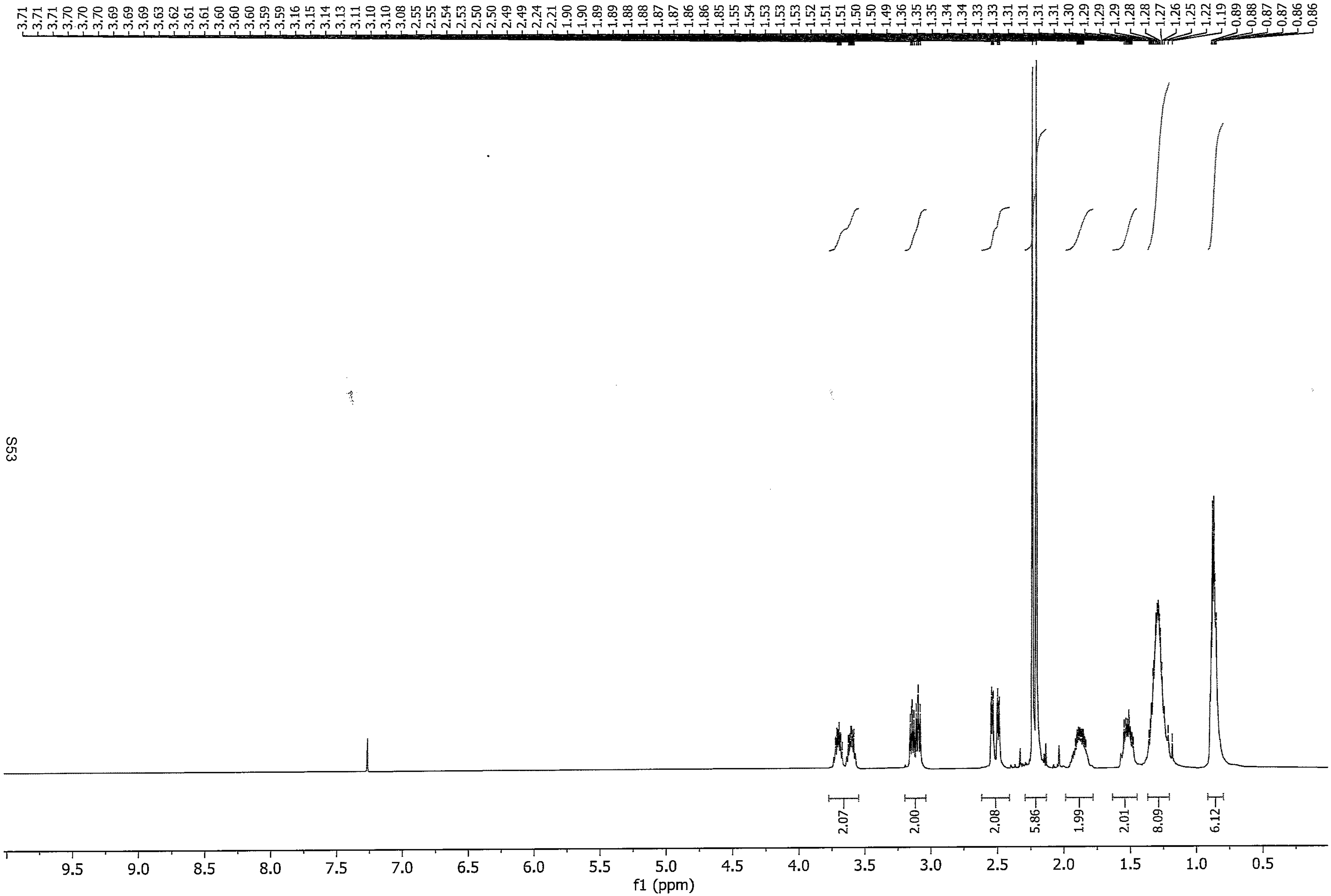

34 - 2-octen-3-one_adduct 13C

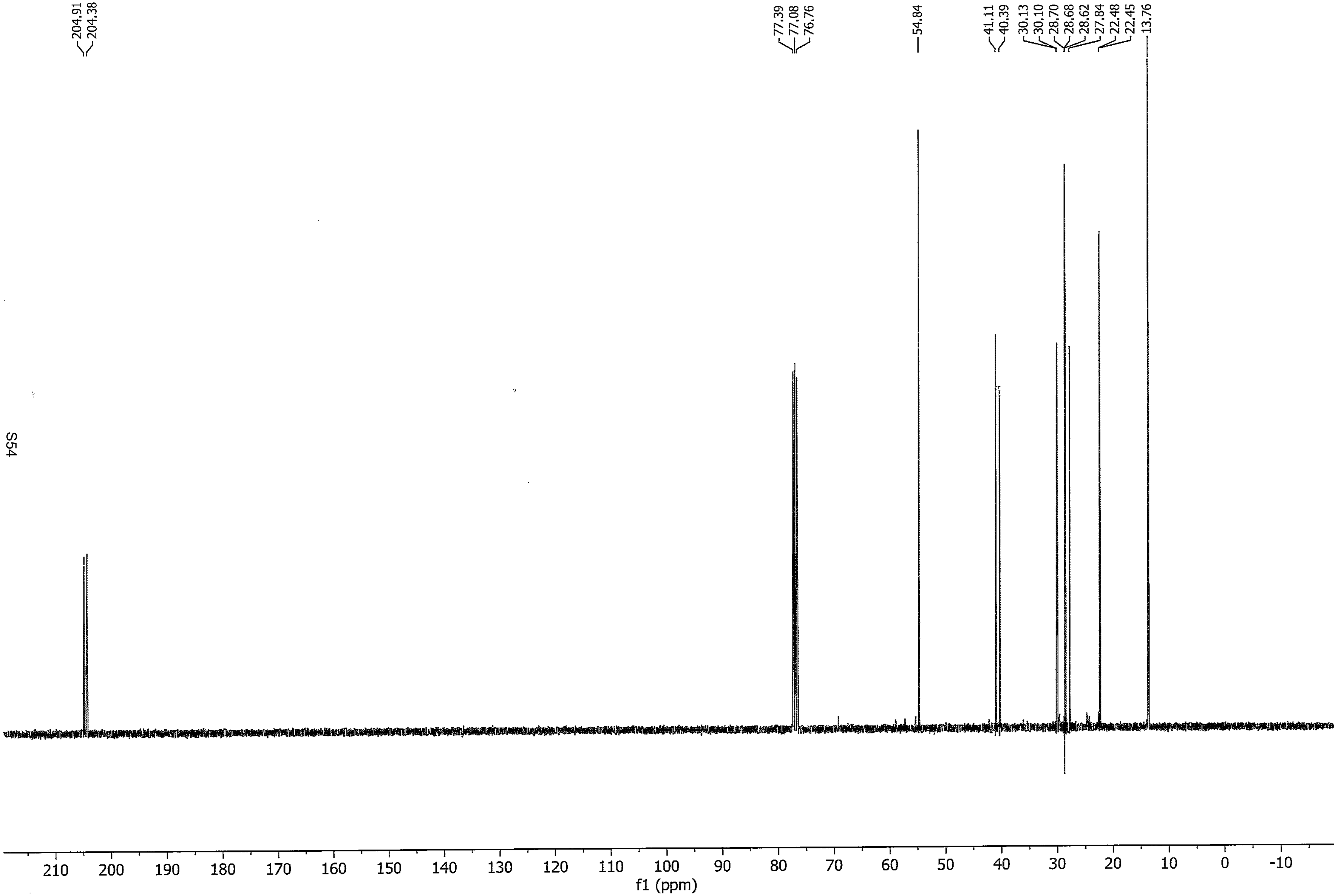

Supplement: File 1 — Experimental procedures, copies of 1H and 13C NMR spectra and further computational details. [file Beilstein_J_Org_Chem-22-742-s001.pdf]
